# Integrated analysis of fossils and molecular divergence time estimates a latest Jurassic origin of angiosperms

**DOI:** 10.1038/s41477-026-02311-x

**Published:** 2026-06-11

**Authors:** Ruolin Wu, Sandra Álvarez-Carretero, Yue Tong, Shan Wan, Harald Schneider, James Clark, Davide Pisani, Daniele Silvestro, Philip C. J. Donoghue

**Affiliations:** 1 Bristol Palaeobiology Group, School of Earth Sciences, University of Bristol, Bristol, UK; 2 Independent researcher, Hunan, China; 3 Center for Integrative Conservation & Yunnan Key Laboratory for Conservation of Tropical Rainforests and Elephants, Xishuangbanna Tropical Botanical Garden, Chinese Academy of Sciences, Menglun, China; 4 State Key Laboratory of Plant Diversity and Speciality Crops, Xishuangbana Tropical Botanical Garden, Chinese Academy of Sciences, Menglun, China; 5 Milner Centre for Evolution, Department of Life Sciences, University of Bath, Bath, UK; 6 Bristol Palaeobiology Group, School of Biological Sciences, University of Bristol, Bristol, UK; 7 Department of Biosystems Science and Engineering, ETH Zurich, Zurich, Switzerland; 8 Gothenburg Global Biodiversity Centre, Department of Biological and Environmental Sciences, University of Gothenburg, Gothenburg, Sweden; 9 Swiss Institute of Bioinformatics, Basel, Switzerland

**Keywords:** Plant evolution, Phylogenetics

## Abstract

Molecular timescales are based on the calibration of molecular evolution to geological time using fossil constraints, but conventional calibration strategies use limited and often subjectively interpreted fossil data. Here we used the Bayesian Brownian Bridge model to derive data-driven calibration densities on the basis of extensive fossil occurrence data. This approach integrates the uncertainty on extant and historical diversity to estimate clade age. We transformed the estimated ages based on >25,000 fossil occurrences into calibration densities, which we used to constrain 110 node ages in a 644-species angiosperm phylogenetic tree inferred from a molecular alignment of 83 genes. The results are incompatible with a post-Jurassic origin of angiosperms, instead inferring a short, Late Jurassic history. Our study demonstrates the utility of a mechanistic approach to establish node-age constraints in molecular-clock-dating analyses, resulting in a more objective method to integrate molecular and palaeontological data when inferring evolutionary timescales.

## Abstract (web summary)

Using extensive fossil occurrence data and Bayesian Brownian Bridge modelling, this study derives data-driven calibration densities for molecular clocks and suggests a Late Jurassic origin of flowering plants, indicating a cryptic early history of angiosperms.

## Main

Calibrating evolutionary trees to geological time is crucial for testing hypotheses on rates of evolution within lineages, coevolution and competition between lineages and the coevolution of Earth and life. While phylogenetic methods are used to understand taxonomic relationships, inference of evolutionary timescales from estimated branch lengths is challenging because rates and times are confounded. Bayesian approaches can be used to tease apart these evolutionary rates and clade ages by estimating them jointly if additional information is provided to calibrate either the rates or the branching times^1^. Node-age constraints have long been used to calibrate phylogenies to absolute time, on the basis of fossil evidence that provides minimum and/or maximum bounds on clade ages^2–4^. This node-calibration approach has proved controversial when clade age estimates are substantially older than the oldest fossil record^5^. One of the most iconic debates concerns the timing of divergence of the clade of living flowering plants (crown angiosperms), the origin and diversification of which underpins modern terrestrial ecosystems. Fossil evidence has been interpreted to reflect an Early Cretaceous origin of crown angiosperms, ~130–140 million years ago (Ma)^5^. However, Bayesian node-calibrated approaches under relaxed-clock models often estimate a Jurassic, Triassic or even Permian origin of the clade^6^, a discrepancy between fossil-based and molecular-clock time estimates that is commonly referred to as the Jurassic gap^7^. Debates over the veracity of the Jurassic gap have centred on the ability of molecular clocks to accommodate shifts in the evolutionary rates implied by the interpretations of fossil evidence^6,8,9^, though this may be because of the challenge of integrating sufficient fossil data to support this conjecture^10^. Traditional calibration strategies, whether node- or tip-based, have limitations in utilizing more than a small sample of the relevant fossil record. In tip-calibrated trees, incorporating a large number of fossils increases model complexity and computational demands, reducing tractability^11^. In contrast, node calibration considers all available fossil occurrences in the lineage leading to the node whose age is to be constrained, and the oldest is used to specify a sensible minimum age for that node. This strategy is often perceived as underutilizing the broader fossil record, as a probability density incorporating all fossil occurrences is not modelled^12^.

Here we propose a two-step, sequential approach using the Bayesian Brownian Bridge (BBB) model^13,14^ to estimate the timing of clade origination on the basis of all available fossil occurrence data. These estimates are then used as prior densities for node calibration in a molecular clock analysis of angiosperm diversification. BBB analyses are based on fossil stratigraphic occurrences and the extant diversity of clades, or their time of extinction. Clade age is estimated through a Markov chain Monte Carlo (MCMC)-based approach in which diversity is modelled as a random walk, scaffolded between the time of extinction or the present day. Diversity is required to equal or exceed sampled fossil diversity, and clade origin is estimated as the time at which a clade was composed of a single originating species. The BBB model does not use molecular data or the specification of priors on divergence times or evolutionary rates, and it is not tree-based except for the assumption that the analysed groups are monophyletic. These features and assumptions are attractive in that they obviate putative concerns with the birth–death priors underlying molecular clock analyses^15^ (though these concerns have been challenged^16^). While BBB estimates provide valuable fossil-based node-age constraints, they are applicable only to clades with a fossil record and can result in wide credible intervals due to the inherent uncertainty in fossil preservation and sampling. By integrating these estimates as node calibrations in a molecular clock framework, we obtain a complete time-calibrated phylogeny. Additionally, Bayesian timetree inference refines the initial BBB estimates by integrating them alongside molecular data, which can help reduce uncertainty and provide narrower credible intervals. BBB estimates of clade age thus serve both as an independent test of molecular estimates^13,14^ and as a mechanistic basis for establishing probabilistic fossil-based node calibrations in molecular clock analyses. BBB-based node calibrations also provide a means of including all pertinent fossil evidence, not just those that can be included as tip or conventional node calibrations. We explore the utility of our new approach using the fossil record and a large-scale molecular dataset for angiosperms, testing the Jurassic gap hypothesis while deriving a comprehensive and accurate timeline for the diversification of angiosperms.

## Results

### Age estimation from the BBB method

**Fig. 1:**
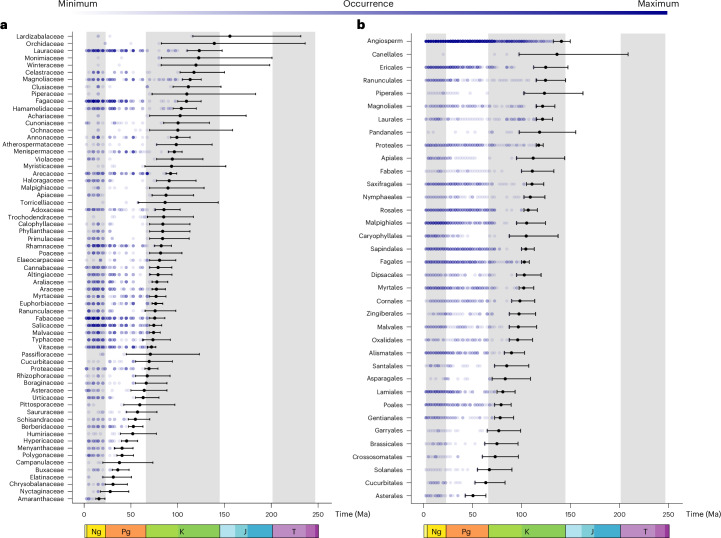
The fossil occurrence distribution and BBB-inferred age over time for all sampled clades. **a**,**b**, The distribution and age for the family level (**a**) and for the order and whole angiosperm level (**b**). The *y* axis lists the names of the sampled clades, while the *x* axis shows the geological time. Fossil occurrences are plotted in 2.5-Myr time bins, visualized as blue points with scaled colour transparencies alongside the *x* axis for each sampled clade. The transparency scale was calculated as log_10_(occurrences) for better visualization. The error bars show the 95% HPD intervals of the BBB analysis of each sampled clade, and black dots within the error bars indicate the estimated mean root age. Statistics were derived from the posterior distribution of the BBB analysis (*n* ≥ 3,600 posterior samples retained after burn-in for each clade).

The BBB is a mathematical model capable of generating clade age estimates without requiring an explicit phylogeny, aside from assuming monophyly of the taxonomic groups analysed. It estimates the time of clade origination through MCMC sampling of diversity through time. This is modelled as a random walk that is conditioned on being equal to or higher than sampled fossil diversity per unit time, and equal to the modern diversity of living clades. Clade origination is estimated to be the time when the lineage first had a diversity of one. We applied the BBB model to a dataset comprising 25,685 fossil occurrences, originally analysed by Silvestro et al.^13^. We excluded all pollen records from our analysis because they are difficult to attribute consistently to family-level clades within angiosperms, show disproportionately high diversity relative to meso- and macrofossil assemblages, cannot be directly linked to corresponding meso- or macroremains, and possess few corroborating apomorphies to discriminate homology from convergence. The dataset was revised in terms of the stratigraphic ages of the fossil occurrences and their systematic classification at different taxonomic levels. Instead of estimating the age of all angiosperm families and inferring the age of angiosperms on the basis of the age of the oldest family, as Silvestro et al.^13^ did, we estimated the ages of clades at different taxonomic ranks. Although taxonomic ranks are imperfect comparators, families and orders offer an effective balance between fossil sampling sufficiency and phylogenetic coherence, and they have been widely used in palaeobotanical studies. Thus, while we estimated the ages of families on the basis of species occurrences, the ages of orders were estimated on the basis of occurrence data for families, and the age of the angiosperm crown group was estimated on the basis of occurrence data for orders. By doing this, we estimated the ages of 101 clades using the BBB model, including 65 families, 35 orders and the crown-angiosperm clade (Fig. 1). The 95% highest posterior density (HPD) interval associated with each estimate represents the temporal interval, preceding the oldest fossil occurrence, within which the clade’s origination time is inferred to occur with 95% posterior probability. For each clade, we then inferred a minimum age estimate (corresponding to the age of the oldest fossil), a maximum age estimate (the upper bound of the 95% HPD interval) and a mean age estimate. Generally, a scarcity of fossil occurrences and a dispersed temporal distribution tend to result in broader and, consequently, more uncertain HPD intervals. All HPDs exceeding 100 million years were associated with clades with fewer than five fossil occurrences (Supplementary Table 1) and clades with a small number of fossil occurrences but a long stratigraphic range.

Estimating clade ages at different taxonomic ranks, the mean age projections spread across the Cenozoic (16 families, 24.6%; 2 orders, 5.7%), Cretaceous (48 families, 73.8%; 33 orders, 94.3%) and Jurassic (1 family, 1.5%). Among them, 11 families and 4 orders have uncertainties associated with their origination times that extend to before the Cretaceous, with either scarce or dispersed fossil occurrences (Fig. 1). For one family, Lardizabalaceae (Ranunculales; eudicots), the mean estimated origination age falls in the Jurassic. The total number of fossil occurrences associated with this family is four, and they span 100 Myr. Disregarding variations in sampling rates among clades by aggregating all angiosperm fossils results in an estimate for the origin of crown angiosperms that ranges from 150 to 133 Ma (Kimmeridgian–Valanginian; mean of 141 Ma) or Late Jurassic to Early Cretaceous (Fig. 1b and Supplementary Table 1).

### Molecular clock analyses

The BBB results represent fossil-based posterior estimates of clade age and, as such, may be readily employed as node calibrations in molecular clock analyses. Traditional approaches to node calibration typically rely on the stratigraphic age of a single fossil as the lower bound on a probability distribution that is otherwise informed by the subjective interpretation of qualified negative evidence^2,17^. This can include taphonomic controls afforded by the presence of older outgroup relatives in the absence of ingroup representatives^3,18^. In comparison, the BBB model provides an approach to comprehensively incorporating all available fossil evidence. The probabilistic clade age estimates from the BBB model can be directly used as calibration densities for molecular clock analysis, reducing subjectivity in the formulation of calibrations and providing a more comprehensive basis for calibrating specific nodes in a phylogenetic tree.

To explore how different assumptions about calibration uncertainty affect species divergence time estimation, we employed three calibration strategies: (1) a skew-T distribution that accurately reflects the probabilistic nature of BBB clade age estimates; (2) a uniform distribution set at the limits of the 95% HPDs from the BBB estimates, with soft bounds implemented as 2.5% tail distributions added to both the minimum and maximum bounds to reflect the uncertainty associated with the 95% HPD; and (3) a uniform distribution with hard bounds set at the limits of the 95% HPDs from the BBB estimates, imposing strict constraints on the clade age estimates inferred under the BBB model with the fossil occurrence data. These calibration strategies were designed to assess the sensitivity of angiosperm divergence time estimates to the construction of the joint time prior and how this relates to the posterior time estimates. This is of significance since it is widely appreciated that calibration densities (user-specified priors) may be altered when dating programs infer the marginal densities (effective priors) due to the additional topological constraints that impose age estimates for ancestral nodes that are older than those of their descendants^19^. Thus, while calibration strategy 1 provides for specified priors that faithfully reflect the BBB estimates, calibration strategies 2 and 3 constrain, by degree, the marginal densities (effective priors) to fall within the bounds of the BBB estimates.

**Fig. 2:**
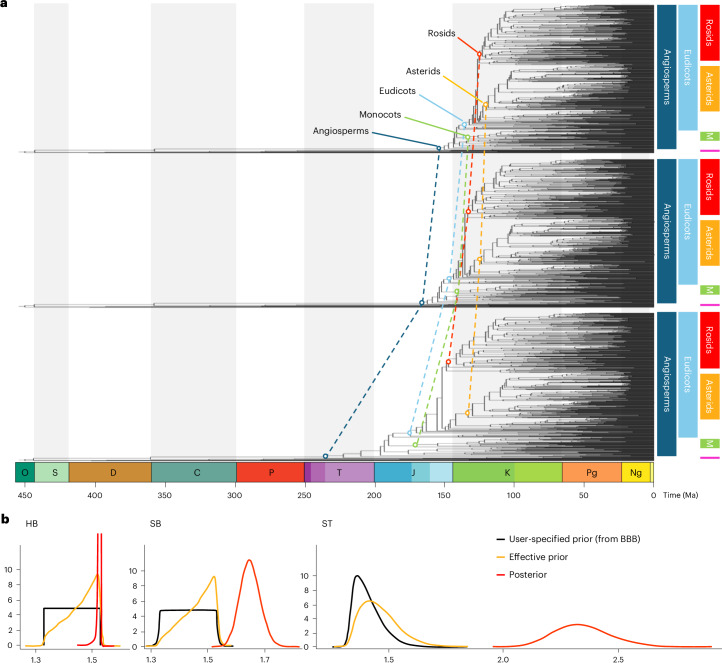
The impact that marginal densities (effective priors) resulting from calibration densities (user-specified priors) can have on posterior time densities of major groups of tracheophytes and angiosperms. **a**, Tracheophyte evolutionary timescale. The nodes of the five major groups are highlighted with circles using different colours: angiosperms in dark blue, monocots in green, eudicots in light blue, asterids in orange and rosids in red. The same colours are used for the dashed lines that connect the same node across the three evolutionary timelines inferred under three different calibration strategies: HB, SB and ST, ordered from top to bottom. Details of three trees can be found in Supplementary Fig. 2. **b**, Calibration densities (user-specified priors), marginal densities (effective priors) and posterior time densities for the angiosperm crown node (node label: 648). From left to right, these densities are displayed and compared for each of the evaluated calibration strategies: HB, SB and ST. Calibration densities are represented with black lines, marginal densities with yellow lines and posterior time densities with red lines.

While the marginal densities under different calibration strategies generally fully fall within the calibration densities, this is not always the case^20^. For calibration strategy 1, which used a skew-T distribution, the age of the peak of the density varied between marginal and calibration densities. For calibration strategies 2 and 3, which used uniform distributions with soft and hard bounds as calibration densities, respectively, the marginal densities were non-uniform and sometimes multimodal (Fig. 2b and Supplementary Fig. 3).

The posterior time estimates showed even greater differences from the specified calibration densities. Under calibration strategy 1, the divergence time estimate for the crown angiosperm spans almost 50 Myr from the Permian to the Triassic (261.26–212.30 Ma), with a Late Triassic mean time estimate. Crown monocots are estimated to have originated 187.49–153.25 Ma, and crown eudicots 186.95–163.51 Ma, both fully within the Jurassic. Within eudicots, rosids are estimated to have originated within a Late Jurassic–Early Cretaceous interval (154.96–139.73 Ma) and crown asterids within the Early Cretaceous (140.38–126.32 Ma).

**Fig. 3:**
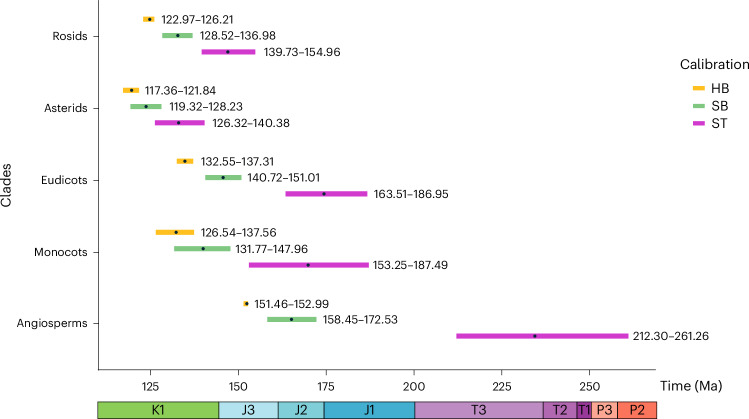
Inferred 95% HPD intervals for the divergence times of the five major nodes under different calibration strategies. Five major groups are listed on the *y* axis, while the timeline (absolute times) is shown on the *x* axis. The black dots in each of the 95% HPD intervals indicate the corresponding mean posterior divergence times. Each colour relates to a different calibration strategy: purple for strategy 1, the skew-T distribution (ST); green for strategy 2, the uniform densities with soft bounds (2.5% tail probability densities on both sides) (SB); and yellow for strategy 3, the uniform densities with hard bounds (HB).

The other two calibration strategies generated younger timescales for early angiosperm evolution. Molecular clock analyses using calibration strategy 2 estimated angiosperms to have originated in the Middle to Late Jurassic (172.53–158.45 Ma), crown monocots and crown eudicots in the latest Jurassic to Cretaceous, and rosids and asterids fully within the Cretaceous. Analyses using calibration strategy 3 estimated crown angiosperms to have originated in the Late Jurassic (152.99–151.46 Ma) and crown monocots, eudicots, asterids and rosids all originating in the Cretaceous (for comparison, see Fig. 3). Overall, the increasing constraint on node ages provided by calibration strategies 1–3 resulted in increasingly narrow HPDs, with uncertainty increasing progressively from the tips to the deepest nodes within the tree (Fig. 2a).

## Discussion

We present a timescale for angiosperm evolution based on the integration of tens of thousands of fossil occurrences and a large-scale genomic dataset, achieved using BBB to objectively define node calibrations for molecular-clock-dating analyses. Silvestro et al.^13^ used the BBB model to estimate the ages of families and of crown angiosperms on the basis of the age of the oldest family. This suggested that crown angiosperms originated in a Permian–Jurassic interval. However, this approach to interpreting the results of the analysis is biased by the oldest families, which have sparse fossil records and therefore extremely broad clade age uncertainties. When phylogenetic models were considered as the cause of the discrepancy between molecular clock and stratigraphic interpretations of the angiosperm diversification, it was an advantage that the original BBB study was aphylogenetic^13^, avoiding biases inherent in phylogeny-based analyses. However, the phylogenetic relationships among clades are informative of their ages since sibling lineages arise contemporaneously from their common ancestor and tree topology necessarily implies age constraints across its nodes. The uncertainty associated with sparsely sampled lineages can thus be strongly reduced when their sibling lineages are richly sampled and their BBB estimates of clade age are therefore more certain. Hence, in contrast to Silvestro et al.^13^, we estimated the age of higher clades among crown angiosperms on the basis of sampling the occurrences of lower taxonomic ranks. This resulted in much younger age estimates for key angiosperm clades, including Early Cretaceous estimates for the origins of rosids, asterids, eudicots and monocots, and a Late Jurassic estimate for the origin of crown angiosperms. These probabilistic estimates of clade age indicate that the fossil record of crown angiosperms is a close approximation of their time of origin.

**Fig. 4:**
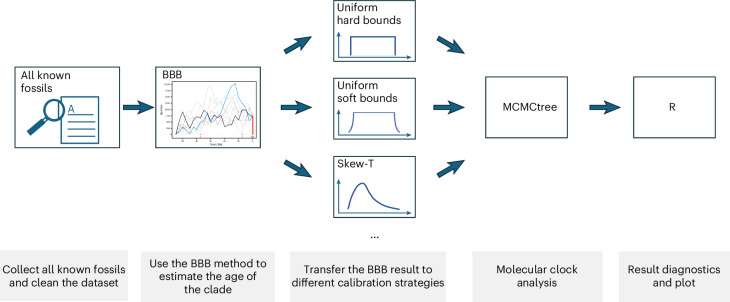
Workflow followed to derive node-age constraints based on the results obtained under the BBB approach, which were subsequently used to calibrate the fixed tree topology used in PAML for Bayesian timetree inference. The workflow includes five major steps: data collection, BBB analysis for clade age estimation, deriving node-age constraints as calibration strategies based on the already estimated clade age densities, Bayesian node-dating timetree inference and MCMC diagnostics. If the latter tests are not passed (for example, issues with chain convergence, severe truncation problems and so on), model parameters and/or MCMC settings may need to be adjusted and molecular-clock-dating analyses rerun.

Barba-Montoya et al.^21^ advocated for more mechanistic approaches to deriving probabilistic node age calibrations, and this is precisely what the BBB clade age estimates represent. We calibrated molecular clock analyses using BBB estimates but, in so doing, we explored how BBB-based calibrations are best implemented. Intuitively, the BBB estimates should be implemented faithfully, as non-uniform prior probabilities reflect the BBB posterior probabilities. However, specified time priors are transformed in the construction of the joint time prior to ensure that in clade age proposals to the MCMC process ancestral nodes are older than their descendants. This can lead to marginal densities that can be very different from the calibration densities specified by the user^19^. Hence, we implemented three different calibration strategies that imposed increasingly greater temporal constraint, diminishing the degree to which the marginal densities could depart from the calibration densities (the full workflow is shown in Fig. 4). We found that the least constrained calibration strategy (which is the most faithful to the BBB estimates) led to the most disparate marginal densities, invariably leading to older timescales. Only the most tightly constrained calibration strategy, in which the BBB posterior probabilities were converted to hard-bounded uniform distributions as calibration densities, led to posterior estimates that were confined to the bounds of the BBB estimates of clade age.

We reason that the third, most tightly constrained calibration strategy is the most appropriate because this is the only strategy that faithfully reflects the BBB-based clade age estimates in the joint time prior of the molecular clock analysis. Researchers have long been encouraged to review the marginal densities inferred when no data are being analysed to ensure that they are biologically reasonable and align with the calibration densities that reflect the evidence on which they were specified^19^. The acceptable extent of disagreement between calibration densities and marginal densities has been difficult to assess when the node calibrations are established on a qualitative analysis of the presence and absence of fossil records of the ingroup and outgroup^2,18,22^. However, the BBB-based estimates of clade age are formal mechanistically derived prior estimates of clade age, and we expect that clade age lies within these estimates, not least since simulations have shown the BBB model to be effective in accurately recovering clade ages even when fossil sampling rates are very low^13,14^ (Supplementary Fig. 5). We therefore consider calibration strategies that lead to marginal densities incompatible with the BBB-based specified priors as inappropriate. The molecular sequence data and phylogenetic framework add information that allows us to improve the precision of these estimates while also inferring an overall timescale that includes all lineages. This shows that conventional clock methods are entirely capable of recovering explosive radiations (contra Smith and Beaulieu^6^) when there is sufficient palaeontological evidence to inform the calibrations that provide local checks on rate variation.

**Fig. 5:**
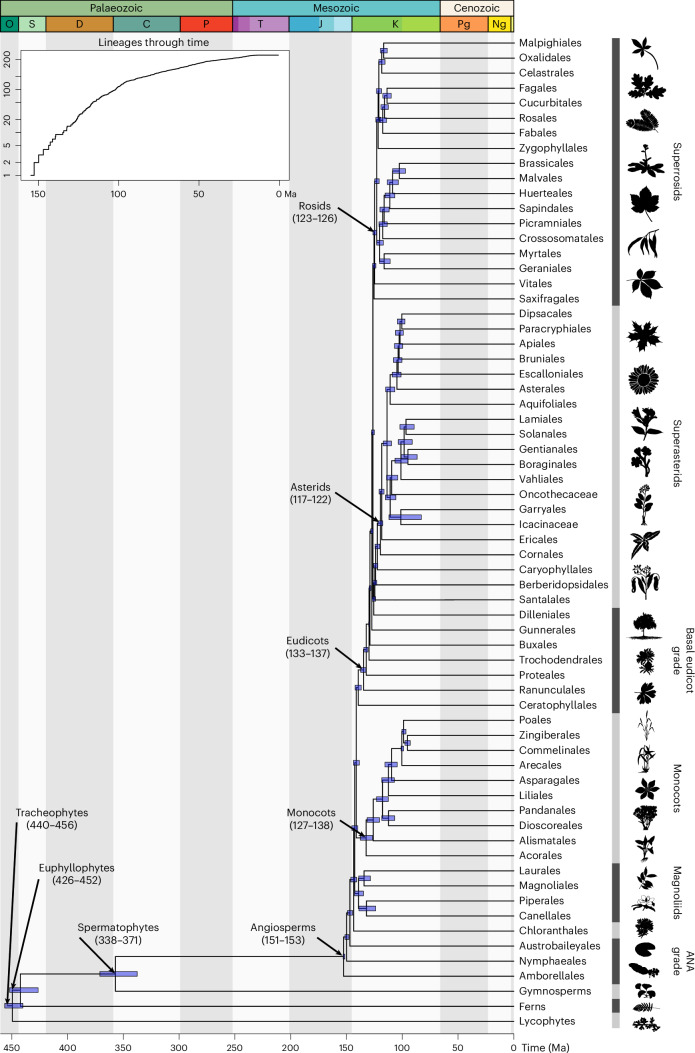
Estimated evolutionary timeline for tracheophytes using a uniform distribution with hard bounds encompassing 95% HPDs of major clades, and the lineages-through-time plot. The timetree of tracheophytes using strategy 3, densities in uniform distribution with hard bounds. The *x* axis shows the time (Ma), and for the lineages-through-time plot, the *y* axis shows the number of lineages. The blue bars show the 95% HPD intervals of the nodes. In the timetree, terminals are collapsed to represent angiosperm orders. Major nodes and clades are marked on the tree with arrows and shadow bars, respectively. Credit: plant icons, PhyloPic under a Creative Commons license CC0 1.0.

The resulting timescale for the origin of crown angiosperms (Fig. 5) is incompatible with previous molecular clock analyses that have inferred a deeper Jurassic, Triassic or Permian origin^6,23^, unless they were calibrated to ensure compatibility with an Early Cretaceous origin of angiosperms^24^. The main underlying difference between our analysis and previous ones lies in the nature of the calibrations. In particular, many have included broad and therefore largely uninformative prior age constraints on the angiosperm crown ancestor, so that they encompass older Jurassic and Triassic records that have little evidential basis for their claim on crown-angiosperm affinity^10^. Previous calibration schemes have also relied on the qualitative interpretation of absence data that have necessarily led to broad calibration priors. The BBB-based calibrations are considerably more informative, thus resulting in a much more precise timescale of angiosperm diversification.

This timescale disagrees with traditional palaeobotanical interpretations that rely on a literal reading of the fossil record, inferring angiosperms to have originated in the Valanginian (Early Cretaceous) on the basis of the earliest unequivocal record (for example, Friis et al.^25^ and Herendeen et al.^5^). The result that we have obtained through combined statistical analysis of the fossil record and molecular divergence time estimation indicates that crown angiosperms originated in the middle of the Late Jurassic. Many molecular clock analyses have led to diverging interpretations of the evolutionary history of angiosperms^26^. In contrast, our estimated timescale may not require much reformulation of the evolutionary narrative since it models the gaps in the fossil record of early angiosperm evolution. Independent lines of evidence have likewise supported a Late Jurassic origin of crown angiosperms. For example, Coiro et al.^8^ inferred a Late Jurassic origin of crown angiosperms on the basis of the biogeographic distribution of the earliest pollen records of the clade, conjecturing that Valanginian records of reticulate monosulcate pollen might reflect the diversification of crown mesangiosperms, rather than crown angiosperms. Perhaps most significantly, the overall timescale inferred here and its associated lineages-through-time plot (Fig. 5) suggest that the richness of angiosperm diversity emerged in the Early Cretaceous. This finding precedes the perceived angiosperm terrestrial revolution^27^, which has been linked to the increasing angiosperm richness but which may instead reflect the diversification of insect, fern and vertebrate clades in response to angiosperm family-level diversification, or else co-diversification with angiosperms at higher ranks.

Overall, our study shows that it is possible to formally integrate all relevant fossil evidence into molecular clock analyses and thus to derive informative calibrations even for clades with sparse fossil records, and in the absence of extensive morphological data^28^. In the case of angiosperms, molecular clock analyses calibrated in this way have resulted in a timescale that does not imply gaps in the fossil record of angiosperm diversification that are implausible given the nature and structure of their pollen record. We anticipate that the application of our approach could help resolve other iconic rock–clock conflicts, including the timing of the origin of placental mammals and animals. All of these controversies are associated with long stem lineages in which the body plans and genomes of their crown clade were assembled piecemeal over time. If our angiosperm timescale is a guide, younger estimates of crown-clade age only deepen the mystery of their evolutionary origins by further extending the length of their stem lineage.

## Conclusion

We introduced an approach for integrating the wealth of the fossil record into molecular clock analyses. By applying a Bayesian model to a revised angiosperm fossil occurrence dataset, we generated clade age estimates across different taxonomic levels, which were subsequently used as node-calibration densities to infer a time-calibrated phylogeny, reconstructing the origin and divergence history of angiosperms. We estimated a Late Jurassic origin for crown-group angiosperms, dramatically diminishing the perceived Jurassic gap between the molecular clock and the oldest unequivocal crown-angiosperm fossils. This revised timeline aligns with biogeographic inferences derived from early angiosperm fossil records and suggests that angiosperm diversification began to accelerate in the Early Cretaceous.

## Methods

### Molecular and fossil data collection

The molecular data in our study were assembled by Barba-Montoya et al.^29^, composed of 83 genes (75,030 nucleotides) spanning 644 species (632 angiosperms, 8 gymnosperms, 2 ferns and 2 lycophytes). The topology was based on the best-scoring maximum-likelihood tree inferred by Barba-Montoya et al.^29^ using a molecular dataset partitioned into five alignment blocks: first and second codon positions for plastid genes, third positions for plastid genes, first and second codon positions for mitochondrial genes, third positions for mitochondrial genes, and nuclear RNA genes. Phylogeny inference took place in RAxML v.7.7.8 (ref. ^30^) under the GTR + Γ model with 100 bootstrap replicates.

We chose to use this dataset because it encompasses the breadth of angiosperm diversity and has previously been subjected to extensive experimental investigation in terms of how it informs on the timescale of angiosperm diversification^29^. Other, larger datasets are available (for example, Zuntini et al.^31^), but these are neither necessary nor desirable to achieve our aim: exploring the utility of the BBB model to establish node calibrations for molecular clock analyses. These larger datasets are not desirable because their large size precludes experimental perturbation and, consequently, a thorough exploration of alternative calibration strategies, for reasons of computational tractability. The limits of increasing dataset size can also be established on the basis of the infinite sites theory, which shows that even with endless sequence data, errors in divergence time estimates persist because of inherent uncertainties in the fossil calibrations and variation in evolutionary rates^32^. These practical limits on molecular dating inform on the limit at which gains are still possible through adding sequence data.

To determine whether our dataset was sufficient in terms of alignment size to achieve precision in our divergence time estimates, we conducted a series of experiments in which we randomly sampled our sequence alignment to create data subsets consisting of 10%, 25%, 50% and 75% of sequences. We then carried out five replicate molecular-clock-dating analyses under the preferred calibration strategy. Across all sampling levels, the mean time estimates for major angiosperm nodes remained stable, whereas the widths of the corresponding HPDs increased predictably with decreasing sequence length (Supplementary Fig. 6). The infinite-sites plots show that the posterior uncertainty associated with the estimated age of crown angiosperms and other major clades approaches an asymptote within subsets of the alignment. These results indicate that the size of our full dataset has already exceeded the limit at which additional sequence data is likely to yield gains in precision when estimating the divergence time of these deep nodes.

We revised the fossil data assembled by Silvestro et al.^13^, a comprehensive dataset containing 25,685 fossil angiosperm occurrences. All pollen records were removed from our analysis. After revising the taxonomic classification and geological age of records in the dataset, we assigned fossil clades to the tree topology. Lastly, we established 101 node calibrations for key clades in the phylogeny to ensure that local checks on rate variance were evenly distributed throughout the tree.

### BBB

We identified 110 nodes in the fixed tree topology for timetree inference; these represent 65 families, 35 orders, the crown-angiosperm node and 9 outgroup nodes. The outgroup nodes were calibrated with the same node-age constraints used by Barba-Montoya et al.^29^, conventional node calibrations established after following best practice^17^. The remaining 101 ingroup angiosperm nodes were calibrated using node-age constraints derived from the results obtained under the BBB method^13,14^.

BBB is an open-source mathematical method for estimating the origin of clades solely on the basis of fossil occurrence and the modern diversity of the clades of interest; no molecular data are used, and phylogenetic constraints are not imposed. It has previously been validated using simulation studies, as reported in the original and subsequent research^13,14^. The Brownian bridge traces the diversity through time from modern diversity back to the time at which diversity was one, while constraining the estimated diversity per unit of time to ensure that it does not drop below the fossil sampled diversity. We controlled the sampling rate heterogeneity across different taxonomic levels by sampling in various units when sampling from different taxonomic levels (that is, sampling families using species as a unit, sampling orders using the family as a unit and sampling the crown angiosperm using the order as a unit).

We assessed MCMC convergence of the BBB analyses using Tracer v.1.7.2 (ref. ^33^). For analyses of all taxonomic levels, each was run for at least 1,000,000 to up to 5,000,000 iterations, sampling every 250 iterations. The first 10% of samples were discarded as burn-in to ensure that the retained samples approximated the stationary posterior distribution. Trace plots showed adequate mixing and stationarity for the time estimation, and effective sample size values exceeded 200 for it.

Specifically, for crown angiosperms, we explored alternative treatments of orders with uncertain taxonomic placement. We considered two extreme strategies: (1) treating all unknown orders as belonging to a single order and (2) treating each unknown order as a distinct lineage. To assess the robustness of the results, we performed ten independent random resamplings to generate occurrence matrices under each strategy. The resulting age estimates were highly consistent across replicates and treatments, with inferred crown ages ranging approximately from 135 to 153 Ma. Given the similarity of the results, we adopted the more conservative strategy, in which all unknown orders were treated as a single order. For this configuration, we retained only MCMC chains with effective sample size values above 200 for the age parameter. The final posterior density was obtained by combining the retained chains, and the 95% HPD interval was used to summarize the crown age estimate.

We derived three different calibration strategies based on the clade age densities obtained from the BBB results: (1) skew-T distributions, (2) uniform distributions with soft bounds (that is, tail probabilities of 2.5% at each side) and (3) uniform distributions with hard bounds. All densities were derived on the basis of the 95% posterior distribution of the densities obtained under the BBB analysis. These three calibration strategies were used alongside other node-age constraints based on the fossil record as calibration densities (user-specified priors); their impact on divergence time estimation was assessed. Before running a Bayesian timetree inference when the target distribution is the posterior, we ran various tests when the target distribution where samples were collected was the prior to assess whether there was discordance between the calibration densities and the marginal densities (effective priors). After making sure that the marginal densities were not in conflict with our calibration densities, we used 110 node-age constraints under both strategies 2 and 3 and 107 under strategy 1. These node-age constraints include 101 calibration densities derived from the BBB analyses; for strategy 1, three node-age constraints were excluded because they showed severe conflicts with the resulting marginal densities of neighbouring nodes. The same set of calibration densities to constrain the node ages of outgroups, following Barba-Montoya et al.^29^, was used across all three strategies. The list of calibration densities in different strategies is presented as well as the other related information (Supplementary Tables 2–3 and Supplementary Fig. 1).

### Bayesian node-dating timetree inference

All Bayesian timetree inference analyses were conducted in MCMCtree, part of the PAML package (v.4.10.7)^34^. The three calibration schemes were run separately with three partitioning schemes (first and second codon positions for plastid genes, first and second codon positions for mitochondrial genes, and nuclear RNA genes, with the two partitions for third codon positions in plastid and mitochondrial genes excluded). Our analyses used the independent rates model under a log-normal distribution^35,36^ and the HKY85 + Γ5 substitution model^37^; the independent rates model does not assume rate heritability. A gamma rate prior *G*(2, 50), centred around 0.04 substitutions per site per time unit, was used, and violation on the clock was assumed, thus assigning *G*(2, 4) as a prior on rate variance.

We ran a total of 32 independent chains for each calibration strategy under the independent rates model when the target distribution was the posterior, discarding a total of 50,000 iterations as part of the burn-in phase and sampling every 250 iterations. In total, each independent chain was set to collect 20,000 samples. MCMC diagnostics were carried out for each independent chain. Under each calibration strategy, the convergence plot of the posterior densities inferred for each model parameter is shown in Supplementary Fig. 4. No further samples had to be discarded from the trace file generated by MCMCtree; the burn-in phase specified in the control file was enough to retain those samples that are probably coming from our desired target distribution. We also observed healthy traces for each model parameter (that is, no issues with mixing or autocorrelation; thinning by increasing the sampling frequency was sufficient), and the effective sample size for each model parameter was above 200.

When evaluating the marginal densities when no data were used, we ran a total of four independent chains when the target distribution was the prior. We used the same MCMC settings as described above, and we also followed the same workflow when running MCMC diagnostics.

### Reporting summary

Further information on research design is available in the Nature Portfolio Reporting Summary linked to this article.

## Supplementary information

Supplementary Information Supplementary Sections 1–9.

Reporting Summary

## Data Availability

The generated outputs and associated documentation are available via GitHub at https://github.com/lynnwrl/angiosperms_dating.

## Code availability

The code and associated documentation are available via GitHub at https://github.com/lynnwrl/angiosperms_dating.
