# Supplementary material for: Integrated analysis of fossils and molecular divergence time estimates a latest Jurassic origin of angiosperms
Source: Nat Plants. 2026 Jun 11;12(6):1242–51. doi: 10.1038/s41477-026-02311-x (PMC13286982; doi:10.1038/s41477-026-02311-x)

nature plants

Supplementary information https://doi.org/10.1038/s41477-026-02311-x

Integrated analysis of fossils and molecular divergence time estimates a latest Jurassic origin of angiosperms

In the format provided by the authors and unedited

Supplementary Table 1. Summary of the results of BBB estimation

101 clades were analysed. The catagory 'group' refers to: 1. Angiosperm, 2. Orders, 3. Families. Estimated parameter values are summarised from the original BBB output file ("output.txt" file) and rounded to two decimals (when applicable). The R package "HDInterval" was used to calculate the corresponding 95% HPD intervals and the mean and mode for all parameter estimates. To learn more about the BBB workflow and familiarise with input/output files, please check the available documentation on the "rootBBB" GitHub repository: https://github.com/dsilvestro/rootBBB/tree/master.

group	name	likelihood	Nobs	Nfossils	root_obs	ext_obs	DA_count	root_est	root_m	root_M	root_est_mode	q_est	q_m	q_M	q_est_mode	a_est	a_m	a_M	a_est_mode	sig2_est	sig2_m	sig2_M	sig2_est_mode
1	Angiosperm	-160.12	64	1234	136.11	2.50	999.99	140.78	132.50	149.83	140.96	0.17	0.01	0.37	0.09	1.51	0.64	2.43	1.44	2.48	0.51	4.52	2.38
2	Alismatales	-53.62	14	93	82.50	2.50	1000.00	89.78	82.50	103.14	86.28	0.16	0.00	0.39	0.03	1.76	0.00	3.80	1.30	-0.03	-3.40	3.38	0.23
2	Apiales	-39.50	7	35	95.00	5.00	1000.00	111.96	95.00	144.16	100.45	0.03	0.00	0.13	0.00	2.52	0.36	4.71	2.48	0.95	-2.94	4.89	2.70
2	Asparagales	-25.57	14	12	67.50	7.50	1000.00	83.41	70.00	109.18	73.98	0.01	0.00	0.03	0.00	1.38	0.00	3.41	0.38	3.87	1.30	6.22	4.28
2	Asterales	-23.53	11	22	42.50	2.50	1000.00	50.44	42.50	63.62	46.38	0.06	0.00	0.18	0.02	2.90	0.00	6.76	0.81	2.08	-1.20	5.31	2.39
2	Brassicales	-25.91	17	19	62.50	2.50	1000.00	74.93	62.50	96.50	67.20	0.02	0.00	0.06	0.00	2.47	0.00	5.22	1.97	2.90	-0.76	6.05	3.67
2	Canellales	-15.00	2	4	97.50	20.00	1000.00	136.28	97.50	208.90	110.05	0.01	0.00	0.03	0.00	0.72	0.00	1.90	0.16	1.46	-2.03	4.27	2.56
2	Caryophyllales	-38.21	38	44	87.50	2.50	1000.00	104.84	87.52	137.38	93.47	0.01	0.00	0.03	0.00	3.21	0.61	5.57	2.90	2.48	-0.91	6.06	3.26
2	Cornales	-47.83	7	53	90.00	2.50	1000.00	98.37	90.00	113.53	93.76	0.10	0.00	0.28	0.02	1.19	0.00	2.86	0.27	1.18	-2.19	4.80	1.47
2	Crossosomatales	-24.03	7	16	60.00	5.00	1000.00	73.12	60.00	96.86	64.81	0.02	0.00	0.08	0.00	3.35	0.04	6.62	2.70	2.29	-1.55	5.39	3.17
2	Cucurbitales	-24.35	8	18	52.50	5.00	1000.00	63.50	52.51	83.25	56.80	0.02	0.00	0.09	0.00	3.11	0.00	6.62	2.60	3.06	-0.11	5.80	3.89
2	Dipsacales	-37.69	2	42	95.00	2.50	1000.00	102.87	95.00	120.02	96.48	0.28	0.00	0.61	0.18	1.99	0.27	3.66	1.84	-3.41	-6.26	-0.73	-3.25
2	Ericales	-80.81	22	185	112.50	2.50	1000.00	124.74	112.50	147.45	116.87	0.03	0.00	0.10	0.00	2.90	1.19	4.39	2.96	2.69	-0.85	5.50	2.78
2	Fabales	-38.58	4	34	100.00	2.50	1000.00	111.06	100.00	133.08	103.63	0.11	0.00	0.30	0.02	1.31	0.00	2.99	0.45	-0.93	-4.72	3.38	-1.19
2	Fagales	-60.50	7	144	100.00	2.50	1000.00	102.92	100.00	107.95	101.62	0.70	0.20	1.16	0.66	0.72	0.00	1.63	0.53	-1.85	-3.76	-0.14	-1.66
2	Garryales	-24.46	2	16	65.00	5.00	1000.00	76.80	65.01	99.10	69.45	0.09	0.00	0.29	0.02	2.02	0.00	4.45	1.02	0.10	-4.31	3.89	1.93
2	Gentianales	-35.90	5	45	70.00	2.50	1000.00	78.21	72.50	91.96	73.83	0.32	0.00	0.69	0.21	1.64	0.00	3.64	1.29	-1.75	-4.64	0.80	-1.77
2	Lamiales	-50.49	24	84	72.50	2.50	1000.00	80.94	75.00	93.46	76.32	0.09	0.00	0.23	0.02	1.85	0.00	4.06	1.19	2.26	-0.11	5.15	1.90
2	Laurales	-63.67	7	63	115.00	2.50	1000.00	121.49	115.00	131.73	118.86	0.21	0.01	0.41	0.21	0.44	0.00	1.25	0.10	0.01	-2.22	2.75	-0.54
2	Magnoliales	-57.21	6	64	115.00	2.50	1000.00	121.57	115.00	134.09	116.73	0.23	0.00	0.45	0.23	0.58	0.00	1.64	0.13	-1.58	-5.44	1.99	-1.34
2	Malpighiales	-67.01	36	157	95.00	2.50	1000.00	105.30	95.00	124.49	98.97	0.03	0.00	0.11	0.01	2.81	0.70	4.73	2.91	3.34	0.39	6.06	3.63
2	Malvales	-45.01	10	55	87.50	2.50	1000.00	96.77	87.50	115.52	91.25	0.14	0.00	0.32	0.03	1.34	0.00	3.13	0.58	-0.50	-3.63	3.17	-0.74
2	Myrtales	-58.30	9	98	95.00	2.50	1000.00	102.28	97.50	112.59	98.88	0.28	0.00	0.59	0.19	1.10	0.00	2.58	0.60	-0.68	-3.64	2.73	-1.13
2	Nymphaeales	-40.05	3	44	102.50	2.50	1000.00	109.00	102.50	123.92	103.80	0.38	0.01	0.76	0.30	0.99	0.00	2.23	0.71	-3.31	-5.58	-0.93	-3.34
2	Oxalidales	-44.64	7	44	87.50	2.50	1000.00	96.12	87.50	111.40	91.65	0.14	0.00	0.32	0.05	1.04	0.00	2.72	0.25	-0.36	-3.96	3.44	-0.42
2	Pandanales	-29.99	5	12	97.50	5.00	1000.00	118.48	97.64	155.47	105.69	0.02	0.00	0.05	0.00	0.70	0.00	1.88	0.18	2.30	-0.67	4.94	3.03
2	Piperales	-28.37	3	17	102.50	5.00	1000.00	123.44	102.50	162.81	109.12	0.02	0.00	0.08	0.00	2.57	0.22	4.75	2.54	0.48	-4.31	4.26	2.31
2	Poales	-44.78	14	71	72.50	2.50	1000.00	79.17	72.50	89.45	76.46	0.19	0.01	0.37	0.21	0.95	0.00	2.67	0.21	0.16	-2.95	3.56	0.51
2	Proteales	-49.68	4	106	115.00	2.50	1000.00	117.53	115.00	122.18	116.43	0.95	0.44	1.45	0.99	0.48	0.00	1.17	0.16	-4.10	-6.44	-2.01	-3.91
2	Ranunculales	-60.01	7	71	115.00	2.50	1000.00	124.53	115.00	144.96	117.24	0.18	0.00	0.47	0.04	1.22	0.00	2.59	0.79	-1.05	-3.66	2.02	-1.46
2	Rosales	-64.33	9	149	100.00	2.50	1000.00	106.89	102.50	116.21	103.70	0.28	0.00	0.62	0.16	1.73	0.38	3.17	1.55	-1.03	-3.21	0.83	-1.04
2	Santalales	-31.73	7	25	72.50	2.50	1000.00	84.94	72.50	107.45	77.13	0.04	0.00	0.15	0.01	2.67	0.00	5.10	2.65	1.47	-2.50	5.06	2.41
2	Sapindales	-61.85	9	139	100.00	2.50	1000.00	104.43	100.00	113.07	101.27	0.31	0.01	0.71	0.19	1.33	0.01	2.70	0.96	-0.97	-3.17	1.74	-1.10
2	Saxifragales	-61.81	15	110	102.50	2.50	1000.00	110.72	105.00	122.77	106.37	0.17	0.00	0.36	0.11	1.05	0.00	2.53	0.60	-0.47	-4.07	3.46	-0.54
2	Solanales	-21.99	5	15	55.00	2.50	1000.00	67.20	55.01	90.25	59.22	0.06	0.00	0.18	0.01	2.36	0.00	5.48	0.79	0.87	-3.00	4.42	1.45
2	Zingiberales	-44.73	8	38	87.50	5.00	1000.00	97.48	87.52	114.41	91.64	0.03	0.00	0.10	0.01	1.45	0.00	3.11	1.08	2.91	-0.19	5.59	3.45
3	Achariaceae	-11.71	155	3	67.50	15.00	999.97	102.61	70.00	173.11	77.15	0.00	0.00	0.00	0.00	1.30	0.00	3.47	0.23	6.28	3.47	8.60	6.64
3	Adoxaceae	-55.17	225	56	75.00	2.50	999.95	85.31	75.68	102.56	79.20	0.00	0.00	0.01	0.00	2.10	0.00	4.09	1.72	6.67	4.05	8.95	7.21
3	Altingiaceae	-54.21	15	55	70.00	5.00	999.94	79.00	70.00	93.88	73.76	0.02	0.00	0.05	0.00	3.11	0.50	5.62	3.03	4.67	2.54	6.53	5.00
3	Amaranthaceae	-8.13	2040	11	10.00	2.50	999.74	15.81	12.50	22.27	14.20	0.00	0.00	0.00	0.00	6.84	0.00	17.79	1.36	9.26	6.72	11.48	9.69
3	Annonaceae	-101.90	2500	59	90.00	2.50	999.71	99.13	92.50	113.17	93.75	0.00	0.00	0.00	0.00	1.15	0.00	2.53	0.74	11.04	9.58	12.31	11.23
3	Apiaceae	-27.14	3575	18	70.00	5.00	999.98	87.53	72.50	116.96	76.20	0.00	0.00	0.00	0.00	3.30	0.30	6.28	2.97	10.10	7.63	11.99	10.58
3	Araceae	-81.04	3750	89	70.00	2.50	999.97	77.30	72.50	86.78	73.77	0.00	0.00	0.00	0.00	0.85	0.00	2.15	0.16	11.01	9.36	12.54	11.30
3	Araliaceae	-76.05	1650	72	70.00	5.00	999.99	77.75	72.50	89.38	73.54	0.00	0.00	0.00	0.00	1.37	0.00	3.08	0.79	9.67	7.49	11.55	10.16
3	Arecaceae	-239.67	2600	186	87.50	2.50	994.99	92.36	87.50	98.93	91.70	0.00	0.00	0.00	0.00	0.30	0.00	0.85	0.05	12.14	11.15	13.11	12.16
3	Asteraceae	-24.05	24700	20	47.50	2.50	1000.00	64.22	50.00	88.49	55.95	0.00	0.00	0.00	0.00	3.38	0.00	6.52	3.22	11.91	9.27	14.04	12.51
3	Atherospermataceae	-22.19	16	9	77.50	2.50	1000.00	98.46	77.50	136.76	83.99	0.01	0.00	0.02	0.00	1.79	0.00	4.08	0.87	2.91	-0.45	5.97	3.69
3	Berberidaceae	-41.77	700	38	45.00	5.00	1000.00	52.61	47.50	62.84	48.90	0.00	0.00	0.00	0.00	1.00	0.00	2.78	0.17	8.92	6.81	10.59	9.23
3	Boraginaceae	-36.07	2535	26	52.50	2.50	961.67	66.37	55.00	88.46	58.71	0.00	0.00	0.00	0.00	1.61	0.00	3.92	0.33	9.73	7.39	11.67	10.11
3	Buxaceae	-18.80	123	13	27.50	5.00	949.93	35.97	30.00	48.04	31.26	0.01	0.00	0.01	0.00	2.18	0.00	5.97	0.38	6.88	4.63	8.77	7.40
3	Calophyllaceae	-32.32	475	16	67.50	2.50	999.98	84.14	70.00	113.07	73.51	0.00	0.00	0.00	0.00	1.77	0.00	4.07	0.97	7.71	5.20	9.89	8.08
3	Campanulaceae	-7.70	2300	3	17.50	5.00	1000.00	37.74	20.00	73.47	24.92	0.00	0.00	0.00	0.00	2.85	0.00	8.15	0.51	9.43	6.86	11.50	10.01
3	Cannabaceae	-52.31	100	55	70.00	2.50	999.87	79.06	70.00	93.71	73.97	0.01	0.00	0.03	0.00	2.28	0.00	4.55	1.78	5.98	3.63	8.09	6.49
3	Celastraceae	-78.08	1350	45	100.00	5.00	999.24	117.37	102.50	150.02	105.28	0.00	0.00	0.00	0.00	2.39	0.51	4.29	2.47	9.88	7.77	11.53	10.26
3	Chrysobalanaceae	-17.10	533	10	20.00	5.00	955.38	30.87	22.50	46.20	25.81	0.00	0.00	0.00	0.00	1.95	0.00	5.51	0.35	8.49	6.20	10.33	8.91
3	Clusiaceae	-41.66	750	17	92.50	2.50	999.92	111.42	95.00	146.23	98.32	0.00	0.00	0.00	0.00	1.30	0.00	2.91	0.77	8.49	6.43	10.34	8.73
3	Cucurbitaceae	-32.92	965	15	52.50	5.00	968.65	69.55	55.00	94.37	61.07	0.00	0.00	0.00	0.00	0.60	0.00	1.74	0.11	9.44	7.63	10.94	9.80
3	Cunoniaceae	-34.90	330	20	82.50	2.50	999.90	100.39	85.00	133.92	88.41	0.00	0.00	0.00	0.00	1.76	0.00	3.86	1.20	6.94	4.13	9.29	7.57
3	Elaeocarpaceae	-45.51	615	23	67.50	2.50	961.29	80.59	70.00	97.96	74.43	0.00	0.00	0.00	0.00	0.57	0.00	1.62	0.09	9.05	7.40	10.46	9.41
3	Elatinaceae	-7.96	35	3	20.00	5.00	999.95	31.17	20.00	50.48	24.18	0.01	0.00	0.02	0.00	4.43	0.00	11.98	0.83	4.76	1.99	7.11	5.37
3	Euphorbiaceae	-92.30	6252	79	70.00	2.50	999.95	76.47	72.50	84.07	73.82	0.00	0.00	0.00	0.00	0.50	0.00	1.34	0.09	12.29	11.24	13.30	12.34
3	Fabaceae	-266.82	19500	442	67.50	2.50	940.13	75.48	70.00	86.32	71.19	0.00	0.00	0.00	0.00	2.84	0.82	4.73	2.86	14.34	13.23	15.35	14.48
3	Fagaceae	-251.32	927	494	97.50	2.50	921.52	109.35	100.00	125.00	101.98	0.00	0.00	0.00	0.00	5.01	3.56	6.44	5.01	11.25	10.00	12.41	11.44
3	Haloragaceae	-37.90	145	32	75.00	2.50	999.91	90.92	77.43	119.57	80.35	0.00	0.00	0.01	0.00	3.88	0.91	6.76	3.64	5.73	3.12	8.30	6.09
3	Hamamelidaceae	-83.71	86	89	92.50	2.50	956.77	103.82	95.00	119.84	98.16	0.01	0.00	0.02	0.00	2.11	0.31	3.81	2.06	6.66	4.61	8.43	7.11
3	Humiriaceae	-14.67	56	7	37.50	5.00	1000.00	51.95	38.85	77.24	42.32	0.01	0.00	0.01	0.00	1.99	0.00	5.28	0.38	5.31	2.51	7.69	5.79
3	Hypericaceae	-33.66	590	33	37.50	5.00	1000.00	45.41	40.00	57.14	41.13	0.00	0.00	0.01	0.00	1.96	0.00	4.99	0.37	7.97	5.13	10.28	8.62
3	Lardizabalaceae	-15.66	40	4	115.00	15.00	999.92	155.79	116.19	231.57	127.41	0.00	0.00	0.00	0.00	1.08	0.00	2.69	0.24	4.40	1.23	7.14	5.21
3	Lauraceae	-285.81	2850	423	110.00	2.50	979.36	123.05	110.00	147.49	114.02	0.00	0.00	0.00	0.00	2.75	1.38	4.12	2.72	12.11	10.75	13.26	12.29
3	Magnoliaceae	-137.02	294	130	105.00	2.50	996.40	113.28	105.00	125.09	108.82	0.00	0.00	0.01	0.00	1.32	0.00	2.52	1.09	8.51	6.94	10.04	8.71

3	Malpighiaceae	-25.54	1315	11	67.50	10.00	999.95	89.63	70.00	128.19	74.35	0.00	0.00	0.00	0.00	1.40	0.00	3.42	0.27	8.66	5.91	10.95	9.17
3	Malvaceae	-104.67	4225	103	67.50	2.50	944.96	74.20	70.00	81.34	71.53	0.00	0.00	0.00	0.00	0.61	0.00	1.61	0.11	11.84	10.61	13.03	12.02
3	Menispermaceae	-113.00	440	82	90.00	5.00	999.73	96.41	90.15	104.67	93.70	0.00	0.00	0.01	0.00	0.68	0.00	1.71	0.13	9.22	8.00	10.42	9.34
3	Menyanthaceae	-22.50	60	18	32.50	5.00	1000.00	40.53	32.50	52.25	36.14	0.01	0.00	0.03	0.01	2.62	0.00	6.65	0.53	5.57	2.74	7.92	6.31
3	Monimiaceae	-15.31	217	4	80.00	15.00	987.66	122.45	82.50	200.77	93.42	0.00	0.00	0.00	0.00	0.78	0.00	2.15	0.14	6.89	4.39	9.08	7.49
3	Myristicaceae	-14.82	520	5	62.50	15.00	999.96	93.44	64.97	151.40	71.89	0.00	0.00	0.00	0.00	1.15	0.00	3.07	0.20	7.73	5.05	9.97	8.30
3	Myrtaceae	-82.80	5950	64	67.50	2.50	953.47	76.90	70.00	87.46	73.63	0.00	0.00	0.00	0.00	0.38	0.00	1.09	0.06	12.08	10.76	13.23	12.29
3	Nyctaginaceae	-12.22	400	5	15.00	10.00	958.85	27.88	17.50	47.72	21.02	0.00	0.00	0.00	0.00	2.42	0.00	6.83	0.41	8.23	5.84	10.04	8.76
3	Ochnaceae	-18.37	550	6	67.50	15.00	1000.00	100.13	70.00	158.64	78.50	0.00	0.00	0.00	0.00	0.84	0.00	2.31	0.16	8.00	5.45	10.13	8.56
3	Orchidaceae	-14.89	28000	4	80.00	22.50	999.97	139.08	82.50	236.18	103.81	0.00	0.00	0.00	0.00	0.70	0.00	1.94	0.12	11.88	9.22	14.08	12.46
3	Passifloraceae	-12.42	980	4	42.50	17.50	999.98	70.79	45.00	123.11	50.94	0.00	0.00	0.00	0.00	1.40	0.00	3.91	0.26	8.50	5.83	10.71	9.08
3	Phyllanthaceae	-33.89	2050	19	67.50	5.00	1000.00	83.94	70.00	113.11	72.52	0.00	0.00	0.00	0.00	2.06	0.00	4.46	1.46	9.47	7.22	11.45	9.83
3	Piperaceae	-13.32	3700	4	70.00	5.00	999.90	109.74	72.50	183.23	81.58	0.00	0.00	0.00	0.00	1.22	0.00	3.23	0.24	9.86	7.22	12.00	10.45
3	Pittosporaceae	-11.07	245	4	40.00	5.00	1000.00	59.49	42.45	96.96	45.93	0.00	0.00	0.00	0.00	2.52	0.00	6.51	0.54	6.76	4.19	9.01	7.23
3	Poaceae	-51.21	12000	39	67.50	5.00	1000.00	81.71	69.99	104.49	73.32	0.00	0.00	0.00	0.00	2.07	0.00	3.99	1.82	11.21	8.72	13.27	11.68
3	Polygonaceae	-29.09	1200	33	32.50	2.50	1000.00	40.51	35.00	52.80	36.52	0.00	0.00	0.00	0.00	3.71	0.00	8.01	2.66	8.66	6.13	10.88	9.27
3	Primulaceae	-34.62	2790	23	67.50	5.00	1000.00	83.84	70.00	112.24	72.96	0.00	0.00	0.00	0.00	2.36	0.00	4.73	1.90	9.68	7.20	11.64	10.18
3	Proteaceae	-60.89	1660	50	62.50	2.50	999.96	69.36	64.99	78.90	66.02	0.00	0.00	0.00	0.00	1.08	0.00	2.76	0.21	10.38	8.98	11.60	10.58
3	Ranunculaceae	-57.41	2346	45	62.50	5.00	999.91	75.85	65.00	97.94	66.80	0.00	0.00	0.00	0.00	4.45	1.40	7.50	4.29	9.97	7.90	11.81	10.31
3	Rhamnaceae	-123.27	950	159	75.00	2.50	999.20	82.39	75.00	93.30	78.52	0.00	0.00	0.01	0.00	2.42	0.30	4.42	2.46	10.33	8.86	11.60	10.59
3	Rhizophoraceae	-25.22	147	12	52.50	2.50	962.75	67.45	55.00	91.82	59.10	0.00	0.00	0.01	0.00	1.59	0.00	4.06	0.29	6.39	3.99	8.51	6.75
3	Salicaceae	-150.93	1220	284	67.50	2.50	942.19	74.46	70.00	82.89	71.03	0.00	0.00	0.01	0.00	2.34	0.34	4.31	2.34	10.98	9.61	12.15	11.17
3	Saururaceae	-17.79	6	9	45.00	5.00	1000.00	57.06	45.00	77.79	49.42	0.02	0.00	0.07	0.01	2.00	0.00	5.14	0.39	2.79	-0.16	5.37	3.31
3	Schisandraceae	-29.32	85	18	45.00	5.00	1000.00	54.78	47.19	70.22	48.86	0.01	0.00	0.02	0.00	1.31	0.00	3.57	0.25	6.33	4.14	8.28	6.72
3	Torricelliaceae	-10.84	10	3	57.50	20.00	999.99	86.52	57.61	143.32	66.10	0.00	0.00	0.01	0.00	1.25	0.00	3.43	0.23	3.39	0.54	5.82	3.96
3	Trochodendraceae	-20.55	2	8	65.00	10.00	972.12	85.04	67.50	116.80	73.99	0.02	0.00	0.07	0.01	0.86	0.00	2.38	0.15	1.81	-1.12	4.33	2.56
3	Typhaceae	-75.27	51	93	62.50	5.00	998.32	73.54	62.50	92.09	66.44	0.00	0.00	0.01	0.00	5.40	2.48	8.49	5.45	7.19	5.86	8.42	7.34
3	Urticaceae	-39.10	2625	33	52.50	5.00	1000.00	63.06	55.00	79.95	56.48	0.00	0.00	0.00	0.00	2.34	0.00	5.02	1.55	9.61	7.24	11.65	10.03
3	Violaceae	-26.58	980	15	75.00	5.00	999.95	94.25	77.50	126.98	81.15	0.00	0.00	0.00	0.00	3.61	0.43	6.85	3.40	8.45	6.35	10.43	8.67
3	Vitaceae	-123.15	910	160	67.50	2.50	999.57	71.99	67.50	76.64	70.53	0.01	0.00	0.01	0.01	0.53	0.00	1.42	0.10	10.27	9.06	11.40	10.42
3	Winteraceae	-11.77	65	3	80.00	15.00	999.97	119.53	82.32	198.37	90.36	0.00	0.00	0.00	0.00	1.05	0.00	2.82	0.20	5.25	2.23	7.70	5.88

Supplementary Table 2. Calibration densities derived from BBB results.
The Skew-t distributions were fitted onto the posterior age densities inferred by BBB using the "sn" Package. To specify the uniform densities with soft and hard bounds, we used the 2.5% and 97.5% quantiles to specify the minimum and maximum ages as bounds, respectively.

No	Calib	node	Prior_uniform_hardbounds	Prior_uniform_softbounds	Prior_skew_T
1	Tracheophyta	645	B(4.22,4.51,0.01,0.05)	B(4.22,4.51,0.01,0.05)	B(4.22,4.51,0.01,0.05)
2	Euphyllophytes	646	B(3.86,4.51,0.01,0.05)	B(3.86,4.51,0.01,0.05)	B(3.86,4.51,0.01,0.05)
3	Spermatophytes	647	B(3.08,3.66,0.01,0.05)	B(3.08,3.66,0.01,0.05)	B(3.08,3.66,0.01,0.05)
4	angiosperm	648	B(1.33,1.53,1e-300,1e-300)	B(1.33,1.53,0.025,0.025)	ST(1.337,0.08,6.638,15.561)
5	Trochodendraceae	657	B(0.68,1.3,1e-300,1e-300)	B(0.68,1.3,0.025,0.025)	removed - mcmc_clash
6	Malpighiales	668	B(0.96,1.35,1e-300,1e-300)	B(0.96,1.35,0.025,0.025)	ST(0.967,0.07,6.124,2.337)
7	Euphorbiaceae	674	B(0.73,0.86,1e-300,1e-300)	B(0.73,0.86,0.025,0.025)	ST(0.726,0.039,16.22,3.495)
8	Malpighiaceae	697	B(0.71,1.45,1e-300,1e-300)	B(0.71,1.45,0.025,0.025)	ST(0.7,0.178,108.856,2.934)
9	Elatinaceae	702	B(0.22,0.6,1e-300,1e-300)	B(0.22,0.6,0.025,0.025)	ST(0.224,0.071,8.819,2.227)
10	Ochnaceae	704	B(0.71,1.79,1e-300,1e-300)	B(0.71,1.79,0.025,0.025)	ST(0.702,0.286,89.896,3.484)
11	Phyllanthaceae	714	B(0.7,1.26,1e-300,1e-300)	B(0.7,1.26,0.025,0.025)	ST(0.7,0.12,90.408,2.551)
12	Chrysobalanaceae	734	B(0.23,0.52,1e-300,1e-300)	B(0.23,0.52,0.025,0.025)	ST(0.225,0.083,608.917,3.882)
13	Rhizophoraceae	741	B(0.55,1.02,1e-300,1e-300)	B(0.55,1.02,0.025,0.025)	ST(0.55,0.116,267.652,3.149)
14	Achariaceae	746	B(0.71,2.01,1e-300,1e-300)	B(0.71,2.01,0.025,0.025)	ST(0.701,0.29,71.339,2.693)
15	Salicaceae	748	B(0.7,0.86,1e-300,1e-300)	B(0.7,0.86,0.025,0.025)	ST(0.7,0.043,1375.69,3.617)
16	Violaceae	762	B(0.78,1.38,1e-300,1e-300)	B(0.78,1.38,0.025,0.025)	ST(0.775,0.158,78.71,3.313)
17	Passifloraceae	766	B(0.46,1.45,1e-300,1e-300)	B(0.46,1.45,0.025,0.025)	ST(0.451,0.231,147.504,2.863)
18	Calophyllaceae	777	B(0.7,1.24,1e-300,1e-300)	B(0.7,1.24,0.025,0.025)	ST(0.701,0.127,47.076,2.828)
19	Hypericaceae	779	B(0.4,0.61,1e-300,1e-300)	B(0.4,0.61,0.025,0.025)	ST(0.401,0.048,17.992,2.648)
20	Clusiaceae	785	B(0.96,1.62,1e-300,1e-300)	B(0.96,1.62,0.025,0.025)	ST(0.95,0.143,81.354,2.567)
21	Humiriaceae	791	B(0.4,0.87,1e-300,1e-300)	B(0.4,0.87,0.025,0.025)	ST(0.401,0.104,18.567,2.64)
22	Oxalidales	796	B(0.88,1.18,1e-300,1e-300)	B(0.88,1.18,0.025,0.025)	ST(0.875,0.086,509.428,4.115)
23	Cunoniaceae	799	B(0.85,1.47,1e-300,1e-300)	B(0.85,1.47,0.025,0.025)	ST(0.85,0.133,42.784,2.539)
24	Elaeocarpaceae	800	B(0.71,1.05,1e-300,1e-300)	B(0.71,1.05,0.025,0.025)	ST(0.701,0.11,139.778,5.264)
25	Celastraceae	809	B(1.03,1.64,1e-300,1e-300)	B(1.03,1.64,0.025,0.025)	ST(1.025,0.125,93.539,2.374)
26	Fagales	825	B(1,1.1,1e-300,1e-300)	B(1,1.1,0.025,0.025)	ST(1,0.029,536.345,3.832)
27	Fagaceae	826	B(1,1.3,1e-300,1e-300)	B(1,1.3,0.025,0.025)	ST(1,0.105,120.132,7.317)
28	Cucurbitales	832	B(0.54,0.92,1e-300,1e-300)	B(0.54,0.92,0.025,0.025)	ST(0.544,0.082,7.122,2.702)
29	Cucurbitaceae	834	B(0.56,1.05,1e-300,1e-300)	B(0.56,1.05,0.025,0.025)	ST(0.552,0.146,67.672,4.512)
30	Rosales	837	B(1.03,1.22,1e-300,1e-300)	B(1.03,1.22,0.025,0.025)	ST(1.025,0.034,462.919,2.099)
31	Urticaceae	841	B(0.55,0.87,1e-300,1e-300)	B(0.55,0.87,0.025,0.025)	ST(0.55,0.07,43.855,2.598)
32	Cannabaceae	843	B(0.72,1,1e-300,1e-300)	B(0.72,1,0.025,0.025)	ST(0.725,0.054,7.318,2.278)
33	Rhamnaceae	844	B(0.77,0.99,1e-300,1e-300)	B(0.77,0.99,0.025,0.025)	ST(0.775,0.039,5.522,2.1)

34	Fabales	847	B(1,1.42,1e-300,1e-300)	B(1,1.42,0.025,0.025)	ST(1,0.096,372.385,2.742)
35	Fabaceae	848	B(0.7,0.94,1e-300,1e-300)	B(0.7,0.94,0.025,0.025)	ST(0.7,0.049,154.092,2.512)
36	Brassicales	862	B(0.64,1.07,1e-300,1e-300)	B(0.64,1.07,0.025,0.025)	ST(0.646,0.095,8.124,2.708)
37	Malvales	869	B(0.88,1.25,1e-300,1e-300)	B(0.88,1.25,0.025,0.025)	ST(0.875,0.084,312.101,2.83)
38	Malvaceae	872	B(0.7,0.84,1e-300,1e-300)	B(0.7,0.84,0.025,0.025)	ST(0.7,0.046,649.782,6.267)
39	Sapindales	875	B(1,1.18,1e-300,1e-300)	B(1,1.18,0.025,0.025)	ST(1,0.04,296.338,2.882)
40	Crossosomatales	881	B(0.61,1.06,1e-300,1e-300)	B(0.61,1.06,0.025,0.025)	ST(0.617,0.103,8.495,2.902)
41	Myrtales	887	B(0.98,1.18,1e-300,1e-300)	B(0.98,1.18,0.025,0.025)	ST(0.975,0.038,371.243,2.234)
42	Myrtaceae	891	B(0.7,0.91,1e-300,1e-300)	B(0.7,0.91,0.025,0.025)	ST(0.7,0.077,1175.373,8.242)
43	Vitaceae	897	B(0.68,0.78,1e-300,1e-300)	B(0.68,0.78,0.025,0.025)	ST(0.702,0.021,1.485,3.802)
44	Saxifragales	898	B(1.05,1.29,1e-300,1e-300)	B(1.05,1.29,0.025,0.025)	ST(1.05,0.05,436.515,2.521)
45	Haloragaceae	906	B(0.78,1.28,1e-300,1e-300)	B(0.78,1.28,0.025,0.025)	ST(0.775,0.126,21.366,3.141)
46	Altingiaceae	913	B(0.71,1,1e-300,1e-300)	B(0.71,1,0.025,0.025)	removed - mcmc_clash
47	Hamamelidaceae	914	B(0.95,1.26,1e-300,1e-300)	B(0.95,1.26,0.025,0.025)	ST(0.95,0.087,1061.88,3.907)
48	Dipsacales	933	B(0.95,1.38,1e-300,1e-300)	B(0.95,1.38,0.025,0.025)	ST(0.95,0.059,454.689,1.717)
49	Adoxaceae	958	B(0.78,1.1,1e-300,1e-300)	B(0.78,1.1,0.025,0.025)	ST(0.775,0.065,12.265,2.333)
50	Apiales	964	B(0.96,1.59,1e-300,1e-300)	B(0.96,1.59,0.025,0.025)	ST(0.96,0.146,14.481,2.97)
51	Torricelliaceae	965	B(0.61,1.69,1e-300,1e-300)	B(0.61,1.69,0.025,0.025)	ST(0.6,0.229,26.089,2.583)
52	Araliaceae	968	B(0.73,0.94,1e-300,1e-300)	B(0.73,0.94,0.025,0.025)	ST(0.725,0.045,19.475,2.399)
53	Apiaceae	970	B(0.73,1.28,1e-300,1e-300)	B(0.73,1.28,0.025,0.025)	ST(0.725,0.142,170.401,3.245)
54	Pittosporaceae	990	B(0.43,1.11,1e-300,1e-300)	B(0.43,1.11,0.025,0.025)	ST(0.426,0.144,39.518,2.457)
55	Asterales	1002	B(0.43,0.7,1e-300,1e-300)	B(0.43,0.7,0.025,0.025)	removed - mcmc_clash
56	Asteraceae	1008	B(0.51,0.96,1e-300,1e-300)	B(0.51,0.96,0.025,0.025)	ST(0.501,0.152,196.221,5.942)
57	Menyanthaceae	1021	B(0.35,0.57,1e-300,1e-300)	B(0.35,0.57,0.025,0.025)	ST(0.35,0.046,6.373,2.287)
58	Campanulaceae	1033	B(0.21,0.92,1e-300,1e-300)	B(0.21,0.92,0.025,0.025)	ST(0.2,0.155,337.75,2.73)
59	Lamiales	1054	B(0.75,0.99,1e-300,1e-300)	B(0.75,0.99,0.025,0.025)	ST(0.75,0.051,136.418,2.597)
60	Solanales	1075	B(0.56,1.02,1e-300,1e-300)	B(0.56,1.02,0.025,0.025)	ST(0.55,0.11,279.88,3.006)
61	Gentianales	1083	B(0.73,1,1e-300,1e-300)	B(0.73,1,0.025,0.025)	ST(0.725,0.04,317.366,1.821)
62	Boraginaceae	1090	B(0.55,0.97,1e-300,1e-300)	B(0.55,0.97,0.025,0.025)	ST(0.55,0.109,710.583,3.412)
63	Garryales	1093	B(0.65,1.08,1e-300,1e-300)	B(0.65,1.08,0.025,0.025)	ST(0.651,0.115,173.807,3.624)
64	Ericales	1094	B(1.13,1.58,1e-300,1e-300)	B(1.13,1.58,0.025,0.025)	ST(1.14,0.097,8.567,2.848)
65	Primulaceae	1106	B(0.7,1.23,1e-300,1e-300)	B(0.7,1.23,0.025,0.025)	ST(0.7,0.126,102.731,2.93)
66	Cornales	1115	B(0.9,1.2,1e-300,1e-300)	B(0.9,1.2,0.025,0.025)	ST(0.902,0.078,28.513,3.729)
67	Caryophyllales	1120	B(0.89,1.47,1e-300,1e-300)	B(0.89,1.47,0.025,0.025)	ST(0.895,0.139,11.741,3.001)

68	Nyctaginaceae	1141	B(0.18,0.56,1e-300,1e-300)	B(0.18,0.56,0.025,0.025)	ST(0.175,0.098,353.5,3.326)
69	Amaranthaceae	1147	B(0.13,0.25,1e-300,1e-300)	B(0.13,0.25,0.025,0.025)	ST(0.126,0.03,21.612,3)
70	Polygonaceae	1153	B(0.35,0.57,1e-300,1e-300)	B(0.35,0.57,0.025,0.025)	ST(0.351,0.046,18.638,2.307)
71	Santalales	1163	B(0.73,1.15,1e-300,1e-300)	B(0.73,1.15,0.025,0.025)	ST(0.736,0.108,10.859,3.449)
72	Buxaceae	1171	B(0.3,0.53,1e-300,1e-300)	B(0.3,0.53,0.025,0.025)	ST(0.3,0.053,385.069,2.797)
73	Proteales	1174	B(1.15,1.24,1e-300,1e-300)	B(1.15,1.24,0.025,0.025)	ST(1.15,0.025,325.746,3.553)
74	Proteaceae	1176	B(0.65,0.82,1e-300,1e-300)	B(0.65,0.82,0.025,0.025)	ST(0.651,0.038,12.919,2.559)
75	Ranunculales	1179	B(1.15,1.56,1e-300,1e-300)	B(1.15,1.56,0.025,0.025)	ST(1.15,0.076,574.125,2.308)
76	Menispermaceae	1182	B(0.92,1.07,1e-300,1e-300)	B(0.92,1.07,0.025,0.025)	ST(0.925,0.036,4.382,2.831)
77	Ranunculaceae	1183	B(0.65,1.05,1e-300,1e-300)	B(0.65,1.05,0.025,0.025)	ST(0.65,0.1,76.684,2.991)
78	Berberidaceae	1186	B(0.48,0.66,1e-300,1e-300)	B(0.48,0.66,0.025,0.025)	ST(0.475,0.048,22.374,3.075)
79	Lardizabalaceae	1188	B(1.19,2.53,1e-300,1e-300)	B(1.19,2.53,0.025,0.025)	ST(1.177,0.391,41.935,4.188)
80	Arecaceae	1198	B(0.88,1.01,1e-300,1e-300)	B(0.88,1.01,0.025,0.025)	ST(0.889,0.04,3.358,6.699)
81	Poales	1200	B(0.73,0.93,1e-300,1e-300)	B(0.73,0.93,0.025,0.025)	ST(0.725,0.073,152.76,7.459)
82	Poaceae	1201	B(0.7,1.14,1e-300,1e-300)	B(0.7,1.14,0.025,0.025)	ST(0.7,0.112,243.296,3.272)
83	Typhaceae	1205	B(0.65,1,1e-300,1e-300)	B(0.65,1,0.025,0.025)	ST(0.649,0.073,8.212,2.484)
84	Zingiberales	1208	B(0.89,1.23,1e-300,1e-300)	B(0.89,1.23,0.025,0.025)	ST(0.894,0.076,6.699,2.768)
85	Asparagales	1212	B(0.7,1.2,1e-300,1e-300)	B(0.7,1.2,0.025,0.025)	ST(0.7,0.125,480.96,3.111)
86	Orchidaceae	1221	B(0.86,2.61,1e-300,1e-300)	B(0.86,2.61,0.025,0.025)	ST(0.847,0.631,31.109,9.444)
87	Pandanales	1225	B(1,1.69,1e-300,1e-300)	B(1,1.69,0.025,0.025)	ST(0.999,0.178,15.179,3.416)
88	Alismatales	1226	B(0.83,1.1,1e-300,1e-300)	B(0.83,1.1,0.025,0.025)	ST(0.825,0.07,274.653,3.588)
89	Araceae	1232	B(0.73,0.9,1e-300,1e-300)	B(0.73,0.9,0.025,0.025)	ST(0.725,0.044,34.814,2.98)
90	Laurales	1237	B(1.15,1.35,1e-300,1e-300)	B(1.15,1.35,0.025,0.025)	ST(1.15,0.073,528.2,8.591)
91	Lauraceae	1239	B(1.12,1.59,1e-300,1e-300)	B(1.12,1.59,0.025,0.025)	ST(1.121,0.087,8.551,2.26)
92	Monimiaceae	1240	B(0.85,2.27,1e-300,1e-300)	B(0.85,2.27,0.025,0.025)	ST(0.833,0.386,47.392,3.721)
93	Atherospermataceae	1245	B(0.8,1.53,1e-300,1e-300)	B(0.8,1.53,0.025,0.025)	ST(0.798,0.169,16.316,2.862)
94	Magnoliales	1248	B(1.15,1.4,1e-300,1e-300)	B(1.15,1.4,0.025,0.025)	ST(1.15,0.062,599.03,3.23)
95	Magnoliaceae	1249	B(1.07,1.3,1e-300,1e-300)	B(1.07,1.3,0.025,0.025)	ST(1.074,0.054,5.97,2.848)
96	Annonaceae	1252	B(0.93,1.18,1e-300,1e-300)	B(0.93,1.18,0.025,0.025)	ST(0.925,0.056,22.654,2.442)
97	Myristicaceae	1254	B(0.66,1.72,1e-300,1e-300)	B(0.66,1.72,0.025,0.025)	ST(0.651,0.26,86.568,3.043)
98	Piperales	1257	B(1.04,1.78,1e-300,1e-300)	B(1.04,1.78,0.025,0.025)	ST(1.035,0.19,20.09,3.331)
99	Piperaceae	1259	B(0.74,2.11,1e-300,1e-300)	B(0.74,2.11,0.025,0.025)	ST(0.726,0.36,259.89,3.499)
100	Saururaceae	1260	B(0.46,0.87,1e-300,1e-300)	B(0.46,0.87,0.025,0.025)	ST(0.47,0.091,7.974,2.862)
101	Canellales	1264	B(1.01,2.32,1e-300,1e-300)	B(1.01,2.32,0.025,0.025)	ST(0.999,0.366,25.883,3.989)

102	Winteraceae	1265	B(0.84,2.25,1e-300,1e-300)	B(0.84,2.25,0.025,0.025)	ST(0.826,0.344,57.645,3.035)
103	Schisandraceae	1273	B(0.48,0.75,1e-300,1e-300)	B(0.48,0.75,0.025,0.025)	ST(0.476,0.065,17.102,2.822)
104	Nymphaeales	1275	B(1.03,1.31,1e-300,1e-300)	B(1.03,1.31,0.025,0.025)	ST(1.025,0.049,451.84,2.008)
105	Acrogymnosperms	1279	B(3.08,3.66,0.01,0.05)	B(3.08,3.66,0.01,0.05)	B(3.08,3.66,0.01,0.05)
106	Conifers	1280	B(1.47,3.12,0.01,0.05)	B(1.47,3.12,0.01,0.05)	B(1.47,3.12,0.01,0.05)
107	Gnetales	1282	B(1.2,3.12,0.01,0.05)	B(1.2,3.12,0.01,0.05)	B(1.2,3.12,0.01,0.05)
108	Ginkgo-Cycas	1284	B(2.65,3.66,0.01,0.05)	B(2.65,3.66,0.01,0.05)	B(2.65,3.66,0.01,0.05)
109	Ferns	1286	B(3.85,4.51,0.01,0.05)	B(3.85,4.51,0.01,0.05)	B(3.85,4.51,0.01,0.05)
110	Lycophytes	1287	B(3.92,4.51,0.01,0.05)	B(3.92,4.51,0.01,0.05)	B(3.92,4.51,0.01,0.05)

NOTE: time unit is 100Ma

Supplementary Table 3. Taxonomical classification of the 644 species included in molecular clock-dating analysis.
The species included in this list follow Barba-Montoya et al., 2018. Please see the main text for more details.

No	Species	Family	Order	Clade_1	Clade_2
1	Acalypha	Euphorbiaceae	Malpighiales	Eudicots	Rosids
2	Spathiostemon	Euphorbiaceae	Malpighiales	Eudicots	Rosids
3	Ricinus	Euphorbiaceae	Malpighiales	Eudicots	Rosids
4	Lasiocroton	Euphorbiaceae	Malpighiales	Eudicots	Rosids
5	Dalechampia	Euphorbiaceae	Malpighiales	Eudicots	Rosids
6	Conceveiba	Euphorbiaceae	Malpighiales	Eudicots	Rosids
7	Homalanthus	Euphorbiaceae	Malpighiales	Eudicots	Rosids
8	Hura	Euphorbiaceae	Malpighiales	Eudicots	Rosids
9	Euphorbia	Euphorbiaceae	Malpighiales	Eudicots	Rosids
10	Pimelodendron	Euphorbiaceae	Malpighiales	Eudicots	Rosids
11	Trigonostemon	Euphorbiaceae	Malpighiales	Eudicots	Rosids
12	Codiaeum	Euphorbiaceae	Malpighiales	Eudicots	Rosids
13	Croton	Euphorbiaceae	Malpighiales	Eudicots	Rosids
14	Hevea	Euphorbiaceae	Malpighiales	Eudicots	Rosids
15	Manihot	Euphorbiaceae	Malpighiales	Eudicots	Rosids
16	Moultonianthus	Euphorbiaceae	Malpighiales	Eudicots	Rosids
17	Suregada	Euphorbiaceae	Malpighiales	Eudicots	Rosids
18	Omphalea	Euphorbiaceae	Malpighiales	Eudicots	Rosids
19	Tetrorchidium	Euphorbiaceae	Malpighiales	Eudicots	Rosids
20	Endospermum	Euphorbiaceae	Malpighiales	Eudicots	Rosids
21	Neoscortechinia	Euphorbiaceae	Malpighiales	Eudicots	Rosids
22	Clutia	Peraceae	Malpighiales	Eudicots	Rosids
23	Pera	Peraceae	Malpighiales	Eudicots	Rosids
24	Pogonophora	Peraceae	Malpighiales	Eudicots	Rosids
25	Dicella	Malpighiaceae	Malpighiales	Eudicots	Rosids
26	Malpighia	Malpighiaceae	Malpighiales	Eudicots	Rosids
27	Thryallis	Malpighiaceae	Malpighiales	Eudicots	Rosids
28	Acridocarpus	Malpighiaceae	Malpighiales	Eudicots	Rosids
29	Byrsonima	Malpighiaceae	Malpighiales	Eudicots	Rosids
30	Elatine	Elatinaceae	Malpighiales	Eudicots	Rosids
31	Bergia	Elatinaceae	Malpighiales	Eudicots	Rosids
32	Caryocar	Caryocaraceae	Malpighiales	Eudicots	Rosids
33	Austrobuxus	Picrodendraceae	Malpighiales	Eudicots	Rosids
34	Dissiliaria	Picrodendraceae	Malpighiales	Eudicots	Rosids
35	Micrantheum	Picrodendraceae	Malpighiales	Eudicots	Rosids
36	Petalostigma	Picrodendraceae	Malpighiales	Eudicots	Rosids
37	Tetracoccus	Picrodendraceae	Malpighiales	Eudicots	Rosids
38	Androstachys	Picrodendraceae	Malpighiales	Eudicots	Rosids
39	Podocalyx	Picrodendraceae	Malpighiales	Eudicots	Rosids
40	Lachnostylis	Phyllanthaceae	Malpighiales	Eudicots	Rosids
41	Croizatia	Phyllanthaceae	Malpighiales	Eudicots	Rosids
42	Heywoodia	Phyllanthaceae	Malpighiales	Eudicots	Rosids
43	Phyllanthus	Phyllanthaceae	Malpighiales	Eudicots	Rosids
44	Bischofia	Phyllanthaceae	Malpighiales	Eudicots	Rosids
45	Aporusa	Phyllanthaceae	Malpighiales	Eudicots	Rosids
46	Hugonia	Linaceae	Malpighiales	Eudicots	Rosids

47	Durandea	Linaceae	Malpighiales	Eudicots	Rosids
48	Linum	Linaceae	Malpighiales	Eudicots	Rosids
49	Reinwardtia	Linaceae	Malpighiales	Eudicots	Rosids
50	Centroplacus	Centroplacaceae	Malpighiales	Eudicots	Rosids
51	Bhesa	Centroplacaceae	Malpighiales	Eudicots	Rosids
52	Cespedesia	Ochnaceae	Malpighiales	Eudicots	Rosids
53	Sauvagesia	Ochnaceae	Malpighiales	Eudicots	Rosids
54	Luxemburgia	Ochnaceae	Malpighiales	Eudicots	Rosids
55	Ochna	Ochnaceae	Malpighiales	Eudicots	Rosids
56	Touroulia	Ochnaceae	Malpighiales	Eudicots	Rosids
57	Quiina	Ochnaceae	Malpighiales	Eudicots	Rosids
58	Medusagyne	Ochnaceae	Malpighiales	Eudicots	Rosids
59	Putranjiva	Putranjivaceae	Malpighiales	Eudicots	Rosids
60	Drypetes	Putranjivaceae	Malpighiales	Eudicots	Rosids
61	Lophopyxis	Lophopyxidaceae	Malpighiales	Eudicots	Rosids
62	Hirtella	Chrysobalanaceae	Malpighiales	Eudicots	Rosids
63	Licania	Chrysobalanaceae	Malpighiales	Eudicots	Rosids
64	Atuna	Chrysobalanaceae	Malpighiales	Eudicots	Rosids
65	Chrysobalanus	Chrysobalanaceae	Malpighiales	Eudicots	Rosids
66	Euphronia	Euphroniaceae	Malpighiales	Eudicots	Rosids
67	Tapura	Dichapetalaceae	Malpighiales	Eudicots	Rosids
68	Dichapetalum	Dichapetalaceae	Malpighiales	Eudicots	Rosids
69	Trigonia	Trigoniaceae	Malpighiales	Eudicots	Rosids
70	Balanops	Balanopaceae	Malpighiales	Eudicots	Rosids
71	Carallia	Rhizophoraceae	Malpighiales	Eudicots	Rosids
72	Bruguiera	Rhizophoraceae	Malpighiales	Eudicots	Rosids
73	Rhizophora	Rhizophoraceae	Malpighiales	Eudicots	Rosids
74	Paradrypetes	Rhizophoraceae	Malpighiales	Eudicots	Rosids
75	Erythroxylum	Erythroxylaceae	Malpighiales	Eudicots	Rosids
76	Ctenolophon	Ctenolophonaceae	Malpighiales	Eudicots	Rosids
77	Populus	Salicaceae	Malpighiales	Eudicots	Rosids
78	Salix	Salicaceae	Malpighiales	Eudicots	Rosids
79	Idesia	Salicaceae	Malpighiales	Eudicots	Rosids
80	Poliothyrsis	Salicaceae	Malpighiales	Eudicots	Rosids
81	Flacourtia	Salicaceae	Malpighiales	Eudicots	Rosids
82	Dovyalis	Salicaceae	Malpighiales	Eudicots	Rosids
83	Prockia	Salicaceae	Malpighiales	Eudicots	Rosids
84	Abatia	Salicaceae	Malpighiales	Eudicots	Rosids
85	Scyphostegia	Salicaceae	Malpighiales	Eudicots	Rosids
86	Lunania	Salicaceae	Malpighiales	Eudicots	Rosids
87	Casearia	Salicaceae	Malpighiales	Eudicots	Rosids
88	Lacistema	Lacistemataceae	Malpighiales	Eudicots	Rosids
89	Lozania	Lacistemataceae	Malpighiales	Eudicots	Rosids
90	Hymenanthera	Violaceae	Malpighiales	Eudicots	Rosids
91	Viola	Violaceae	Malpighiales	Eudicots	Rosids
92	Hybanthus	Violaceae	Malpighiales	Eudicots	Rosids
93	Leonia	Violaceae	Malpighiales	Eudicots	Rosids

94	Rinorea	Violaceae	Malpighiales	Eudicots	Rosids
95	Passiflora	Passifloraceae	Malpighiales	Eudicots	Rosids
96	Paropsia	Passifloraceae	Malpighiales	Eudicots	Rosids
97	Turnera	Passifloraceae	Malpighiales	Eudicots	Rosids
98	Malesherbia	Passifloraceae	Malpighiales	Eudicots	Rosids
99	Goupia	Goupiaceae	Malpighiales	Eudicots	Rosids
100	Caloncoba	Achariaceae	Malpighiales	Eudicots	Rosids
101	Carpotroche	Achariaceae	Malpighiales	Eudicots	Rosids
102	Erythrospermum	Achariaceae	Malpighiales	Eudicots	Rosids
103	Hydnocarpus	Achariaceae	Malpighiales	Eudicots	Rosids
104	Acharia	Achariaceae	Malpighiales	Eudicots	Rosids
105	Kiggelaria	Achariaceae	Malpighiales	Eudicots	Rosids
106	Pangium	Achariaceae	Malpighiales	Eudicots	Rosids
107	Triadenum	Hypericaceae	Malpighiales	Eudicots	Rosids
108	Hypericum	Hypericaceae	Malpighiales	Eudicots	Rosids
109	Cratoxylum	Hypericaceae	Malpighiales	Eudicots	Rosids
110	Vismia	Hypericaceae	Malpighiales	Eudicots	Rosids
111	Podostemum	Podostemaceae	Malpighiales	Eudicots	Rosids
112	Marathrum	Podostemaceae	Malpighiales	Eudicots	Rosids
113	Calophyllum	Calophyllaceae	Malpighiales	Eudicots	Rosids
114	Mesua	Calophyllaceae	Malpighiales	Eudicots	Rosids
115	Mammea	Calophyllaceae	Malpighiales	Eudicots	Rosids
116	Pentaphalangium	Clusiaceae	Malpighiales	Eudicots	Rosids
117	Garcinia	Clusiaceae	Malpighiales	Eudicots	Rosids
118	Clusia	Clusiaceae	Malpighiales	Eudicots	Rosids
119	Bonnetia	Bonnetiaceae	Malpighiales	Eudicots	Rosids
120	Archytaea	Bonnetiaceae	Malpighiales	Eudicots	Rosids
121	Klainedoxa	Irvingiaceae	Malpighiales	Eudicots	Rosids
122	Irvingia	Irvingiaceae	Malpighiales	Eudicots	Rosids
123	Ochthocosmus	Ixonanthaceae	Malpighiales	Eudicots	Rosids
124	Panda	Pandaceae	Malpighiales	Eudicots	Rosids
125	Galearia	Pandaceae	Malpighiales	Eudicots	Rosids
126	Microdesmis	Pandaceae	Malpighiales	Eudicots	Rosids
127	Humiria	Humiriaceae	Malpighiales	Eudicots	Rosids
128	Vantanea	Humiriaceae	Malpighiales	Eudicots	Rosids
129	Sacoglottis	Humiriaceae	Malpighiales	Eudicots	Rosids
130	Crinodendron	Elaeocarpaceae	Oxalidales	Eudicots	Rosids
131	Elaeocarpus	Elaeocarpaceae	Oxalidales	Eudicots	Rosids
132	Sloanea	Elaeocarpaceae	Oxalidales	Eudicots	Rosids
133	Cephalotus	Cephalotaceae	Oxalidales	Eudicots	Rosids
134	Eucryphia	Cunoniaceae	Oxalidales	Eudicots	Rosids
135	Geissois	Cunoniaceae	Oxalidales	Eudicots	Rosids
136	Brunellia	Brunelliaceae	Oxalidales	Eudicots	Rosids
137	Dapania	Oxalidaceae	Oxalidales	Eudicots	Rosids
138	Averrhoa	Oxalidaceae	Oxalidales	Eudicots	Rosids
139	Oxalis	Oxalidaceae	Oxalidales	Eudicots	Rosids
140	Connarus	Connaraceae	Oxalidales	Eudicots	Rosids

141 Rourea	Connaraceae	Oxalidales	Eudicots	Rosids
142 Hua	Huaceae	Oxalidales	Eudicots	Rosids
143 Afrostyrax	Huaceae	Oxalidales	Eudicots	Rosids
144 Paxistima	Celastraceae	Celastrales	Eudicots	Rosids
145 Euonymus	Celastraceae	Celastrales	Eudicots	Rosids
146 Celastrus	Celastraceae	Celastrales	Eudicots	Rosids
147 Tripterygium	Celastraceae	Celastrales	Eudicots	Rosids
148 Maytenus	Celastraceae	Celastrales	Eudicots	Rosids
149 Elaeodendron	Celastraceae	Celastrales	Eudicots	Rosids
150 Brexia	Celastraceae	Celastrales	Eudicots	Rosids
151 Plagiopteron	Celastraceae	Celastrales	Eudicots	Rosids
152 Stackhousia	Celastraceae	Celastrales	Eudicots	Rosids
153 Denhamia	Celastraceae	Celastrales	Eudicots	Rosids
154 Siphonodon	Celastraceae	Celastrales	Eudicots	Rosids
155 Parnassia	Celastraceae	Celastrales	Eudicots	Rosids
156 Ruptiliocarpon	Lepidobotryaceae	Celastrales	Eudicots	Rosids
157 Lepidobotrys	Lepidobotryaceae	Celastrales	Eudicots	Rosids
158 Juglans	Juglandaceae	Fagales	Eudicots	Rosids
159 Myrica	Myricaceae	Fagales	Eudicots	Rosids
160 Alnus	Betulaceae	Fagales	Eudicots	Rosids
161 Casuarina	Casuarinaceae	Fagales	Eudicots	Rosids
162 Quercus	Fagaceae	Fagales	Eudicots	Rosids
163 Chrysolepis	Fagaceae	Fagales	Eudicots	Rosids
164 Fagus	Fagaceae	Fagales	Eudicots	Rosids
165 Nothofagus	Nothofagaceae	Fagales	Eudicots	Rosids
166 Begonia	Begoniaceae	Cucurbitales	Eudicots	Rosids
167 Datisca	Datiscaceae	Cucurbitales	Eudicots	Rosids
168 Cucurbita	Cucurbitaceae	Cucurbitales	Eudicots	Rosids
169 Cucumis	Cucurbitaceae	Cucurbitales	Eudicots	Rosids
170 Coriaria	Coriariaceae	Cucurbitales	Eudicots	Rosids
171 Anisophyllea	Anisophylleaceae	Cucurbitales	Eudicots	Rosids
172 Pilea	Urticaceae	Rosales	Eudicots	Rosids
173 Urtica	Urticaceae	Rosales	Eudicots	Rosids
174 Morus	Moraceae	Rosales	Eudicots	Rosids
175 Celtis	Cannabaceae	Rosales	Eudicots	Rosids
176 Cannabis	Cannabaceae	Rosales	Eudicots	Rosids
177 Zelkova	Ulmaceae	Rosales	Eudicots	Rosids
178 Ceanothus	Rhamnaceae	Rosales	Eudicots	Rosids
179 Rhamnus	Rhamnaceae	Rosales	Eudicots	Rosids
180 Elaeagnus	Elaeagnaceae	Rosales	Eudicots	Rosids
181 Prunus	Rosaceae	Rosales	Eudicots	Rosids
182 Spiraea	Rosaceae	Rosales	Eudicots	Rosids
183 Pisum	Fabaceae	Fabales	Eudicots	Rosids
184 Medicago	Fabaceae	Fabales	Eudicots	Rosids
185 Cicer	Fabaceae	Fabales	Eudicots	Rosids
186 Lotus	Fabaceae	Fabales	Eudicots	Rosids
187 Glycine	Fabaceae	Fabales	Eudicots	Rosids

188	Albizia	Fabaceae	Fabales	Eudicots	Rosids
189	Polygala	Polygalaceae	Fabales	Eudicots	Rosids
190	Stylobasium	Surianaceae	Fabales	Eudicots	Rosids
191	Quillaja	Quillajaceae	Fabales	Eudicots	Rosids
192	Guaiacum	Zygophyllaceae	Zygophyllales	Eudicots	Rosids
193	Krameria	Krameriaceae	Zygophyllales	Eudicots	Rosids
194	Arabidopsis	Brassicaceae	Brassicales	Eudicots	Rosids
195	Brassica	Brassicaceae	Brassicales	Eudicots	Rosids
196	Capparis	Capparaceae	Brassicales	Eudicots	Rosids
197	Reseda	Resedaceae	Brassicales	Eudicots	Rosids
198	Batis	Bataceae	Brassicales	Eudicots	Rosids
199	Floerkea	Limnanthaceae	Brassicales	Eudicots	Rosids
200	Carica	Caricaceae	Brassicales	Eudicots	Rosids
201	Tropaeolum	Tropaeolaceae	Brassicales	Eudicots	Rosids
202	Helianthemum	Cistaceae	Malvales	Eudicots	Rosids
203	Anisoptera	Dipterocarpaceae	Malvales	Eudicots	Rosids
204	Bixa	Bixaceae	Malvales	Eudicots	Rosids
205	Gossypium	Malvaceae	Malvales	Eudicots	Rosids
206	Sterculia	Malvaceae	Malvales	Eudicots	Rosids
207	Thymelaea	Thymelaeaceae	Malvales	Eudicots	Rosids
208	Tapiscia	Tapisciaceae	Huerteales	Eudicots	Rosids
209	Dipentodon	Dipentodontaceae	Huerteales	Eudicots	Rosids
210	Ailanthus	Simaroubaceae	Sapindales	Eudicots	Rosids
211	Citrus	Rutaceae	Sapindales	Eudicots	Rosids
212	Swietenia	Meliaceae	Sapindales	Eudicots	Rosids
213	Cupaniopsis	Sapindaceae	Sapindales	Eudicots	Rosids
214	Bursera	Burseraceae	Sapindales	Eudicots	Rosids
215	Schinus	Anacardiaceae	Sapindales	Eudicots	Rosids
216	Nitraria	Nitrariaceae	Sapindales	Eudicots	Rosids
217	Picramnia	Phytolaccaceae	Picramniales	Eudicots	Rosids
218	Crossosoma	Crossosomataceae	Crossosomatales	Eudicots	Rosids
219	Stachyurus	Stachyuraceae	Crossosomatales	Eudicots	Rosids
220	Staphylea	Staphyleaceae	Crossosomatales	Eudicots	Rosids
221	Ixerba	Strasburgeriaceae	Crossosomatales	Eudicots	Rosids
222	Strasburgeria	Strasburgeriaceae	Crossosomatales	Eudicots	Rosids
223	Aphloia	Aphloiaceae	Crossosomatales	Eudicots	Rosids
224	Olinia	Penaeaceae	Myrtales	Eudicots	Rosids
225	Crypteronia	Crypteroniaceae	Myrtales	Eudicots	Rosids
226	Clidemia	Melastomataceae	Myrtales	Eudicots	Rosids
227	Eucalyptus	Myrtaceae	Myrtales	Eudicots	Rosids
228	Myrtus	Myrtaceae	Myrtales	Eudicots	Rosids
229	Qualea	Vochysiaceae	Myrtales	Eudicots	Rosids
230	Lythrum	Lythraceae	Myrtales	Eudicots	Rosids
231	Oenothera	Onagraceae	Myrtales	Eudicots	Rosids
232	Terminalia	Combretaceae	Myrtales	Eudicots	Rosids
233	Viviania	Francoaceae	Geraniales	Eudicots	Rosids
234	Melianthus	Francoaceae	Geraniales	Eudicots	Rosids

235	Geranium	Geraniaceae	Geraniales	Eudicots	Rosids
236	Leea	Vitaceae	Vitales	Eudicots	Rosids
237	Vitis	Vitaceae	Vitales	Eudicots	Rosids
238	Myriophyllum	Haloragaceae	Saxifragales	Eudicots	NA
239	Haloragis	Haloragaceae	Saxifragales	Eudicots	NA
240	Penthorum	Penthoraceae	Saxifragales	Eudicots	NA
241	Tetracarpaea	Tetracarpaeaceae	Saxifragales	Eudicots	NA
242	Aphanopetalum	Aphanopetalaceae	Saxifragales	Eudicots	NA
243	Sedum	Crassulaceae	Saxifragales	Eudicots	NA
244	Kalanchoe	Crassulaceae	Saxifragales	Eudicots	NA
245	Saxifraga	Saxifragaceae	Saxifragales	Eudicots	NA
246	Heuchera	Saxifragaceae	Saxifragales	Eudicots	NA
247	Ribes	Grossulariaceae	Saxifragales	Eudicots	NA
248	Itea	Iteaceae	Saxifragales	Eudicots	NA
249	Choristylis	Iteaceae	Saxifragales	Eudicots	NA
250	Paeonia	Paeoniaceae	Saxifragales	Eudicots	NA
251	Soyauxia	Peridiscaceae	Saxifragales	Eudicots	NA
252	Peridiscus	Peridiscaceae	Saxifragales	Eudicots	NA
253	Hamamelis	Hamamelidaceae	Saxifragales	Eudicots	NA
254	Corylopsis	Hamamelidaceae	Saxifragales	Eudicots	NA
255	Exbucklandia	Hamamelidaceae	Saxifragales	Eudicots	NA
256	Rhodoleia	Hamamelidaceae	Saxifragales	Eudicots	NA
257	Cercidiphyllum	Cercidiphyllaceae	Saxifragales	Eudicots	NA
258	Daphniphyllum	Daphniphyllaceae	Saxifragales	Eudicots	NA
259	Liquidambar	Altingiaceae	Saxifragales	Eudicots	NA
260	Altingia	Altingiaceae	Saxifragales	Eudicots	NA
261	Valerianella	Caprifoliaceae	Dipsacales	Eudicots	Asterids
262	Fedia	Caprifoliaceae	Dipsacales	Eudicots	Asterids
263	Centranthus	Caprifoliaceae	Dipsacales	Eudicots	Asterids
264	Valeriana	Caprifoliaceae	Dipsacales	Eudicots	Asterids
265	Nardostachys	Caprifoliaceae	Dipsacales	Eudicots	Asterids
266	Patrinia	Caprifoliaceae	Dipsacales	Eudicots	Asterids
267	Pterocephalodes	Caprifoliaceae	Dipsacales	Eudicots	Asterids
268	Scabiosa	Caprifoliaceae	Dipsacales	Eudicots	Asterids
269	Dipsacus	Caprifoliaceae	Dipsacales	Eudicots	Asterids
270	Triplostegia	Caprifoliaceae	Dipsacales	Eudicots	Asterids
271	Morina	Caprifoliaceae	Dipsacales	Eudicots	Asterids
272	Cryptothladia	Caprifoliaceae	Dipsacales	Eudicots	Asterids
273	Acanthocalyx	Caprifoliaceae	Dipsacales	Eudicots	Asterids
274	Zabelia	Caprifoliaceae	Dipsacales	Eudicots	Asterids
275	Dipelta	Caprifoliaceae	Dipsacales	Eudicots	Asterids
276	Kolkwitzia	Caprifoliaceae	Dipsacales	Eudicots	Asterids
277	Abelia	Caprifoliaceae	Dipsacales	Eudicots	Asterids
278	Linnaea	Caprifoliaceae	Dipsacales	Eudicots	Asterids
279	Leycesteria	Caprifoliaceae	Dipsacales	Eudicots	Asterids
280	Triosteum	Caprifoliaceae	Dipsacales	Eudicots	Asterids
281	Symphoricarpos	Caprifoliaceae	Dipsacales	Eudicots	Asterids

282 Lonicera	Caprifoliaceae	Dipsacales	Eudicots	Asterids
283 Heptacodium	Caprifoliaceae	Dipsacales	Eudicots	Asterids
284 Diervilla	Caprifoliaceae	Dipsacales	Eudicots	Asterids
285 Weigela	Caprifoliaceae	Dipsacales	Eudicots	Asterids
286 Adoxa	Adoxaceae	Dipsacales	Eudicots	Asterids
287 Tetradoxa	Adoxaceae	Dipsacales	Eudicots	Asterids
288 Sinadoxa	Adoxaceae	Dipsacales	Eudicots	Asterids
289 Sambucus	Adoxaceae	Dipsacales	Eudicots	Asterids
290 Viburnum	Adoxaceae	Dipsacales	Eudicots	Asterids
291 Sphenostemon	Paracryphiaceae	Paracryphiales	Eudicots	Asterids
292 Paracryphia	Paracryphiaceae	Paracryphiales	Eudicots	Asterids
293 Quintinia	Paracryphiaceae	Paracryphiales	Eudicots	Asterids
294 Angelica	Apiaceae	Apiales	Eudicots	Asterids
295 Coriandrum	Apiaceae	Apiales	Eudicots	Asterids
296 Anethum	Apiaceae	Apiales	Eudicots	Asterids
297 Apium	Apiaceae	Apiales	Eudicots	Asterids
298 Daucus	Apiaceae	Apiales	Eudicots	Asterids
299 Heteromorpha	Apiaceae	Apiales	Eudicots	Asterids
300 Sanicula	Apiaceae	Apiales	Eudicots	Asterids
301 Arctopus	Apiaceae	Apiales	Eudicots	Asterids
302 Azorella	Apiaceae	Apiales	Eudicots	Asterids
303 Platysace	Apiaceae	Apiales	Eudicots	Asterids
304 Mackinlaya	Apiaceae	Apiales	Eudicots	Asterids
305 Delarbrea	Myodocarpaceae	Apiales	Eudicots	Asterids
306 Myodocarpus	Myodocarpaceae	Apiales	Eudicots	Asterids
307 Pseudopanax	Araliaceae	Apiales	Eudicots	Asterids
308 Tetraplasandra	Araliaceae	Apiales	Eudicots	Asterids
309 Polyscias	Araliaceae	Apiales	Eudicots	Asterids
310 Tetrapanax	Araliaceae	Apiales	Eudicots	Asterids
311 Schefflera	Araliaceae	Apiales	Eudicots	Asterids
312 Hedera	Araliaceae	Apiales	Eudicots	Asterids
313 Cussonia	Araliaceae	Apiales	Eudicots	Asterids
314 Aralia	Araliaceae	Apiales	Eudicots	Asterids
315 Panax	Araliaceae	Apiales	Eudicots	Asterids
316 Hydrocotyle	Araliaceae	Apiales	Eudicots	Asterids
317 Pittosporum	Pittosporaceae	Apiales	Eudicots	Asterids
318 Sollya	Pittosporaceae	Apiales	Eudicots	Asterids
319 Griselinia	Griseliniaceae	Apiales	Eudicots	Asterids
320 Melanophylla	Torricelliaceae	Apiales	Eudicots	Asterids
321 Torricellia	Torricelliaceae	Apiales	Eudicots	Asterids
322 Aralidium	Torricelliaceae	Apiales	Eudicots	Asterids
323 Pennantia	Pennantiaceae	Apiales	Eudicots	Asterids
324 Desfontainia	Columelliaceae	Bruniales	Eudicots	Asterids
325 Columellia	Columelliaceae	Bruniales	Eudicots	Asterids
326 Berzelia	Bruniaceae	Bruniales	Eudicots	Asterids
327 Brunia	Bruniaceae	Bruniales	Eudicots	Asterids
328 Forgesia	Escalloniaceae	Escalloniales	Eudicots	Asterids

329 Valdivia	Escalloniaceae	Escalloniales	Eudicots	Asterids
330 Escallonia	Escalloniaceae	Escalloniales	Eudicots	Asterids
331 Eremosyne	Escalloniaceae	Escalloniales	Eudicots	Asterids
332 Anopterus	Escalloniaceae	Escalloniales	Eudicots	Asterids
333 Tribeles	Escalloniaceae	Escalloniales	Eudicots	Asterids
334 Polyosma	Escalloniaceae	Escalloniales	Eudicots	Asterids
335 Helianthus	Asteraceae	Asterales	Eudicots	Asterids
336 Guizotia	Asteraceae	Asterales	Eudicots	Asterids
337 Tagetes	Asteraceae	Asterales	Eudicots	Asterids
338 Lactuca	Asteraceae	Asterales	Eudicots	Asterids
339 Cichorium	Asteraceae	Asterales	Eudicots	Asterids
340 Tragopogon	Asteraceae	Asterales	Eudicots	Asterids
341 Echinops	Asteraceae	Asterales	Eudicots	Asterids
342 Gerbera	Asteraceae	Asterales	Eudicots	Asterids
343 Barnadesia	Asteraceae	Asterales	Eudicots	Asterids
344 Acicarpha	Calyceraceae	Asterales	Eudicots	Asterids
345 Boopis	Calyceraceae	Asterales	Eudicots	Asterids
346 Moschopsis	Calyceraceae	Asterales	Eudicots	Asterids
347 Goodenia	Goodeniaceae	Asterales	Eudicots	Asterids
348 Scaevola	Goodeniaceae	Asterales	Eudicots	Asterids
349 Dampiera	Goodeniaceae	Asterales	Eudicots	Asterids
350 Fauria	Menyanthaceae	Asterales	Eudicots	Asterids
351 Menyanthes	Menyanthaceae	Asterales	Eudicots	Asterids
352 Villarsia	Menyanthaceae	Asterales	Eudicots	Asterids
353 Nymphoides	Menyanthaceae	Asterales	Eudicots	Asterids
354 Stylidium	Stylidiaceae	Asterales	Eudicots	Asterids
355 Forstera	Stylidiaceae	Asterales	Eudicots	Asterids
356 Donatia	Stylidiaceae	Asterales	Eudicots	Asterids
357 Wittsteinia	Alseuosmiaceae	Asterales	Eudicots	Asterids
358 Alseuosmia	Alseuosmiaceae	Asterales	Eudicots	Asterids
359 Crispiloba	Alseuosmiaceae	Asterales	Eudicots	Asterids
360 Platyspermation	Alseuosmiaceae	Asterales	Eudicots	Asterids
361 Argophyllum	Argophyllaceae	Asterales	Eudicots	Asterids
362 Corokia	Argophyllaceae	Asterales	Eudicots	Asterids
363 Phelline	Phellinaceae	Asterales	Eudicots	Asterids
364 Pentaphragma	Pentaphragmataceae	Asterales	Eudicots	Asterids
365 Lobelia	Campanulaceae	Asterales	Eudicots	Asterids
366 Dialypetalum	Campanulaceae	Asterales	Eudicots	Asterids
367 Pseudonemacladus	Campanulaceae	Asterales	Eudicots	Asterids
368 Trachelium	Campanulaceae	Asterales	Eudicots	Asterids
369 Campanula	Campanulaceae	Asterales	Eudicots	Asterids
370 Cyphia	Campanulaceae	Asterales	Eudicots	Asterids
371 Abrophyllum	Rousseaceae	Asterales	Eudicots	Asterids
372 Cuttsia	Rousseaceae	Asterales	Eudicots	Asterids
373 Carpodetus	Rousseaceae	Asterales	Eudicots	Asterids
374 Roussea	Rousseaceae	Asterales	Eudicots	Asterids
375 Cardiopteris	Cardiopteridaceae	Aquifoliales	Eudicots	Asterids

376	Citronella	Cardiopteridaceae	Aquifoliales	Eudicots	Asterids
377	Gonocaryum	Cardiopteridaceae	Aquifoliales	Eudicots	Asterids
378	Irvingbaileya	Stemonuraceae	Aquifoliales	Eudicots	Asterids
379	Gomphandra	Stemonuraceae	Aquifoliales	Eudicots	Asterids
380	Grisollea	Stemonuraceae	Aquifoliales	Eudicots	Asterids
381	Phyllonoma	Phyllonomaceae	Aquifoliales	Eudicots	Asterids
382	Helwingia	Helwingiaceae	Aquifoliales	Eudicots	Asterids
383	Ilex	Aquifoliaceae	Aquifoliales	Eudicots	Asterids
384	Phryma	Phrymaceae	Lamiales	Eudicots	Asterids
385	Lamium	Lamiaceae	Lamiales	Eudicots	Asterids
386	Paulownia	Paulowniaceae	Lamiales	Eudicots	Asterids
387	Pedicularis	Orobanchaceae	Lamiales	Eudicots	Asterids
388	Verbena	Verbenaceae	Lamiales	Eudicots	Asterids
389	Thomandersia	Thomandersiaceae	Lamiales	Eudicots	Asterids
390	Acanthus	Acanthaceae	Lamiales	Eudicots	Asterids
391	Pinguicula	Lentibulariaceae	Lamiales	Eudicots	Asterids
392	Byblis	Byblidaceae	Lamiales	Eudicots	Asterids
393	Catalpa	Bignoniaceae	Lamiales	Eudicots	Asterids
394	Martynia	Martyniaceae	Lamiales	Eudicots	Asterids
395	Sesamum	Pedaliaceae	Lamiales	Eudicots	Asterids
396	Scrophularia	Scrophulariaceae	Lamiales	Eudicots	Asterids
397	Antirrhinum	Plantaginaceae	Lamiales	Eudicots	Asterids
398	Halleria	Stilbaceae	Lamiales	Eudicots	Asterids
399	Saintpaulia	Gesneriaceae	Lamiales	Eudicots	Asterids
400	Calceolaria	Calceolariaceae	Lamiales	Eudicots	Asterids
401	Peltanthera	Peltantheraceae	Lamiales	Eudicots	Asterids
402	Polypremum	Tetrachondraceae	Lamiales	Eudicots	Asterids
403	Syringa	Oleaceae	Lamiales	Eudicots	Asterids
404	Jasminum	Oleaceae	Lamiales	Eudicots	Asterids
405	Plocosperma	Plocospermataceae	Lamiales	Eudicots	Asterids
406	Atropa	Solanaceae	Solanales	Eudicots	Asterids
407	Solanum	Solanaceae	Solanales	Eudicots	Asterids
408	Nicotiana	Solanaceae	Solanales	Eudicots	Asterids
409	Cuscuta	Convolvulaceae	Solanales	Eudicots	Asterids
410	Ipomoea	Convolvulaceae	Solanales	Eudicots	Asterids
411	Hydrolea	Hydroleaceae	Solanales	Eudicots	Asterids
412	Sphenoclea	Sphenocleaceae	Solanales	Eudicots	Asterids
413	Montinia	Montiniaceae	Solanales	Eudicots	Asterids
414	Exacum	Gentianaceae	Gentianales	Eudicots	Asterids
415	Gentiana	Gentianaceae	Gentianales	Eudicots	Asterids
416	Nerium	Apocynaceae	Gentianales	Eudicots	Asterids
417	Strychnos	Loganiaceae	Gentianales	Eudicots	Asterids
418	Gelsemium	Gelsemiaceae	Gentianales	Eudicots	Asterids
419	Coffea	Rubiaceae	Gentianales	Eudicots	Asterids
420	Galium	Rubiaceae	Gentianales	Eudicots	Asterids
421	Luculia	Rubiaceae	Gentianales	Eudicots	Asterids
422	Ehretia	Boraginaceae	Boraginales	Eudicots	Asterids

423	Hydrophyllum	Boraginaceae	Boraginales	Eudicots	Asterids
424	Borago	Boraginaceae	Boraginales	Eudicots	Asterids
425	Vahlia	Vahliaceae	Vahliales	Eudicots	Asterids
426	Oncotheca	Oncothecaceae	Icacinales	Eudicots	Asterids
427	Eucommia	Eucommiaceae	Garryales	Eudicots	Asterids
428	Garrya	Garryaceae	Garryales	Eudicots	Asterids
429	Icacina	Icacinaceae	Icacinales	Eudicots	Asterids
430	Arbutus	Ericaceae	Ericales	Eudicots	Asterids
431	Cyrilla	Cyrillaceae	Ericales	Eudicots	Asterids
432	Clethra	Clethraceae	Ericales	Eudicots	Asterids
433	Sarracenia	Sarraceniaceae	Ericales	Eudicots	Asterids
434	Actinidia	Actinidiaceae	Ericales	Eudicots	Asterids
435	Styrax	Styracaceae	Ericales	Eudicots	Asterids
436	Galax	Diapensiaceae	Ericales	Eudicots	Asterids
437	Camellia	Theaceae	Ericales	Eudicots	Asterids
438	Primula	Primulaceae	Ericales	Eudicots	Asterids
439	Androsace	Primulaceae	Ericales	Eudicots	Asterids
440	Clavija	Primulaceae	Ericales	Eudicots	Asterids
441	Maesa	Primulaceae	Ericales	Eudicots	Asterids
442	Diospyros	Ebenaceae	Ericales	Eudicots	Asterids
443	Ternstroemia	Pentaphylacaceae	Ericales	Eudicots	Asterids
444	Manilkara	Sapotaceae	Ericales	Eudicots	Asterids
445	Idria	Fouquieriaceae	Ericales	Eudicots	Asterids
446	Fouquieria	Fouquieriaceae	Ericales	Eudicots	Asterids
447	Polemonium	Polemoniaceae	Ericales	Eudicots	Asterids
448	Couroupita	Lecythidaceae	Ericales	Eudicots	Asterids
449	Marcgravia	Marcgraviaceae	Ericales	Eudicots	Asterids
450	Tetramerista	Tetrameristaceae	Ericales	Eudicots	Asterids
451	Impatiens	Balsaminaceae	Ericales	Eudicots	Asterids
452	Mentzelia	Loasaceae	Cornales	Eudicots	Asterids
453	Petalonyx	Loasaceae	Cornales	Eudicots	Asterids
454	Hydrangea	Hydrangeaceae	Cornales	Eudicots	Asterids
455	Curtisia	Curtisiaceae	Cornales	Eudicots	Asterids
456	Nyssa	Nyssaceae	Cornales	Eudicots	Asterids
457	Cornus	Cornaceae	Cornales	Eudicots	Asterids
458	Halophytum	Halophytaceae	Caryophyllales	Eudicots	NA
459	Basella	Basellaceae	Caryophyllales	Eudicots	NA
460	Alluaudia	Didiereaceae	Caryophyllales	Eudicots	NA
461	Calyptrotheca	Didiereaceae	Caryophyllales	Eudicots	NA
462	Pereskia	Cactaceae	Caryophyllales	Eudicots	NA
463	Opuntia	Cactaceae	Caryophyllales	Eudicots	NA
464	Portulaca	Portulacaceae	Caryophyllales	Eudicots	NA
465	Talinum	Talinaceae	Caryophyllales	Eudicots	NA
466	Claytonia	Montiaceae	Caryophyllales	Eudicots	NA
467	Mollugo	Molluginaceae	Caryophyllales	Eudicots	NA
468	Mirabilis	Nyctaginaceae	Caryophyllales	Eudicots	NA
469	Bougainvillea	Nyctaginaceae	Caryophyllales	Eudicots	NA

470	Rivina	Petiveriaceae	Caryophyllales	Eudicots	NA
471	Phytolacca	Phytolaccaceae	Caryophyllales	Eudicots	NA
472	Sarcobatus	Sarcobataceae	Caryophyllales	Eudicots	NA
473	Gisekia	Gisekiaceae	Caryophyllales	Eudicots	NA
474	Lampranthus	Aizoaceae	Caryophyllales	Eudicots	NA
475	Delosperma	Aizoaceae	Caryophyllales	Eudicots	NA
476	Barbeuia	Barbeuiaceae	Caryophyllales	Eudicots	NA
477	Limeum	Limeaceae	Caryophyllales	Eudicots	NA
478	Stegnosperma	Stegnospermataceae	Caryophyllales	Eudicots	NA
479	Celosia	Amaranthaceae	Caryophyllales	Eudicots	NA
480	Spinacia	Amaranthaceae	Caryophyllales	Eudicots	NA
481	Beta	Amaranthaceae	Caryophyllales	Eudicots	NA
482	Phaulothamnus	Achatocarpaceae	Caryophyllales	Eudicots	NA
483	Stellaria	Caryophyllaceae	Caryophyllales	Eudicots	NA
484	Physena	Physenaceae	Caryophyllales	Eudicots	NA
485	Asteropeia	Asteropeiaceae	Caryophyllales	Eudicots	NA
486	Simmondsia	Simmondsiaceae	Caryophyllales	Eudicots	NA
487	Rhabdodendron	Ericaceae	Caryophyllales	Eudicots	NA
488	Bistorta	Polygonaceae	Caryophyllales	Eudicots	NA
489	Fagopyrum	Polygonaceae	Caryophyllales	Eudicots	NA
490	Polygonum	Polygonaceae	Caryophyllales	Eudicots	NA
491	Limonium	Plumbaginaceae	Caryophyllales	Eudicots	NA
492	Plumbago	Plumbaginaceae	Caryophyllales	Eudicots	NA
493	Tamarix	Tamaricaceae	Caryophyllales	Eudicots	NA
494	Frankenia	Frankeniaceae	Caryophyllales	Eudicots	NA
495	Triphyophyllum	Dioncophyllaceae	Caryophyllales	Eudicots	NA
496	Dioncophyllum	Dioncophyllaceae	Caryophyllales	Eudicots	NA
497	Ancistrocladus	Ancistrocladaceae	Caryophyllales	Eudicots	NA
498	Drosophyllum	Drosophyllaceae	Caryophyllales	Eudicots	NA
499	Drosera	Droseraceae	Caryophyllales	Eudicots	NA
500	Nepenthes	Nepenthaceae	Caryophyllales	Eudicots	NA
501	Aextoxicon	Aextoxicaceae	Berberidopsidales	Eudicots	NA
502	Berberidopsis	Berberidopsidaceae	Berberidopsidales	Eudicots	NA
503	Santalum	Santalaceae	Santalales	Eudicots	NA
504	Opilia	Opiliaceae	Santalales	Eudicots	NA
505	Gaiadendron	Loranthaceae	Santalales	Eudicots	NA
506	Schoepfia	Schoepfiaceae	Santalales	Eudicots	NA
507	Ximenia	Olacaceae	Santalales	Eudicots	NA
508	Heisteria	Olacaceae	Santalales	Eudicots	NA
509	Dillenia	Dilleniaceae	Dilleniales	Eudicots	NA
510	Hibbertia	Dilleniaceae	Dilleniales	Eudicots	NA
511	Tetracera	Dilleniaceae	Dilleniales	Eudicots	NA
512	Myrothamnus	Myrothamnaceae	Gunnerales	Eudicots	NA
513	Gunnera	Gunneraceae	Gunnerales	Eudicots	NA
514	Buxus	Buxaceae	Buxales	Eudicots	NA
515	Pachysandra	Buxaceae	Buxales	Eudicots	NA
516	Didymeles	Buxaceae	Buxales	Eudicots	NA

517	Tetracentron	Trochodendraceae	Trochodendrales	Eudicots	NA
518	Trochodendron	Trochodendraceae	Trochodendrales	Eudicots	NA
519	Petrophile	Proteaceae	Proteales	Eudicots	NA
520	Roupala	Proteaceae	Proteales	Eudicots	NA
521	Platanus	Platanaceae	Proteales	Eudicots	NA
522	Nelumbo	Nelumbonaceae	Proteales	Eudicots	NA
523	Sabia	Sabiaceae	Proteales	Eudicots	NA
524	Meliosma	Sabiaceae	Proteales	Eudicots	NA
525	Ranunculus	Ranunculaceae	Ranunculales	Eudicots	NA
526	Glaucidium	Ranunculaceae	Ranunculales	Eudicots	NA
527	Hydrastis	Ranunculaceae	Ranunculales	Eudicots	NA
528	Nandina	Berberidaceae	Ranunculales	Eudicots	NA
529	Caulophyllum	Berberidaceae	Ranunculales	Eudicots	NA
530	Menispermum	Menispermaceae	Ranunculales	Eudicots	NA
531	Tinospora	Menispermaceae	Ranunculales	Eudicots	NA
532	Decaisnea	Lardizabalaceae	Ranunculales	Eudicots	NA
533	Lardizabala	Lardizabalaceae	Ranunculales	Eudicots	NA
534	Sargentodoxa	Lardizabalaceae	Ranunculales	Eudicots	NA
535	Circaeaster	Circaeasteraceae	Ranunculales	Eudicots	NA
536	Hypecoum	Papaveraceae	Ranunculales	Eudicots	NA
537	Dicentra	Papaveraceae	Ranunculales	Eudicots	NA
538	Eschscholzia	Papaveraceae	Ranunculales	Eudicots	NA
539	Euptelea	Eupteleaceae	Ranunculales	Eudicots	NA
540	Ceratophyllum	Ceratophyllaceae	Ceratophyllales	Ceratophyllidae	NA
541	Saccharum	Poaceae	Poales	Monocots	NA
542	Zea	Poaceae	Poales	Monocots	NA
543	Oryza	Poaceae	Poales	Monocots	NA
544	Hordeum	Poaceae	Poales	Monocots	NA
545	Stegolepis	Rapateaceae	Poales	Monocots	NA
546	Typha	Typhaceae	Poales	Monocots	NA
547	Sparganium	Typhaceae	Poales	Monocots	NA
548	Vriesea	Bromeliaceae	Poales	Monocots	NA
549	Maranta	Marantaceae	Zingiberales	Monocots	NA
550	Musa	Musaceae	Zingiberales	Monocots	NA
551	Strelitzia	Strelitziaceae	Zingiberales	Monocots	NA
552	Philydrum	Philydraceae	Commelinales	Monocots	NA
553	Tradescantia	Commelinaceae	Commelinales	Monocots	NA
554	Chamaedorea	Arecaceae	Arecales	Monocots	NA
555	Elaeis	Arecaceae	Arecales	Monocots	NA
556	Asparagus	Asparagaceae	Asparagales	Monocots	NA
557	Lomandra	Asparagaceae	Asparagales	Monocots	NA
558	Beaucarnea	Asparagaceae	Asparagales	Monocots	NA
559	Agave	Asparagaceae	Asparagales	Monocots	NA
560	Yucca	Asparagaceae	Asparagales	Monocots	NA
561	Allium	Amaryllidaceae	Asparagales	Monocots	NA
562	Xanthorrhoea	Asphodelaceae	Asparagales	Monocots	NA
563	Iris	Iridaceae	Asparagales	Monocots	NA

564	Blandfordia	Blandfordiaceae	Asparagales	Monocots	NA
565	Oncidium	Orchidaceae	Asparagales	Monocots	NA
566	Phalaenopsis	Orchidaceae	Asparagales	Monocots	NA
567	Smilax	Smilacaceae	Liliales	Monocots	NA
568	Lilium	Liliaceae	Liliales	Monocots	NA
569	Trillium	Melanthiaceae	Liliales	Monocots	NA
570	Carludovica	Cyclanthaceae	Pandanales	Monocots	NA
571	Croomia	Stemonaceae	Pandanales	Monocots	NA
572	Dioscorea	Dioscoreaceae	Dioscoreales	Monocots	NA
573	Hydrocharis	Hydrocharitaceae	Alismatales	Monocots	NA
574	Alisma	Alismataceae	Alismatales	Monocots	NA
575	Triglochin	Juncaginaceae	Alismatales	Monocots	NA
576	Potamogeton	Potamogetonaceae	Alismatales	Monocots	NA
577	Pleea	Tofieldiaceae	Alismatales	Monocots	NA
578	Tofieldia	Tofieldiaceae	Alismatales	Monocots	NA
579	Xanthosoma	Araceae	Alismatales	Monocots	NA
580	Spathiphyllum	Araceae	Alismatales	Monocots	NA
581	Lemna	Araceae	Alismatales	Monocots	NA
582	Orontium	Araceae	Alismatales	Monocots	NA
583	Acorus	Acoraceae	Acorales	Monocots	NA
584	Peumus	Monimiaceae	Laurales	Magnoliids	NA
585	Hedycarya	Monimiaceae	Laurales	Magnoliids	NA
586	Hernandia	Hernandiaceae	Laurales	Magnoliids	NA
587	Gyrocarpus	Hernandiaceae	Laurales	Magnoliids	NA
588	Cryptocarya	Lauraceae	Laurales	Magnoliids	NA
589	Laurus	Lauraceae	Laurales	Magnoliids	NA
590	Atherosperma	Atherospermataceae	Laurales	Magnoliids	NA
591	Daphnandra	Atherospermataceae	Laurales	Magnoliids	NA
592	Gomortega	Gomortegaceae	Laurales	Magnoliids	NA
593	Siparuna	Siparunaceae	Laurales	Magnoliids	NA
594	Idiospermum	Calycanthaceae	Laurales	Magnoliids	NA
595	Calycanthus	Calycanthaceae	Laurales	Magnoliids	NA
596	Annona	Annonaceae	Magnoliales	Magnoliids	NA
597	Cananga	Annonaceae	Magnoliales	Magnoliids	NA
598	Eupomatia	Eupomatiaceae	Magnoliales	Magnoliids	NA
599	Galbulimima	Himantandraceae	Magnoliales	Magnoliids	NA
600	Magnolia	Magnoliaceae	Magnoliales	Magnoliids	NA
601	Liriodendron	Magnoliaceae	Magnoliales	Magnoliids	NA
602	Myristica	Myristicaceae	Magnoliales	Magnoliids	NA
603	Mauloutchia	Myristicaceae	Magnoliales	Magnoliids	NA
604	Degeneria	Degeneriaceae	Magnoliales	Magnoliids	NA
605	Houttuynia	Saururaceae	Piperales	Magnoliids	NA
606	Saururus	Saururaceae	Piperales	Magnoliids	NA
607	Peperomia	Piperaceae	Piperales	Magnoliids	NA
608	Piper	Piperaceae	Piperales	Magnoliids	NA
609	Thottea	Aristolochiaceae	Piperales	Magnoliids	NA
610	Aristolochia	Aristolochiaceae	Piperales	Magnoliids	NA

611	Lactoris	Aristolochiaceae	Piperales	Magnoliids	NA
612	Saruma	Aristolochiaceae	Piperales	Magnoliids	NA
613	Tasmannia	Winteraceae	Canellales	Magnoliids	NA
614	Drimys	Winteraceae	Canellales	Magnoliids	NA
615	Takhtajania	Winteraceae	Canellales	Magnoliids	NA
616	Canella	Canellaceae	Canellales	Magnoliids	NA
617	Cinnamodendron	Canellaceae	Canellales	Magnoliids	NA
618	Sarcandra	Chloranthaceae	Chloranthales	NA	NA
619	Chloranthus	Chloranthaceae	Chloranthales	NA	NA
620	Ascarina	Chloranthaceae	Chloranthales	NA	NA
621	Hedyosmum	Chloranthaceae	Chloranthales	NA	NA
622	Schisandra	Schisandraceae	Austrobaileyales	Basal_angiosperms	NA
623	Kadsura	Schisandraceae	Austrobaileyales	Basal_angiosperms	NA
624	Illicium	Schisandraceae	Austrobaileyales	Basal_angiosperms	NA
625	Trimenia	Trimeniaceae	Austrobaileyales	Basal_angiosperms	NA
626	Austrobaileya	Austrobaileyaceae	Austrobaileyales	Basal_angiosperms	NA
627	Brasenia	Cabombaceae	Nymphaeales	Basal_angiosperms	NA
628	Cabomba	Cabombaceae	Nymphaeales	Basal_angiosperms	NA
629	Nymphaea	Nymphaeaceae	Nymphaeales	Basal_angiosperms	NA
630	Nuphar	Nymphaeaceae	Nymphaeales	Basal_angiosperms	NA
631	Trithuria	Hydatellaceae	Nymphaeales	Basal_angiosperms	NA
632	Amborella	Amborellaceae	Amborellales	Basal_angiosperms	NA
633	Gnetum	Gnetaceae	Gnetales	outgroup	Gymnosperms
634	Welwitschia	Welwitschiaceae	Welwitschiales	outgroup	Gymnosperms
635	Podocarpus	Podocarpaceae	Araucariales	outgroup	Gymnosperms
636	Metasequoia	Cupressaceae	Cupressales	outgroup	Gymnosperms
637	Pinus	Pinaceae	Pinales	outgroup	Gymnosperms
638	Cycas	Cycadaceae	Cycadales	outgroup	Gymnosperms
639	Zamia	Zamiaceae	Cycadales	outgroup	Gymnosperms
640	Ginkgo	Ginkgoaceae	Ginkgoales	outgroup	Gymnosperms
641	Psilotum	Psilotaceae	Psilotales	outgroup	Ferns
642	Adiantum	Pteridaceae	Polypodiales	outgroup	Ferns
643	Huperzia	Lycopodiaceae	Lycopodiales	outgroup	Lycophytes
644	Selaginella	Selaginellaceae	Selaginellales	outgroup	Lycophytes

Supplementary Figure 1. Calibrated tree topology.
This figure indicates the nodes which age has been constrained with the derived calibration densities from the BBB analysis. Due to the length of the tree, the topology expands pages 21-25. Pages 21-24 include the tree topology of one clade present in the main calibrated tree topology that was used for timetree inference (if applicable, a collapsed now will be included with the tree topology depicted in the previous page). Page 25 shows the main tree topology with the largest clades collapsed, the outgroups that were used to root the tree, and the root of the tree (Angiosperms).

Subtree of the clade Rosids.

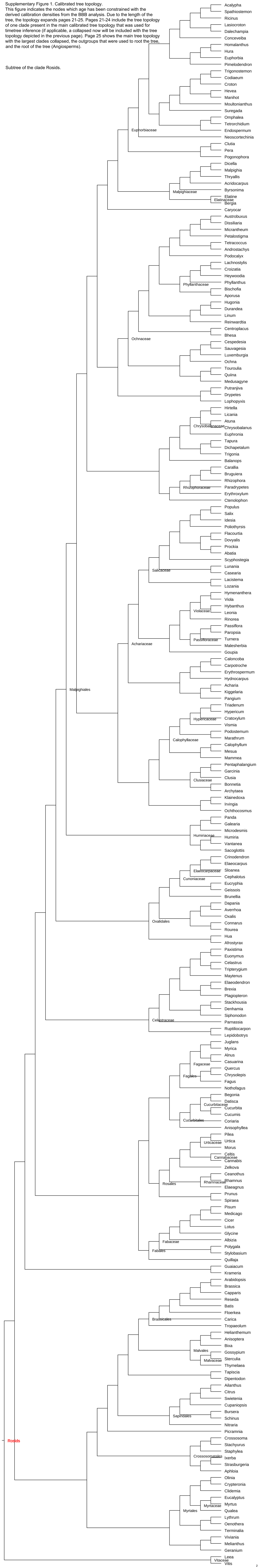

Subtree of the clade Asterids.

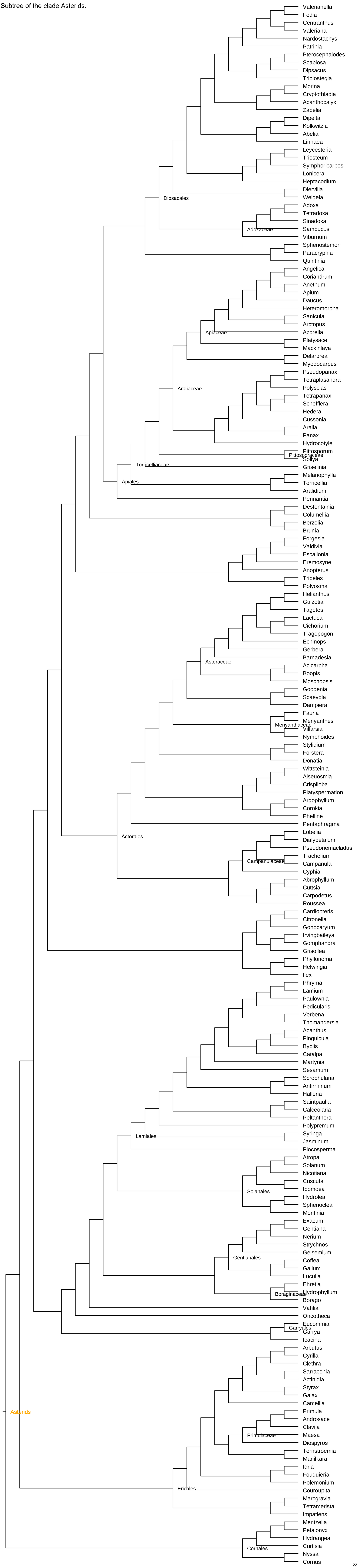

Subtree of the clade Eudicots.

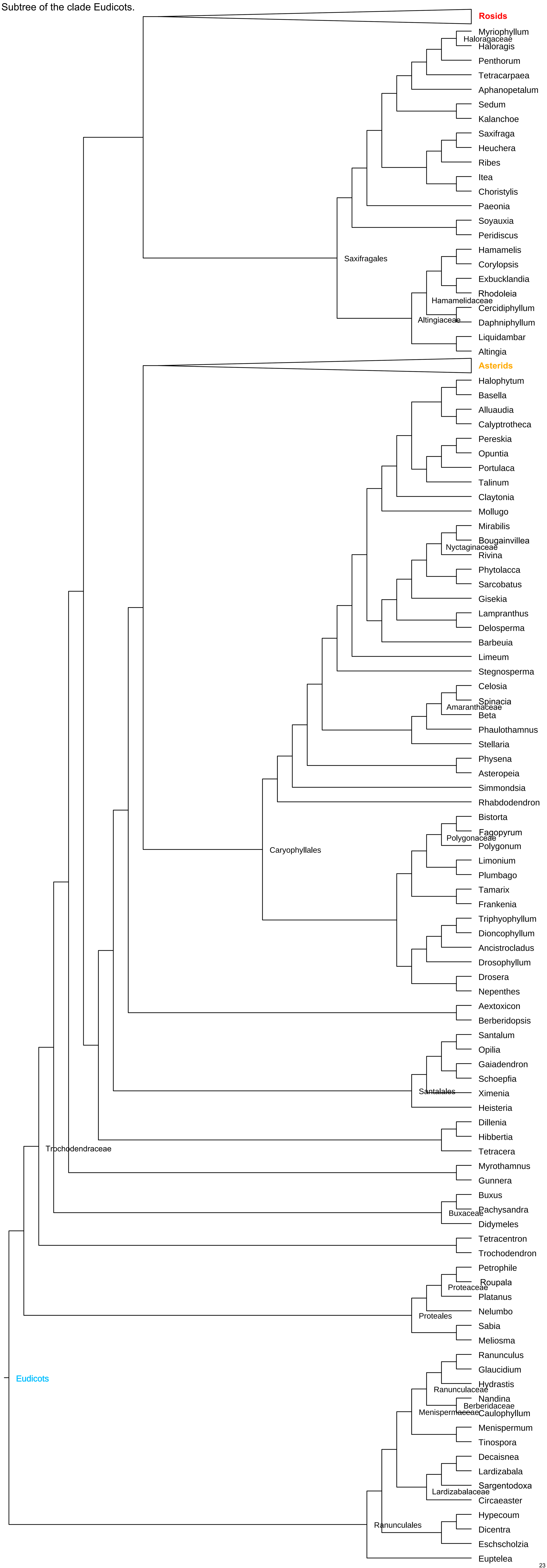

Subtree of the clade Monocots.

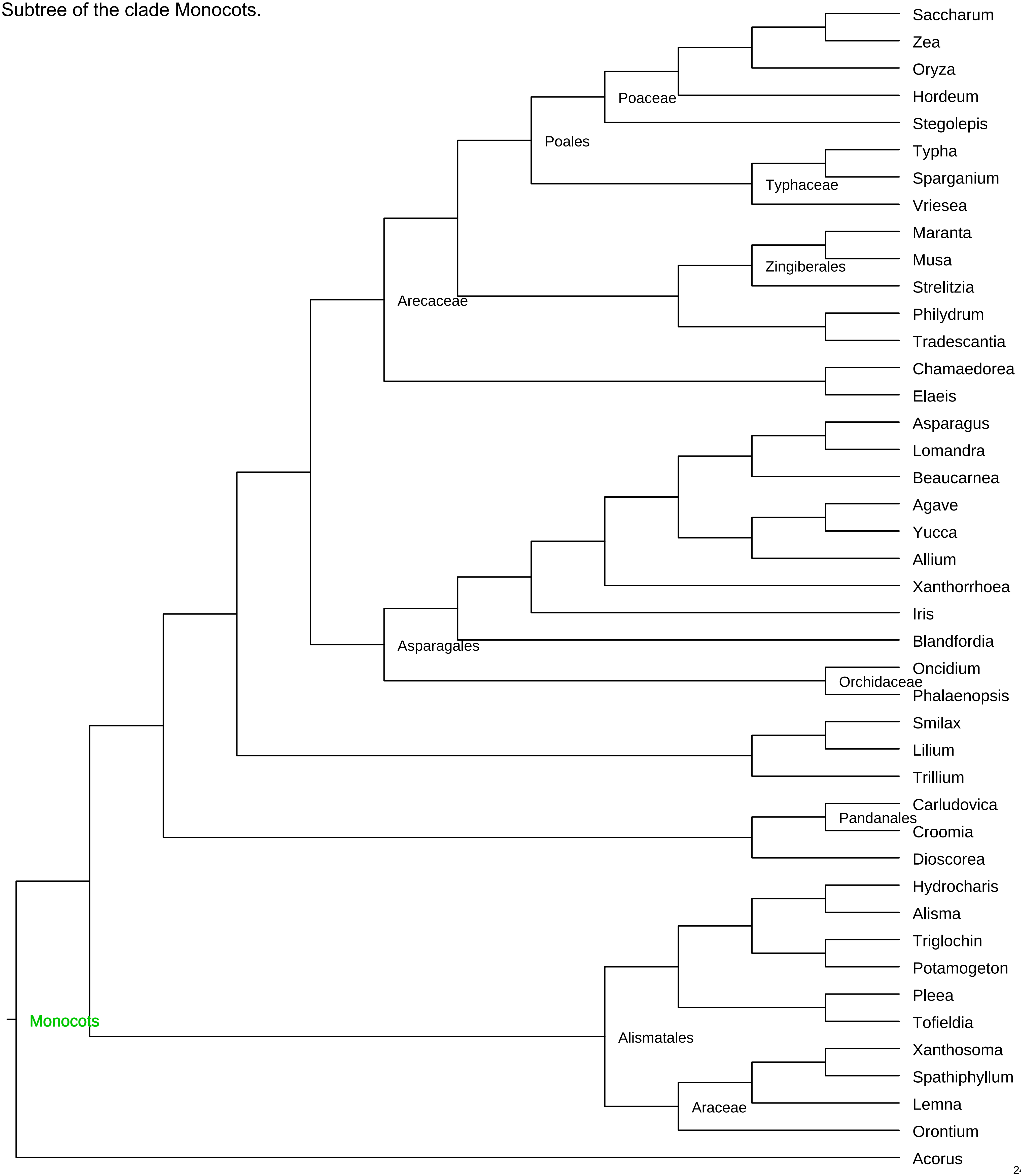

Tree of tracheophytes.

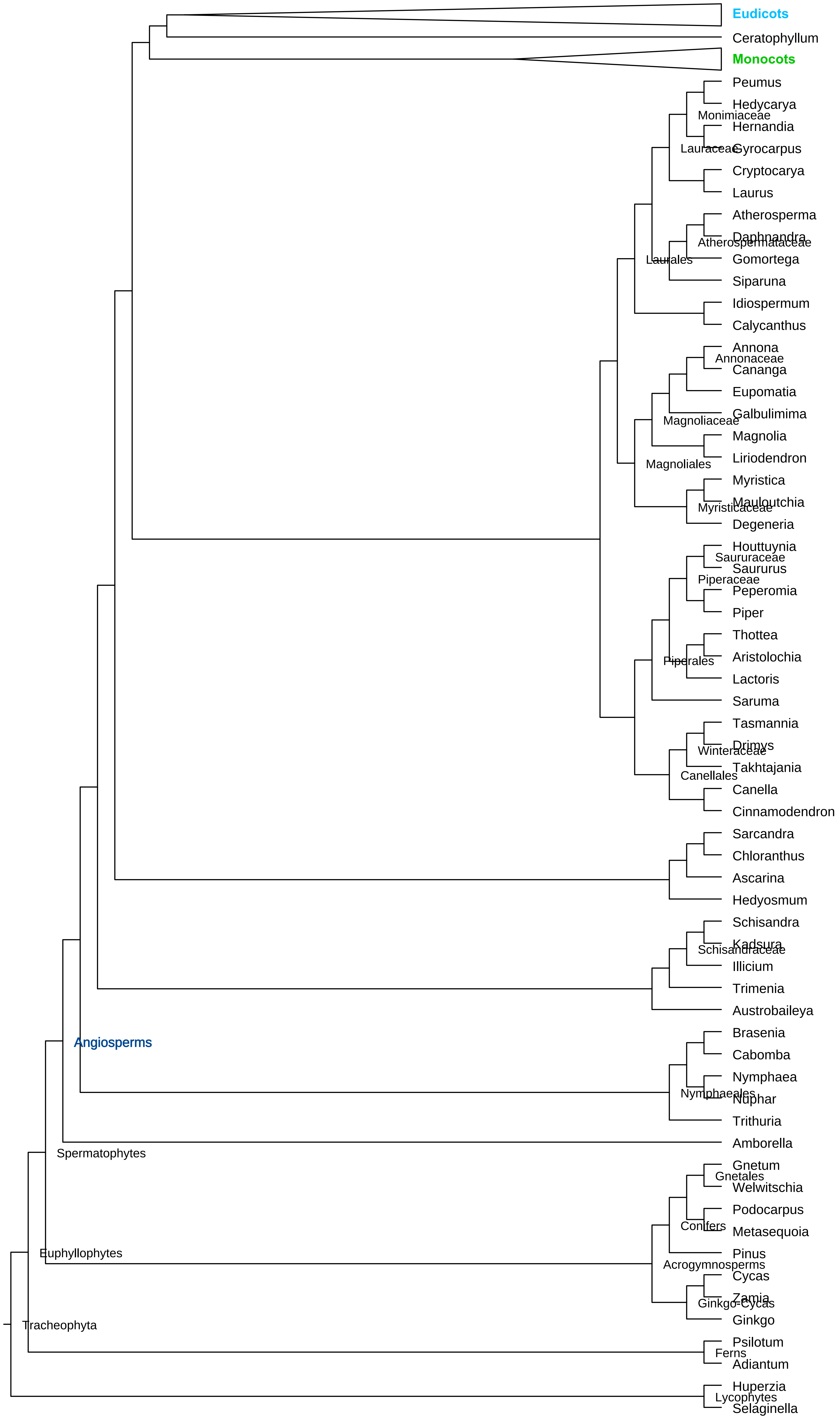

Supplementary Figure 2. The timetree of tracheophytes of three calibration strategies with 95% HPD intervals

Inferred evolutionary timeline of tracheophytes when using the uniform densities with hard bounds (derived from the BBB analysis) as calibration densities during timetree inference. The blue bars show the 95% HPD intervals (bounds are also displayed as node labels) to capture the uncertainty in the estimated posterior time densities.

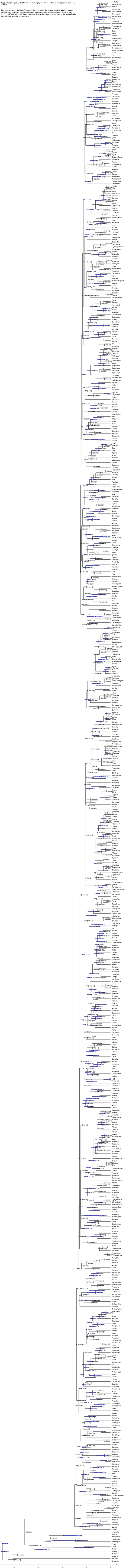

Inferred evolutionary timeline of tracheophytes when using the uniform densities with soft bounds (derived from the BBB analysis, tail percentage set to 2.5% for both upper and lower bounds) as calibration densities during timetree inference. The blue bars show the 95% HPD intervals (bounds are also displayed as node labels) to capture the uncertainty in the estimated posterior time densities.

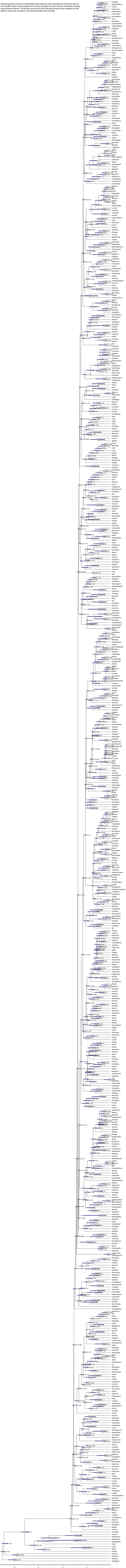

Inferred evolutionary timeline of tracheophytes when using the skew-T distributions (derived from the BBB analysis) as calibration densities during timetree inference. The blue bars show the 95% HPD intervals (bounds are also displayed as node labels) to capture the uncertainty in the estimated posterior time densities.

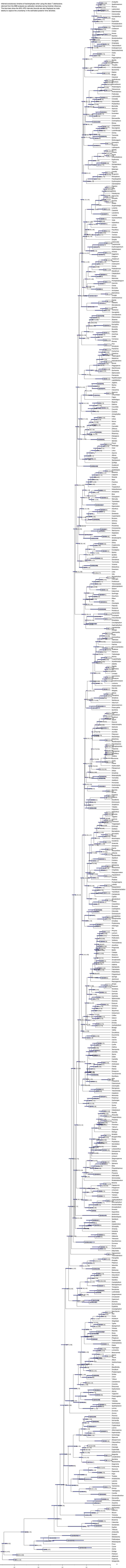

Supplementary Figure 3. Comparison of marginal densities against calibration densities and posterior densities plot.

Comparison of marginal densities (effective priors) against calibration densities (user-specified priors) and posterior densities for those nodes which age had been constrained under each of the different calibration schemes.

Strategy 1: comparison of marginal densities (effective priors) against calibration densities (user-specified priors) and posterior densities for those nodes which age had been constrained with a uniform density with hard bounds.

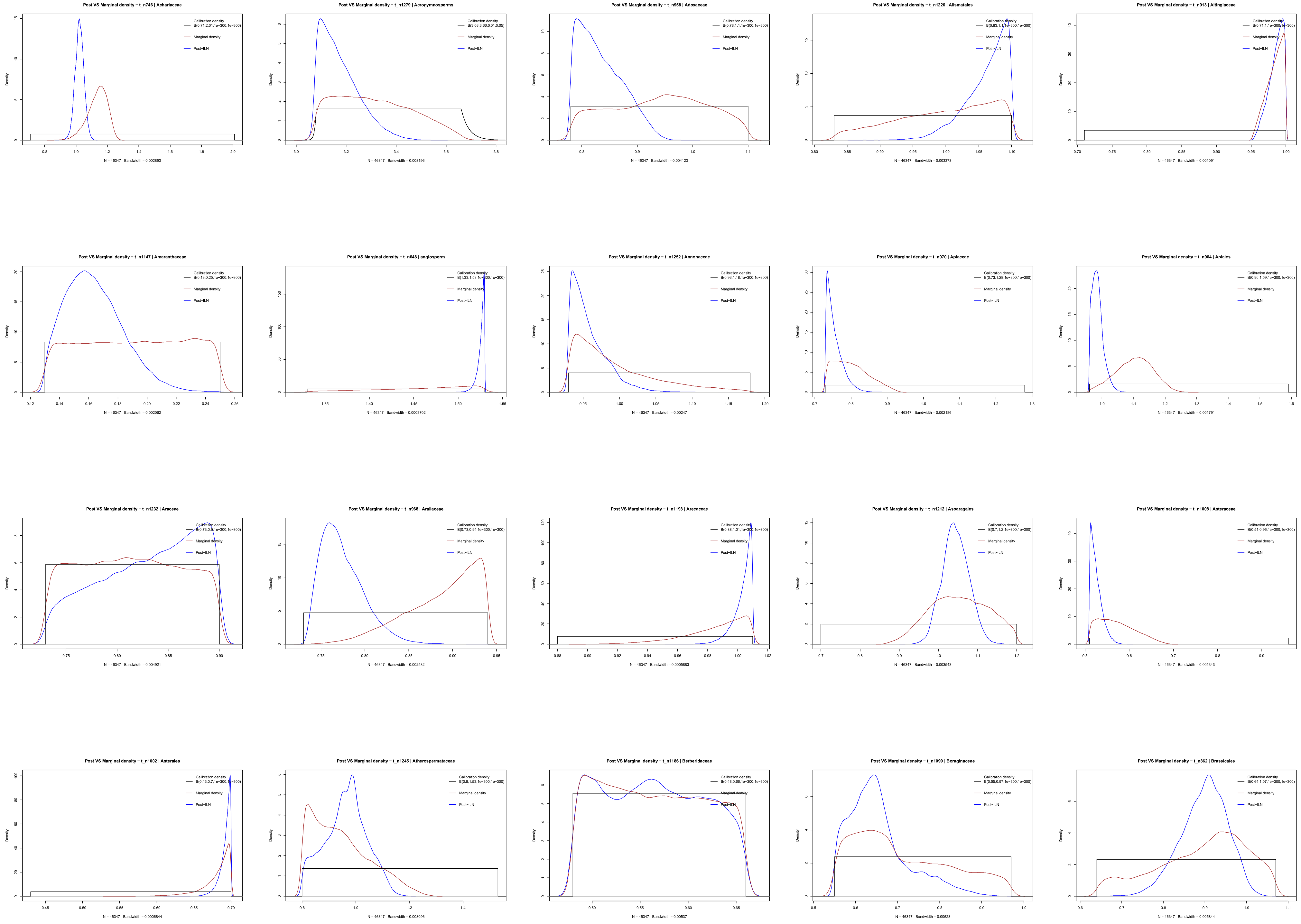

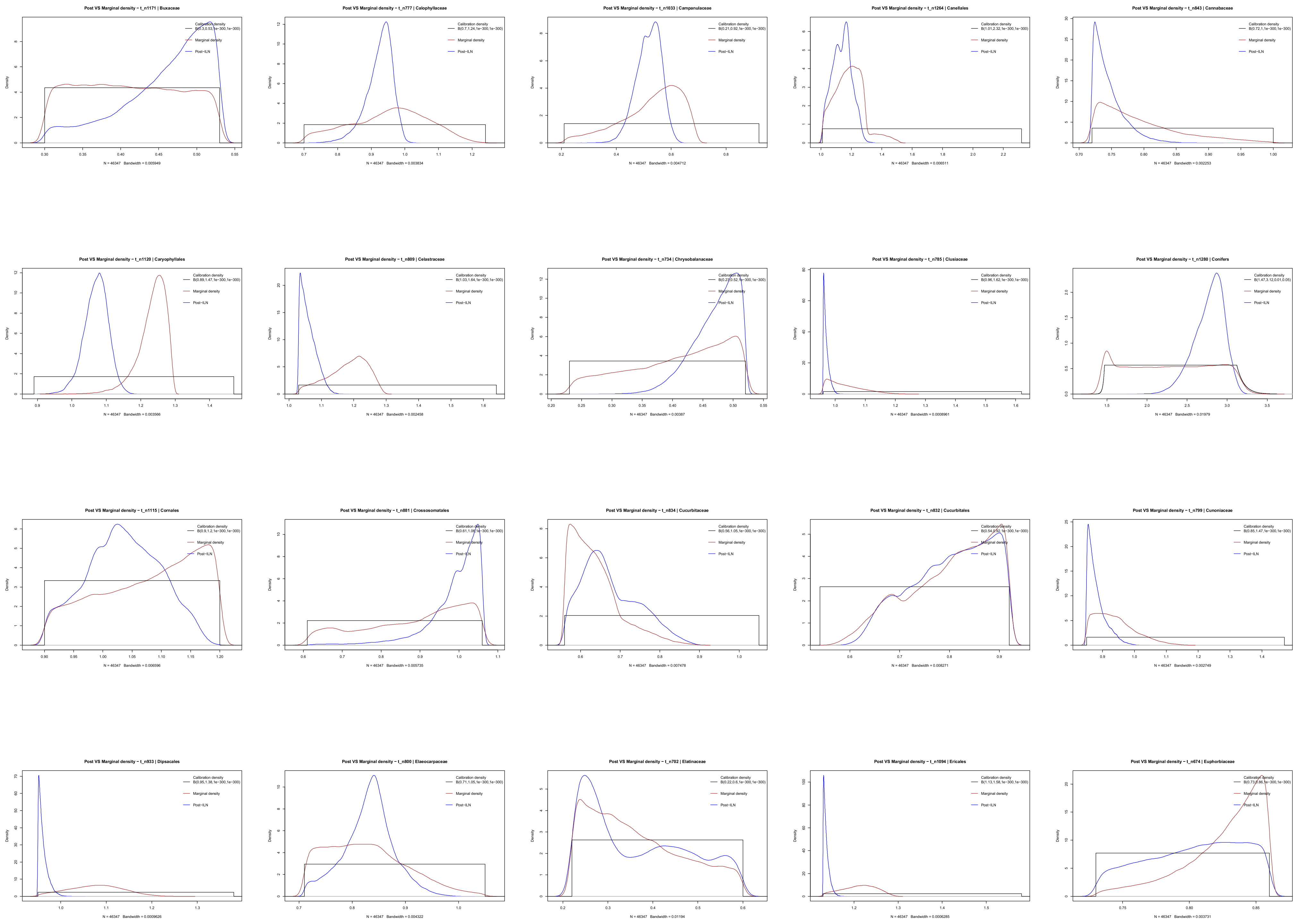
Post VS Marginal density − t_n1171 | Buxaceae
Calibration density
B(0.3,0.53,1e−300,1e−300)
Marginal density
Post−ILN
N = 46347 Bandwidth = 0.005949
Post VS Marginal density − t_n777 | Calophyllaceae
Calibration density
B(0.7,1.24,1e−300,1e−300)
Marginal density
Post−ILN
N = 46347 Bandwidth = 0.003834
Post VS Marginal density − t_n1033 | Campanulaceae
Calibration density
B(0.21,0.92,1e−300,1e−300)
Marginal density
Post−ILN
N = 46347 Bandwidth = 0.004712
Post VS Marginal density − t_n1264 | Canellales
Calibration density
B(1.01,2.32,1e−300,1e−300)
Marginal density
Post−ILN
N = 46347 Bandwidth = 0.006511
Post VS Marginal density − t_n843 | Cannabaceae
Calibration density
B(0.72,1,1e−300,1e−300)
Marginal density
Post−ILN
N = 46347 Bandwidth = 0.002253
Post VS Marginal density − t_n1120 | Caryophyllales
Calibration density
B(0.89,1.47,1e−300,1e−300)
Marginal density
Post−ILN
N = 46347 Bandwidth = 0.003566
Post VS Marginal density − t_n809 | Celastraceae
Calibration density
B(1.03,1.64,1e−300,1e−300)
Marginal density
Post−ILN
N = 46347 Bandwidth = 0.002458
Post VS Marginal density − t_n734 | Chrysobalanaceae
Calibration density
B(0.23,0.52,1e−300,1e−300)
Marginal density
Post−ILN
N = 46347 Bandwidth = 0.00387
Post VS Marginal density − t_n785 | Clusiaceae
Calibration density
B(0.96,1.62,1e−300,1e−300)
Marginal density
Post−ILN
N = 46347 Bandwidth = 0.0008961
Post VS Marginal density − t_n1280 | Conifers
Calibration density
B(1.47,3.12,0.01,0.05)
Marginal density
Post−ILN
N = 46347 Bandwidth = 0.01979
Post VS Marginal density − t_n1115 | Cornales
Calibration density
B(0.9,1.2,1e−300,1e−300)
Marginal density
Post−ILN
N = 46347 Bandwidth = 0.006596
Post VS Marginal density − t_n881 | Crossosomatales
Calibration density
B(0.61,1.06,1e−300,1e−300)
Marginal density
Post−ILN
N = 46347 Bandwidth = 0.005735
Post VS Marginal density − t_n834 | Cucurbitaceae
Calibration density
B(0.56,1.05,1e−300,1e−300)
Marginal density
Post−ILN
N = 46347 Bandwidth = 0.007478
Post VS Marginal density − t_n832 | Cucurbitales
Calibration density
B(0.54,0.92,1e−300,1e−300)
Marginal density
Post−ILN
N = 46347 Bandwidth = 0.008271
Post VS Marginal density − t_n799 | Cunoniaceae
Calibration density
B(0.85,1.47,1e−300,1e−300)
Marginal density
Post−ILN
N = 46347 Bandwidth = 0.002749
Post VS Marginal density − t_n933 | Dipsacales
Calibration density
B(0.95,1.38,1e−300,1e−300)
Marginal density
Post−ILN
N = 46347 Bandwidth = 0.0009626
Post VS Marginal density − t_n800 | Elaeocarpaceae
Calibration density
B(0.71,1.05,1e−300,1e−300)
Marginal density
Post−ILN
N = 46347 Bandwidth = 0.004322
Post VS Marginal density − t_n702 | Elatinaceae
Calibration density
B(0.22,0.6,1e−300,1e−300)
Marginal density
Post−ILN
N = 46347 Bandwidth = 0.01194
Post VS Marginal density − t_n1094 | Ericales
Calibration density
B(1.13,1.58,1e−300,1e−300)
Marginal density
Post−ILN
N = 46347 Bandwidth = 0.0006285
Post VS Marginal density − t_n674 | Euphorbiaceae
Calibration density
B(0.73,0.86,1e−300,1e−300)
Marginal density
Post−ILN
N = 46347 Bandwidth = 0.003731

Post VS Marginal density − t_n646 | Euphyllophytes

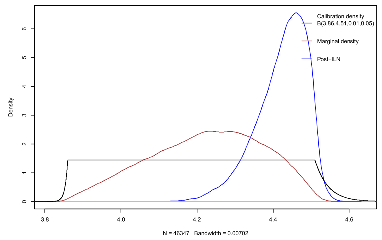

Post VS Marginal density − t_n848 | Fabaceae

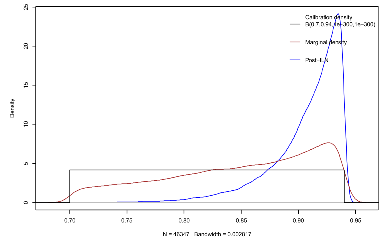

Post VS Marginal density − t_n847 | Fabales

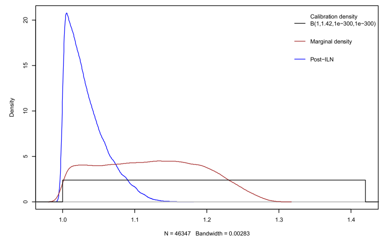

Post VS Marginal density − t_n826 | Fagaceae

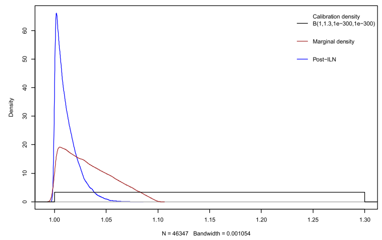

Post VS Marginal density − t_n825 | Fagales

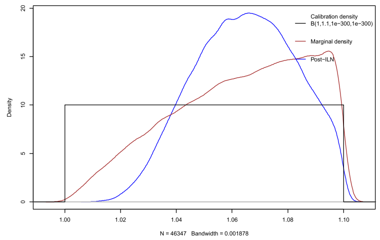

Post VS Marginal density − t_n1286 | Ferns

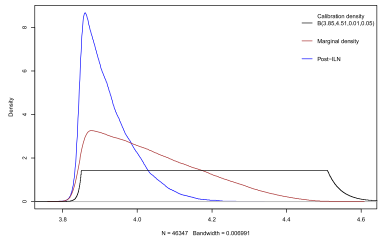

Post VS Marginal density − t_n1093 | Garryales

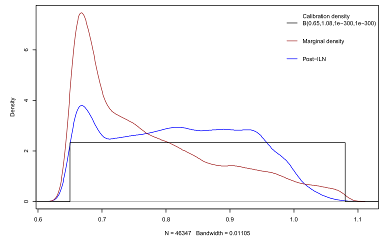

Post VS Marginal density − t_n1083 | Gentianales

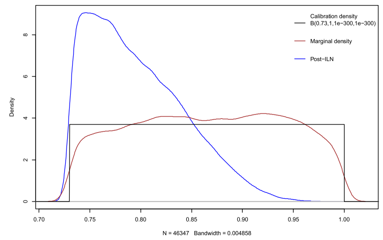

Post VS Marginal density − t_n1284 | Ginkgo−Cycas

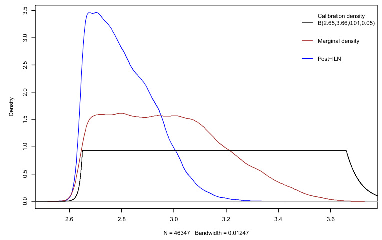

Post VS Marginal density − t_n1282 | Gnetales

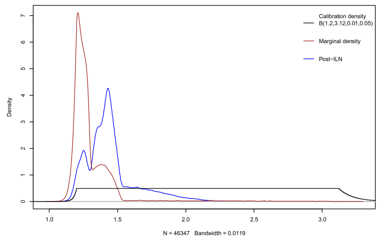

Post VS Marginal density − t_n906 | Haloragaceae

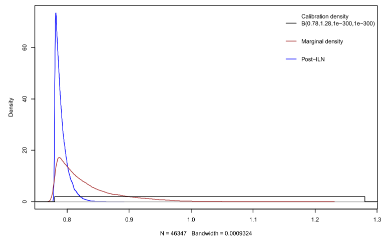

Post VS Marginal density − t_n914 | Hamamelidaceae

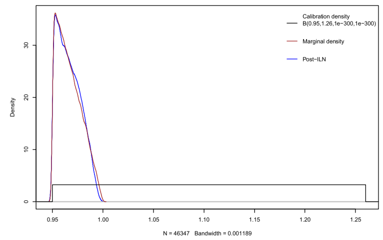

Post VS Marginal density − t_n791 | Humiriaceae

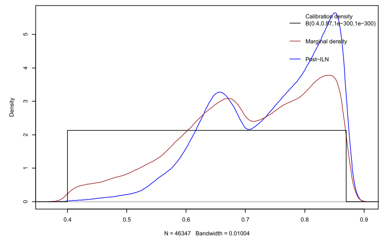

Post VS Marginal density − t_n779 | Hypericaceae

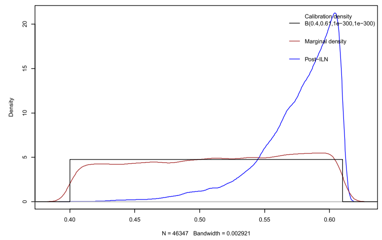

Post VS Marginal density − t_n1054 | Lamiales

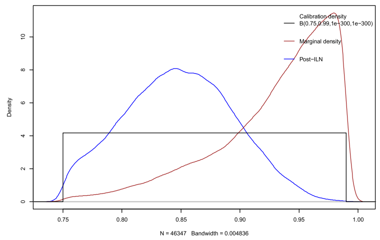

Post VS Marginal density − t_n1188 | Lardizabalaceae

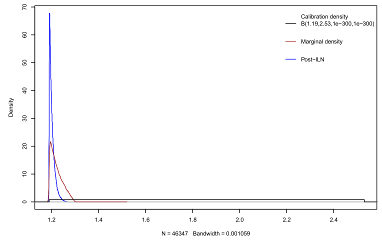

Post VS Marginal density − t_n1239 | Lauraceae

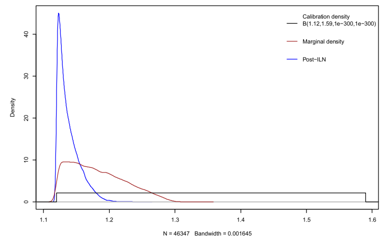

Post VS Marginal density − t_n1237 | Laurales

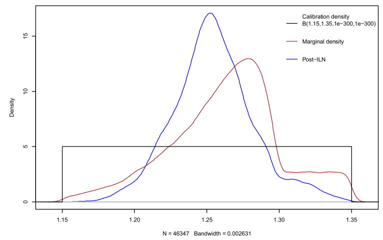

Post VS Marginal density − t_n1287 | Lycophytes

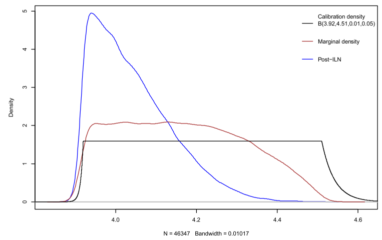

Post VS Marginal density − t_n1249 | Magnoliaceae

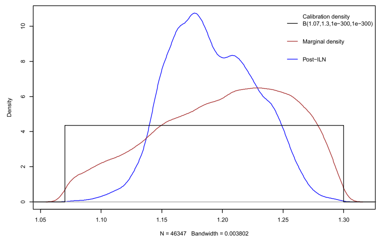

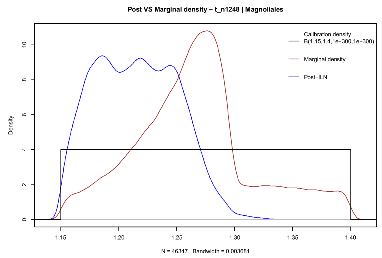
Post VS Marginal density ~ t_n1248 | Magnoliales
Calibration density
B(1.15,1.4,1e−300,1e−300)
Marginal density
Post−ILN
Density
N = 46347 Bandwidth = 0.003681

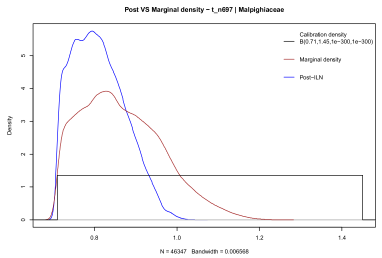
Post VS Marginal density ~ t_n697 | Malpighiaceae
Calibration density
B(0.71,1.45,1e−300,1e−300)
Marginal density
Post−ILN
Density
N = 46347 Bandwidth = 0.006568

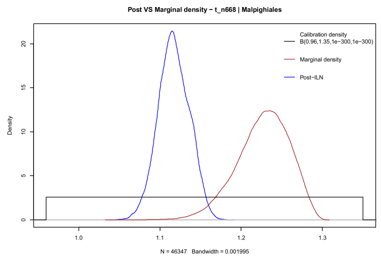
Post VS Marginal density ~ t_n668 | Malpighiales
Calibration density
B(0.96,1.35,1e−300,1e−300)
Marginal density
Post−ILN
Density
N = 46347 Bandwidth = 0.001995

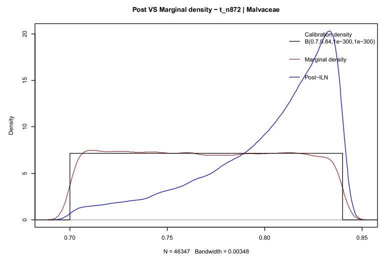
Post VS Marginal density ~ t_n872 | Malvaceae
Calibration density
Marginal density
Post−ILN
Density
N = 46347 Bandwidth = 0.00348

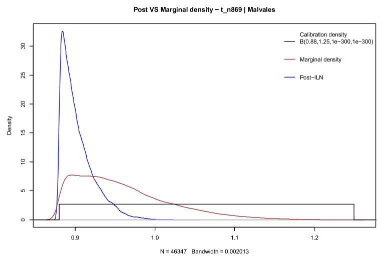
Post VS Marginal density ~ t_n869 | Malvales
Calibration density
B(0.88,1.25,1e−300,1e−300)
Marginal density
Post−ILN
Density
N = 46347 Bandwidth = 0.002013

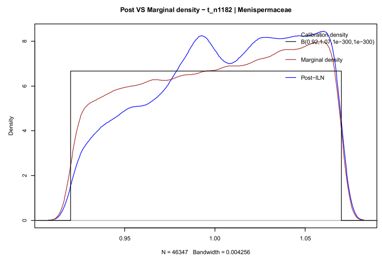
Post VS Marginal density ~ t_n1182 | Menispermaceae
Calibration density
Marginal density
Post−ILN
Density
N = 46347 Bandwidth = 0.004256

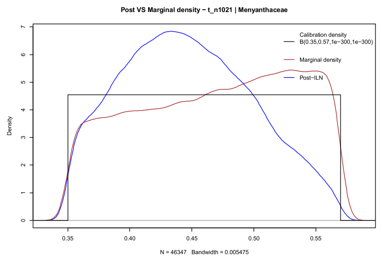
Post VS Marginal density ~ t_n1021 | Menyanthaceae
Calibration density
B(0.35,0.57,1e−300,1e−300)
Marginal density
Post−ILN
Density
N = 46347 Bandwidth = 0.005475

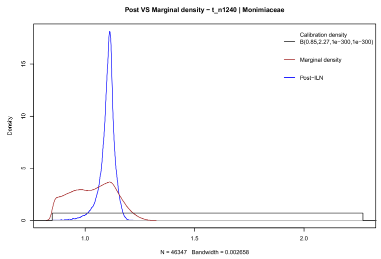
Post VS Marginal density ~ t_n1240 | Monimiaceae
Calibration density
B(0.85,2.27,1e−300,1e−300)
Marginal density
Post−ILN
Density
N = 46347 Bandwidth = 0.002658

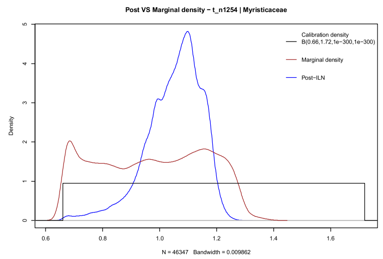
Post VS Marginal density ~ t_n1254 | Myristicaceae
Calibration density
B(0.66,1.72,1e−300,1e−300)
Marginal density
Post−ILN
Density
N = 46347 Bandwidth = 0.009862

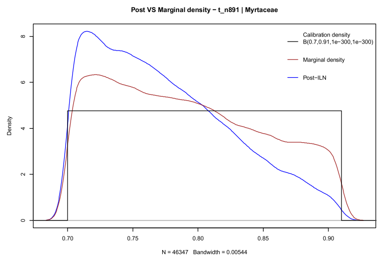
Post VS Marginal density ~ t_n891 | Myrtaceae
Calibration density
B(0.7,0.91,1e−300,1e−300)
Marginal density
Post−ILN
Density
N = 46347 Bandwidth = 0.00544

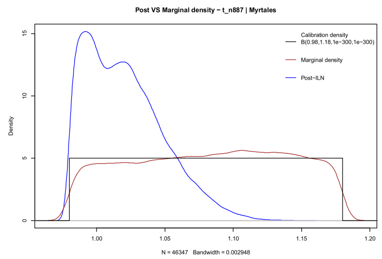
Post VS Marginal density ~ t_n887 | Myrtales
Calibration density
B(0.98,1.18,1e−300,1e−300)
Marginal density
Post−ILN
Density
N = 46347 Bandwidth = 0.002948

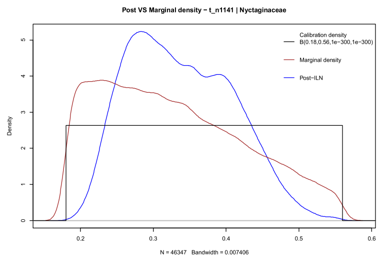
Post VS Marginal density ~ t_n1141 | Nyctaginaceae
Calibration density
B(0.18,0.56,1e−300,1e−300)
Marginal density
Post−ILN
Density
N = 46347 Bandwidth = 0.007406

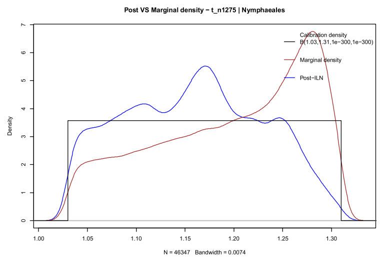
Post VS Marginal density ~ t_n1275 | Nymphaeales
Calibration density
Marginal density
Post−ILN
Density
N = 46347 Bandwidth = 0.0074

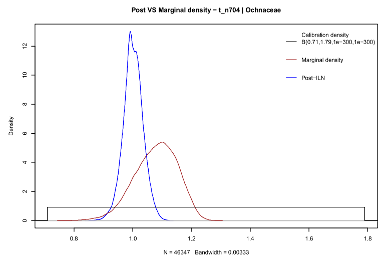
Post VS Marginal density ~ t_n704 | Ochnaceae
Calibration density
B(0.71,1.79,1e−300,1e−300)
Marginal density
Post−ILN
Density
N = 46347 Bandwidth = 0.00333

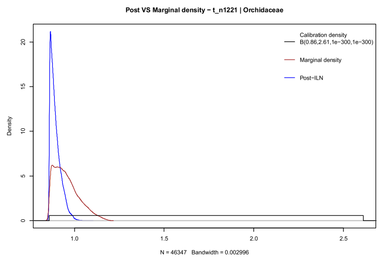
Post VS Marginal density ~ t_n1221 | Orchidaceae
Calibration density
B(0.86,2.61,1e−300,1e−300)
Marginal density
Post−ILN
Density
N = 46347 Bandwidth = 0.002996

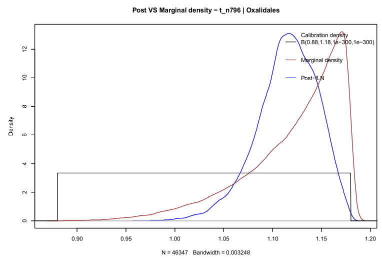
Post VS Marginal density ~ t_n796 | Oxalidales
Calibration density
Marginal density
Post−ILN
Density
N = 46347 Bandwidth = 0.003248

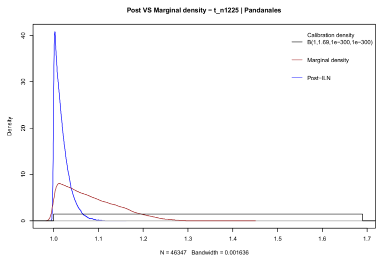
Post VS Marginal density ~ t_n1225 | Pandanales
Calibration density
B(1,1.69,1e−300,1e−300)
Marginal density
Post−ILN
Density
N = 46347 Bandwidth = 0.001636

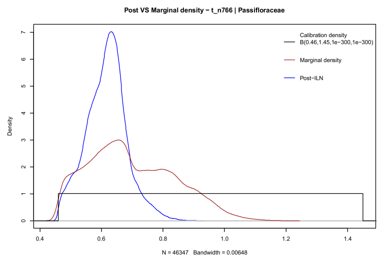
Post VS Marginal density ~ t_n766 | Passifloraceae
Calibration density
B(0.46,1.45,1e−300,1e−300)
Marginal density
Post−ILN
Density
N = 46347 Bandwidth = 0.00648

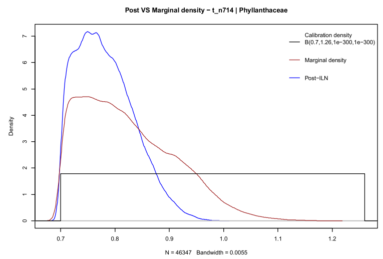
Post VS Marginal density ~ t_n714 | Phyllanthaceae
Calibration density
B(0.7,1.26,1e−300,1e−300)
Marginal density
Post−ILN
Density
N = 46347 Bandwidth = 0.0055

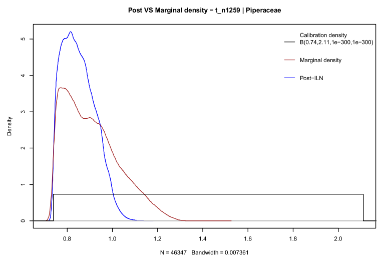
Post VS Marginal density ~ t_n1259 | Piperaceae
Calibration density
B(0.74,2.11,1e−300,1e−300)
Marginal density
Post−ILN
Density
N = 46347 Bandwidth = 0.007361

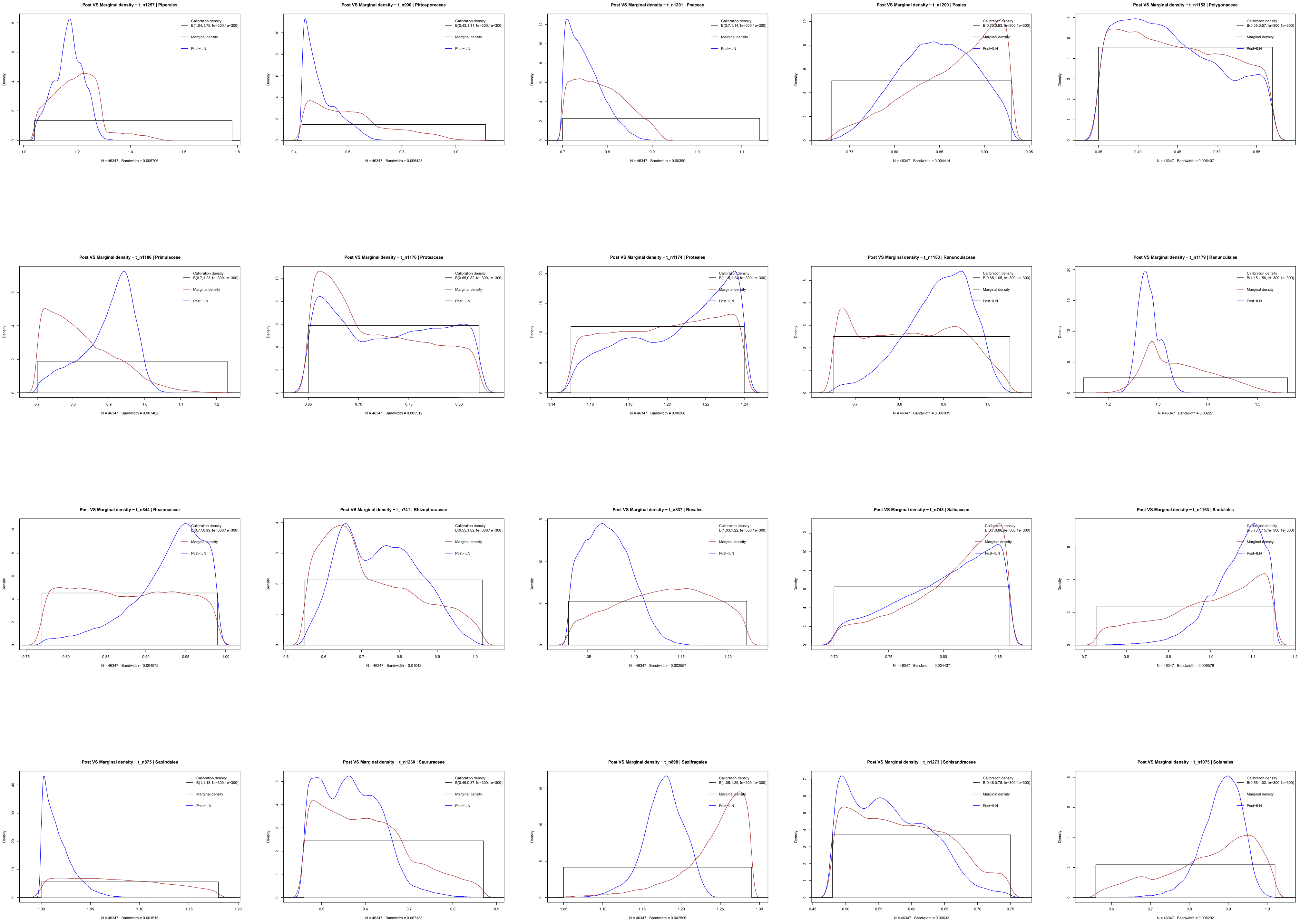
Post VS Marginal density − t_n1257 | Piperales
Calibration density B(1.04,1.78,1e-300,1e-300)
Marginal density
Post−ILN
N = 46347 Bandwidth = 0.005706
Post VS Marginal density − t_n990 | Pittosporaceae
Calibration density B(0.43,1.11,1e-300,1e-300)
N = 46347 Bandwidth = 0.006428
Post VS Marginal density − t_n1201 | Poaceae
Calibration density B(0.7,1.14,1e-300,1e-300)
N = 46347 Bandwidth = 0.00399
Post VS Marginal density − t_n1200 | Poales
N = 46347 Bandwidth = 0.004414
Post VS Marginal density − t_n1153 | Polygonaceae
Calibration density B(0.35,0.57,1e-300,1e-300)
N = 46347 Bandwidth = 0.006407
Post VS Marginal density − t_n1106 | Primulaceae
Calibration density B(0.7,1.23,1e-300,1e-300)
N = 46347 Bandwidth = 0.007462
Post VS Marginal density − t_n1176 | Proteaceae
Calibration density B(0.65,0.82,1e-300,1e-300)
N = 46347 Bandwidth = 0.005512
Post VS Marginal density − t_n1174 | Proteales
N = 46347 Bandwidth = 0.00268
Post VS Marginal density − t_n1183 | Ranunculaceae
Calibration density B(0.65,1.05,1e-300,1e-300)
N = 46347 Bandwidth = 0.007935
Post VS Marginal density − t_n1179 | Ranunculales
Calibration density B(1.15,1.56,1e-300,1e-300)
N = 46347 Bandwidth = 0.00227
Post VS Marginal density − t_n844 | Rhamnaceae
N = 46347 Bandwidth = 0.004575
Post VS Marginal density − t_n741 | Rhizophoraceae
Calibration density B(0.55,1.02,1e-300,1e-300)
N = 46347 Bandwidth = 0.01042
Post VS Marginal density − t_n837 | Rosales
Calibration density B(1.03,1.22,1e-300,1e-300)
N = 46347 Bandwidth = 0.002557
Post VS Marginal density − t_n748 | Salicaceae
N = 46347 Bandwidth = 0.004437
Post VS Marginal density − t_n1163 | Santalales
N = 46347 Bandwidth = 0.006579
Post VS Marginal density − t_n875 | Sapindales
Calibration density B(1,1.18,1e-300,1e-300)
N = 46347 Bandwidth = 0.001515
Post VS Marginal density − t_n1260 | Saururaceae
Calibration density B(0.46,0.87,1e-300,1e-300)
N = 46347 Bandwidth = 0.007138
Post VS Marginal density − t_n898 | Saxifragales
Calibration density B(1.05,1.29,1e-300,1e-300)
N = 46347 Bandwidth = 0.002596
Post VS Marginal density − t_n1273 | Schisandraceae
Calibration density B(0.48,0.75,1e-300,1e-300)
N = 46347 Bandwidth = 0.00632
Post VS Marginal density − t_n1075 | Solanales
Calibration density B(0.56,1.02,1e-300,1e-300)
N = 46347 Bandwidth = 0.005292
Density

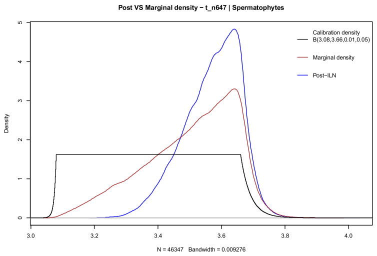
Post VS Marginal density − t_n647 | Spermatophytes
Density
Calibration density
B(3.08,3.66,0.01,0.05)
Marginal density
Post−ILN
N = 46347   Bandwidth = 0.009276

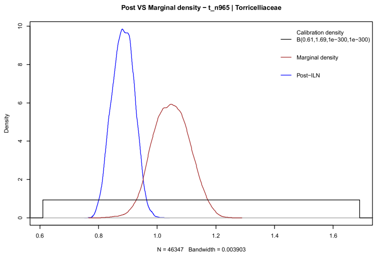
Post VS Marginal density − t_n965 | Torricelliaceae
Density
Calibration density
B(0.61,1.69,1e−300,1e−300)
Marginal density
Post−ILN
N = 46347   Bandwidth = 0.003903

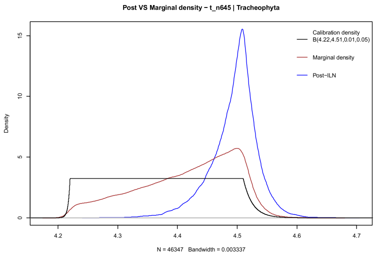
Post VS Marginal density − t_n645 | Tracheophyta
Density
Calibration density
B(4.22,4.51,0.01,0.05)
Marginal density
Post−ILN
N = 46347   Bandwidth = 0.003337

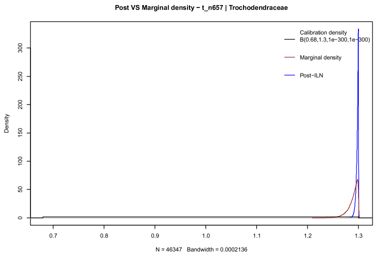
Post VS Marginal density − t_n657 | Trochodendraceae
Density
Calibration density
B(0.68,1.3,1e−300,1e−300)
Marginal density
Post−ILN
N = 46347   Bandwidth = 0.0002136

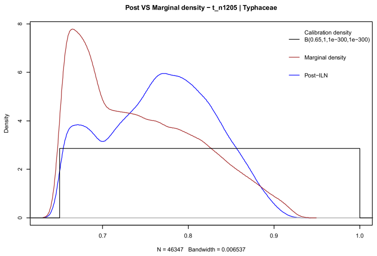
Post VS Marginal density − t_n1205 | Typhaceae
Density
Calibration density
B(0.65,1,1e−300,1e−300)
Marginal density
Post−ILN
N = 46347   Bandwidth = 0.006537

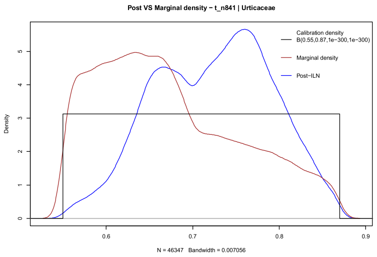
Post VS Marginal density − t_n841 | Urticaceae
Density
Calibration density
B(0.55,0.87,1e−300,1e−300)
Marginal density
Post−ILN
N = 46347   Bandwidth = 0.007056

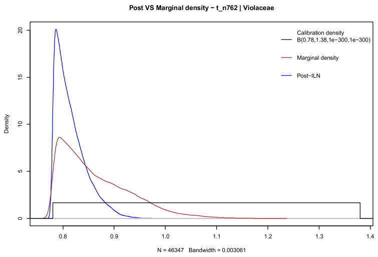
Post VS Marginal density − t_n762 | Violaceae
Density
Calibration density
B(0.78,1.38,1e−300,1e−300)
Marginal density
Post−ILN
N = 46347   Bandwidth = 0.003061

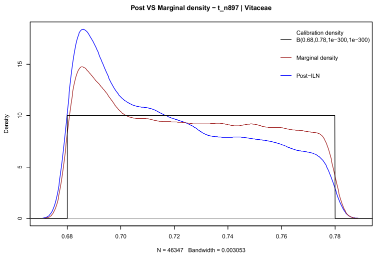
Post VS Marginal density − t_n897 | Vitaceae
Density
Calibration density
B(0.68,0.78,1e−300,1e−300)
Marginal density
Post−ILN
N = 46347   Bandwidth = 0.003053

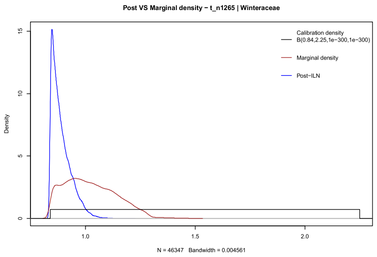
Post VS Marginal density − t_n1265 | Winteraceae
Density
Calibration density
B(0.84,2.25,1e−300,1e−300)
Marginal density
Post−ILN
N = 46347   Bandwidth = 0.004561

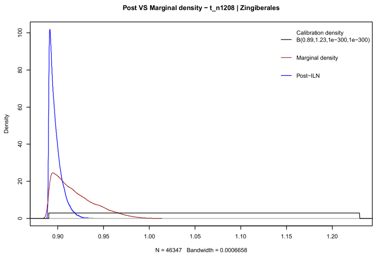
Post VS Marginal density − t_n1208 | Zingiberales
Density
Calibration density
B(0.89,1.23,1e−300,1e−300)
Marginal density
Post−ILN
N = 46347   Bandwidth = 0.0006658

Strategy 2: comparison of marginal densities (effective priors) against calibration densities (user-specified priors) and posterior densities for those nodes which age had been constrained with a uniform density with soft bounds.

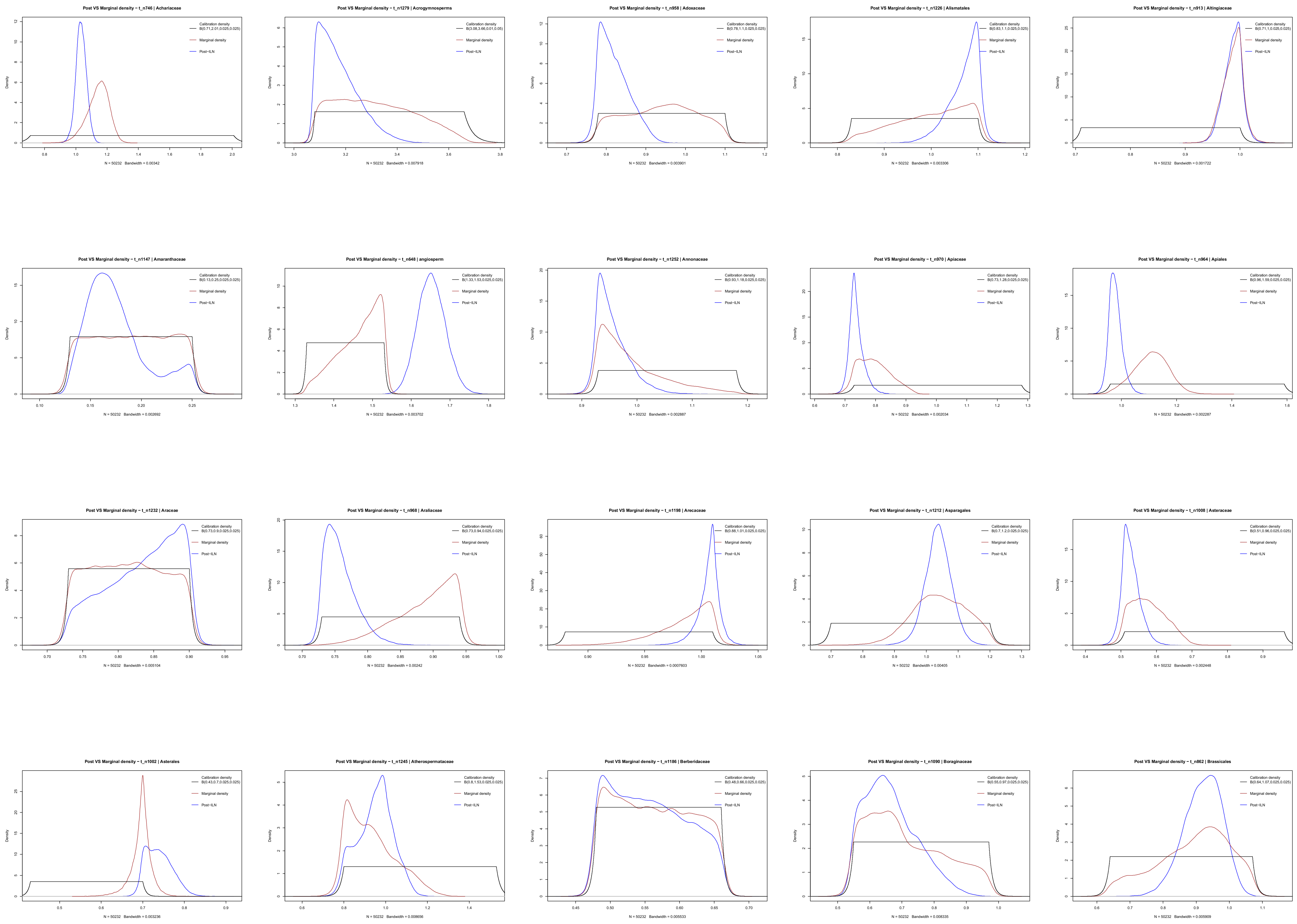

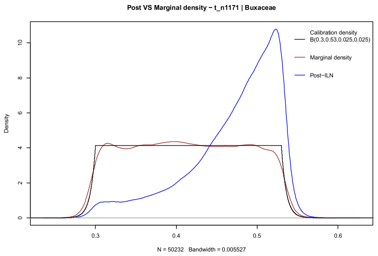
Post VS Marginal density − t_n1171 | Buxaceae
Calibration density
B(0.3,0.53,0.025,0.025)
Marginal density
Post−ILN
Density
N = 50232 Bandwidth = 0.005527

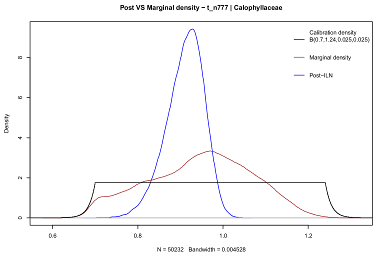
Post VS Marginal density − t_n777 | Calophyllaceae
Calibration density
B(0.7,1.24,0.025,0.025)
Marginal density
Post−ILN
Density
N = 50232 Bandwidth = 0.004528

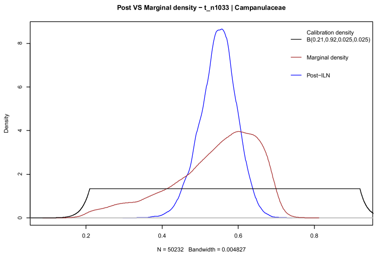
Post VS Marginal density − t_n1033 | Campanulaceae
Calibration density
B(0.21,0.92,0.025,0.025)
Marginal density
Post−ILN
Density
N = 50232 Bandwidth = 0.004827

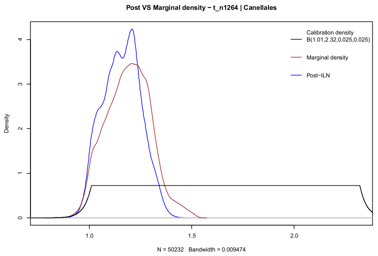
Post VS Marginal density − t_n1264 | Canellales
Calibration density
B(1.01,2.32,0.025,0.025)
Marginal density
Post−ILN
Density
N = 50232 Bandwidth = 0.009474

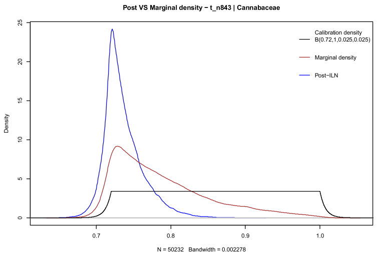
Post VS Marginal density − t_n843 | Cannabaceae
Calibration density
B(0.72,1,0.025,0.025)
Marginal density
Post−ILN
Density
N = 50232 Bandwidth = 0.002278

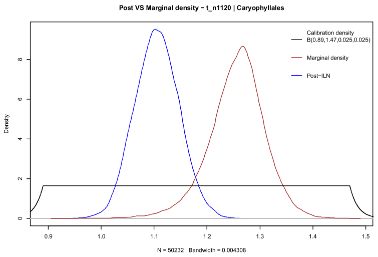
Post VS Marginal density − t_n1120 | Caryophyllales
Calibration density
B(0.89,1.47,0.025,0.025)
Marginal density
Post−ILN
Density
N = 50232 Bandwidth = 0.004308

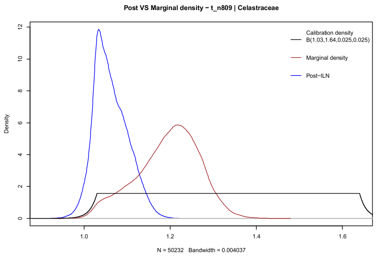
Post VS Marginal density − t_n809 | Celastraceae
Calibration density
B(1.03,1.64,0.025,0.025)
Marginal density
Post−ILN
Density
N = 50232 Bandwidth = 0.004037

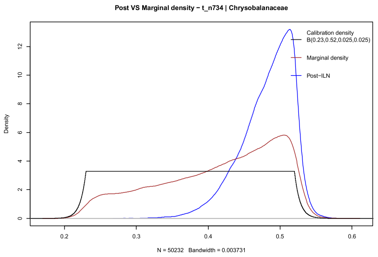
Post VS Marginal density − t_n734 | Chrysobalanaceae
Calibration density
B(0.23,0.52,0.025,0.025)
Marginal density
Post−ILN
Density
N = 50232 Bandwidth = 0.003731

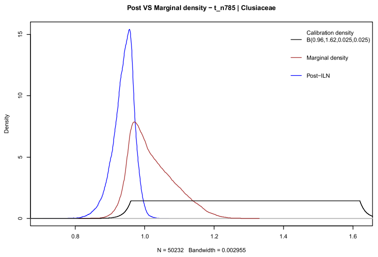
Post VS Marginal density − t_n785 | Clusiaceae
Calibration density
B(0.96,1.62,0.025,0.025)
Marginal density
Post−ILN
Density
N = 50232 Bandwidth = 0.002955

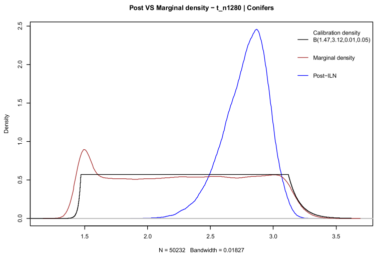
Post VS Marginal density − t_n1280 | Conifers
Calibration density
B(1.47,3.12,0.01,0.05)
Marginal density
Post−ILN
Density
N = 50232 Bandwidth = 0.01827

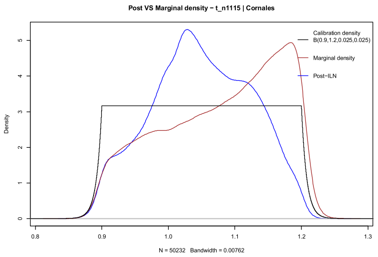
Post VS Marginal density − t_n1115 | Cornales
Calibration density
B(0.9,1.2,0.025,0.025)
Marginal density
Post−ILN
Density
N = 50232 Bandwidth = 0.00762

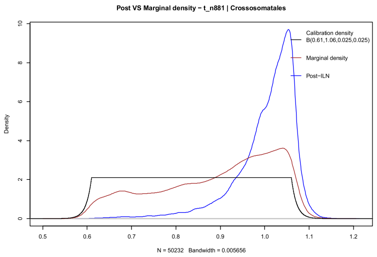
Post VS Marginal density − t_n881 | Crossosomatales
Calibration density
B(0.61,1.06,0.025,0.025)
Marginal density
Post−ILN
Density
N = 50232 Bandwidth = 0.005656

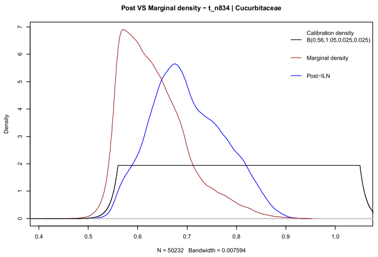
Post VS Marginal density − t_n834 | Cucurbitaceae
Calibration density
B(0.56,1.05,0.025,0.025)
Marginal density
Post−ILN
Density
N = 50232 Bandwidth = 0.007594

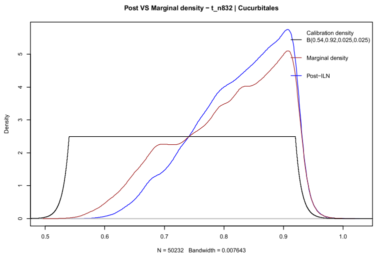
Post VS Marginal density − t_n832 | Cucurbitales
Calibration density
B(0.54,0.92,0.025,0.025)
Marginal density
Post−ILN
Density
N = 50232 Bandwidth = 0.007643

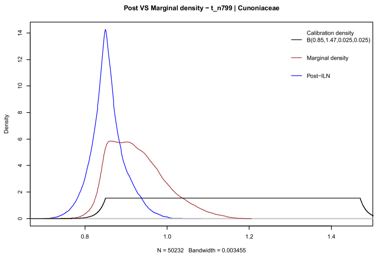
Post VS Marginal density − t_n799 | Cunoniaceae
Calibration density
B(0.85,1.47,0.025,0.025)
Marginal density
Post−ILN
Density
N = 50232 Bandwidth = 0.003455

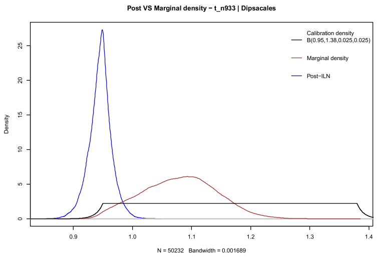
Post VS Marginal density − t_n933 | Dipsacales
Calibration density
B(0.95,1.38,0.025,0.025)
Marginal density
Post−ILN
Density
N = 50232 Bandwidth = 0.001689

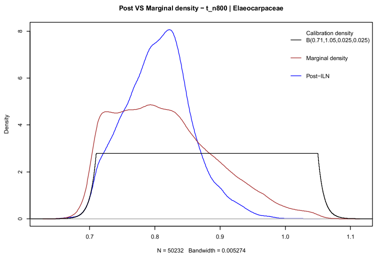
Post VS Marginal density − t_n800 | Elaeocarpaceae
Calibration density
B(0.71,1.05,0.025,0.025)
Marginal density
Post−ILN
Density
N = 50232 Bandwidth = 0.005274

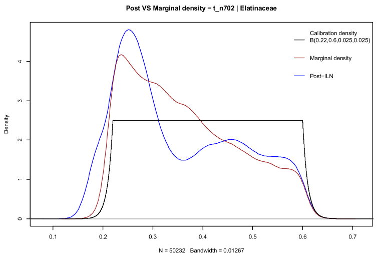
Post VS Marginal density − t_n702 | Elatinaceae
Calibration density
B(0.22,0.6,0.025,0.025)
Marginal density
Post−ILN
Density
N = 50232 Bandwidth = 0.01267

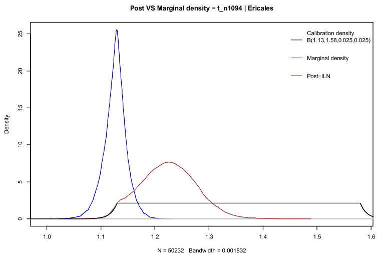
Post VS Marginal density − t_n1094 | Ericales
Calibration density
B(1.13,1.58,0.025,0.025)
Marginal density
Post−ILN
Density
N = 50232 Bandwidth = 0.001832

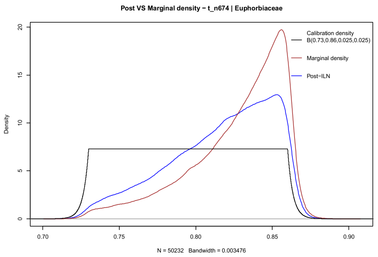
Post VS Marginal density − t_n674 | Euphorbiaceae
Calibration density
B(0.73,0.86,0.025,0.025)
Marginal density
Post−ILN
Density
N = 50232 Bandwidth = 0.003476

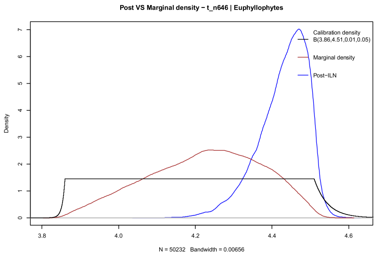
Post VS Marginal density − t_n646 | Euphyllophytes
Calibration density
B(3.86,4.51,0.01,0.05)
Marginal density
Post−ILN
Density
N = 50232 Bandwidth = 0.00656

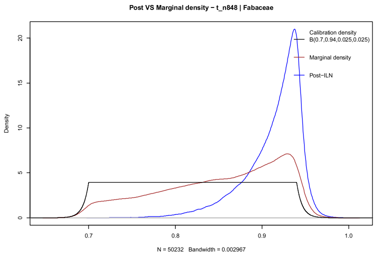
Post VS Marginal density − t_n848 | Fabaceae
Calibration density
B(0.7,0.94,0.025,0.025)
Marginal density
Post−ILN
Density
N = 50232 Bandwidth = 0.002967

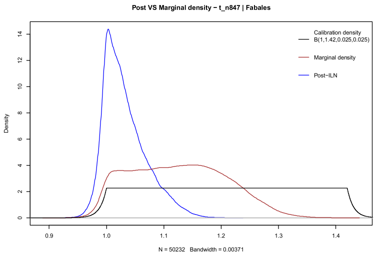
Post VS Marginal density − t_n847 | Fabales
Calibration density
B(1,1.42,0.025,0.025)
Marginal density
Post−ILN
Density
N = 50232 Bandwidth = 0.00371

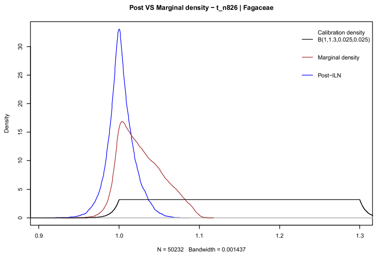
Post VS Marginal density − t_n826 | Fagaceae
Calibration density
B(1,1.3,0.025,0.025)
Marginal density
Post−ILN
Density
N = 50232 Bandwidth = 0.001437

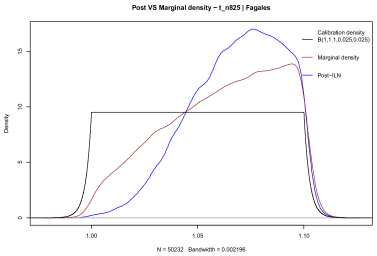
Post VS Marginal density − t_n825 | Fagales
Calibration density
B(1,1.1,0.025,0.025)
Marginal density
Post−ILN
Density
N = 50232 Bandwidth = 0.002196

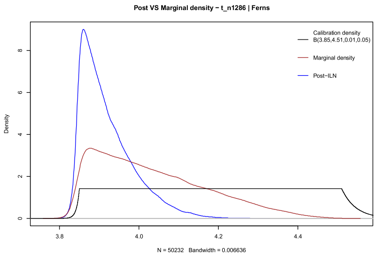
Post VS Marginal density − t_n1286 | Ferns
Calibration density
B(3.85,4.51,0.01,0.05)
Marginal density
Post−ILN
Density
N = 50232 Bandwidth = 0.006636

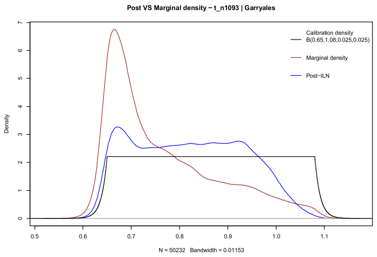
Post VS Marginal density − t_n1093 | Garryales
Calibration density
B(0.65,1.08,0.025,0.025)
Marginal density
Post−ILN
Density
N = 50232 Bandwidth = 0.01153

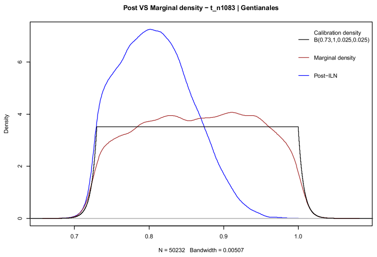
Post VS Marginal density − t_n1083 | Gentianales
Calibration density
B(0.73,1,0.025,0.025)
Marginal density
Post−ILN
Density
N = 50232 Bandwidth = 0.00507

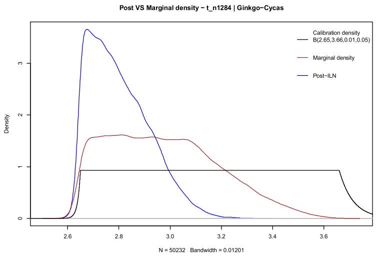
Post VS Marginal density − t_n1284 | Ginkgo−Cycas
Calibration density
B(2.65,3.66,0.01,0.05)
Marginal density
Post−ILN
Density
N = 50232 Bandwidth = 0.01201

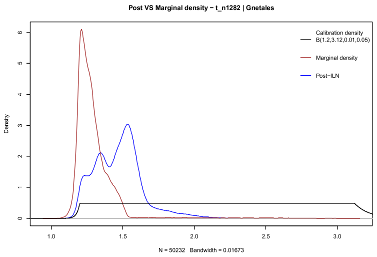
Post VS Marginal density − t_n1282 | Gnetales
Calibration density
B(1.2,3.12,0.01,0.05)
Marginal density
Post−ILN
Density
N = 50232 Bandwidth = 0.01673

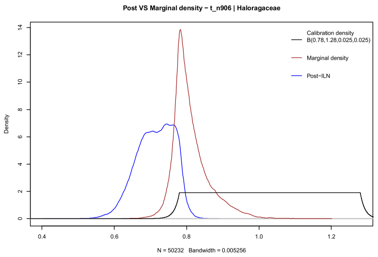
Post VS Marginal density − t_n906 | Haloragaceae
Calibration density
B(0.78,1.28,0.025,0.025)
Marginal density
Post−ILN
Density
N = 50232 Bandwidth = 0.005256

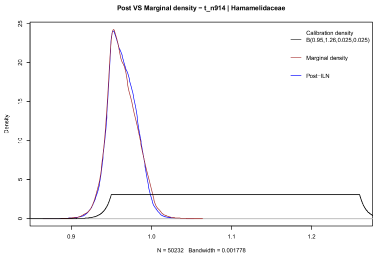
Post VS Marginal density − t_n914 | Hamamelidaceae
Calibration density
B(0.95,1.26,0.025,0.025)
Marginal density
Post−ILN
Density
N = 50232 Bandwidth = 0.001778

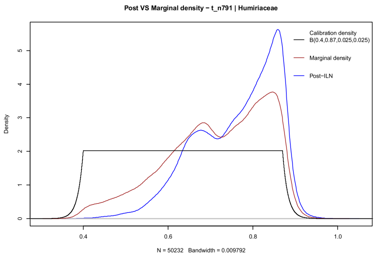
Post VS Marginal density − t_n791 | Humiriaceae
Calibration density
B(0.4,0.87,0.025,0.025)
Marginal density
Post−ILN
Density
N = 50232 Bandwidth = 0.009792

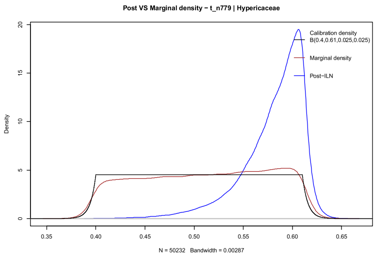
Post VS Marginal density − t_n779 | Hypericaceae
Calibration density
B(0.4,0.61,0.025,0.025)
Marginal density
Post−ILN
Density
N = 50232 Bandwidth = 0.00287

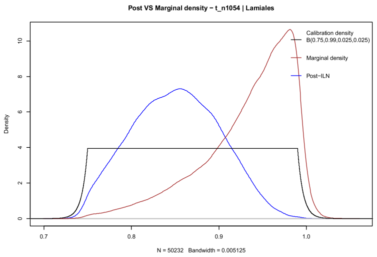
Post VS Marginal density − t_n1054 | Lamiales
Calibration density
B(0.75,0.99,0.025,0.025)
Marginal density
Post−ILN
Density
N = 50232 Bandwidth = 0.005125

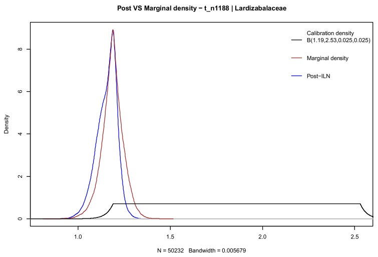
Post VS Marginal density − t_n1188 | Lardizabalaceae
Calibration density
B(1.19,2.53,0.025,0.025)
Marginal density
Post−ILN
Density
N = 50232 Bandwidth = 0.005679

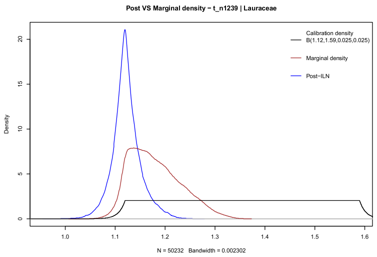
Post VS Marginal density − t_n1239 | Lauraceae
Calibration density
B(1.12,1.59,0.025,0.025)
Marginal density
Post−ILN
Density
N = 50232 Bandwidth = 0.002302

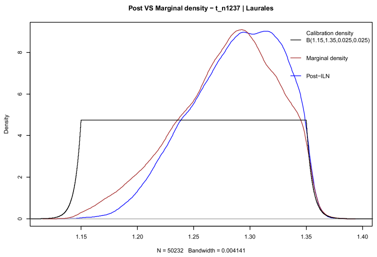
Post VS Marginal density − t_n1237 | Laurales
Calibration density
B(1.15,1.35,0.025,0.025)
Marginal density
Post−ILN
Density
N = 50232 Bandwidth = 0.004141

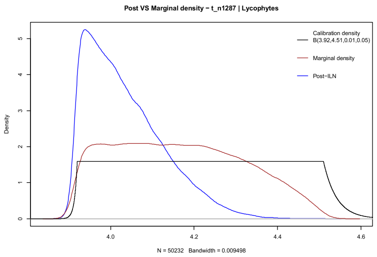
Post VS Marginal density − t_n1287 | Lycophytes
Calibration density
B(3.92,4.51,0.01,0.05)
Marginal density
Post−ILN
Density
N = 50232 Bandwidth = 0.009498

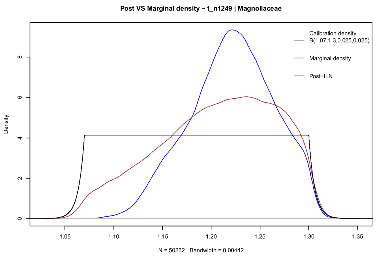
Post VS Marginal density − t_n1249 | Magnoliaceae
Calibration density
B(1.07,1.3,0.025,0.025)
Marginal density
Post−ILN
Density
N = 50232 Bandwidth = 0.00442

Post VS Marginal density ~ t_n1248 | Magnoliales

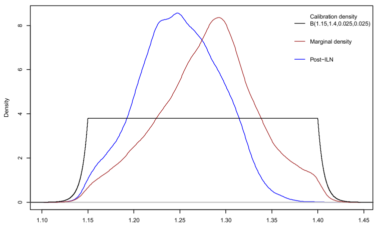

N = 50232 Bandwidth = 0.004566

Post VS Marginal density ~ t_n697 | Malpighiaceae

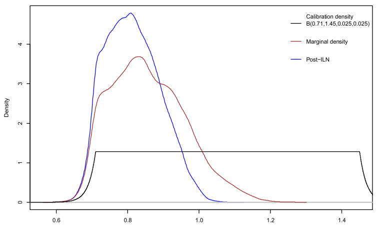

N = 50232 Bandwidth = 0.007777

Post VS Marginal density ~ t_n668 | Malpighiales

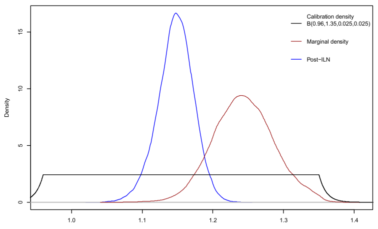

N = 50232 Bandwidth = 0.00251

Post VS Marginal density ~ t_n872 | Malvaceae

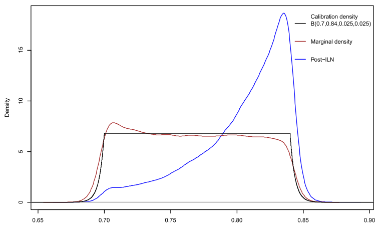

N = 50232 Bandwidth = 0.00353

Post VS Marginal density ~ t_n869 | Malvales

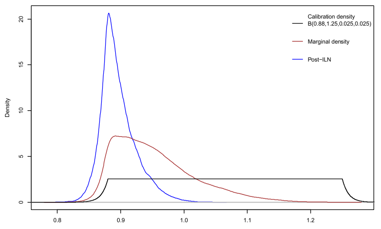

N = 50232 Bandwidth = 0.00251

Post VS Marginal density ~ t_n1182 | Menispermaceae

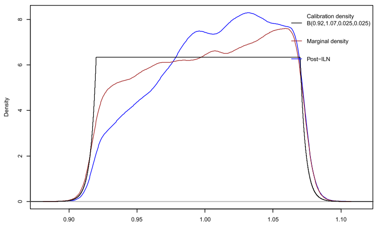

N = 50232 Bandwidth = 0.004315

Post VS Marginal density ~ t_n1021 | Menyanthaceae

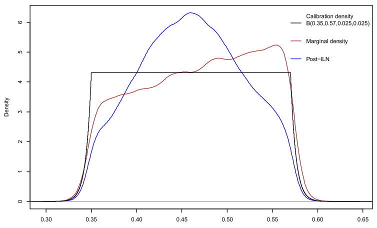

N = 50232 Bandwidth = 0.005857

Post VS Marginal density ~ t_n1240 | Monimiaceae

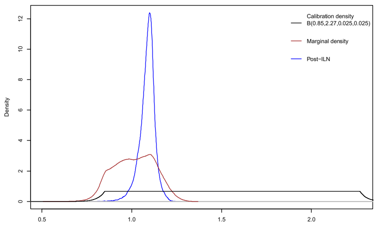

N = 50232 Bandwidth = 0.003653

Post VS Marginal density ~ t_n1254 | Myristicaceae

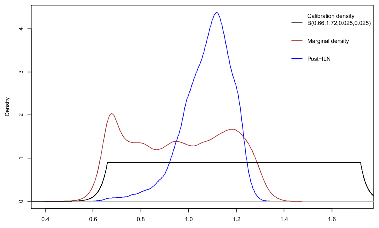

N = 50232 Bandwidth = 0.01027

Post VS Marginal density ~ t_n891 | Myrtaceae

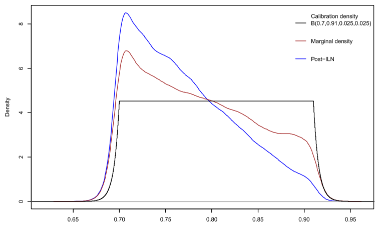

N = 50232 Bandwidth = 0.005727

Post VS Marginal density ~ t_n887 | Myrtales

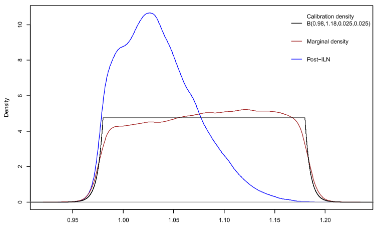

N = 50232 Bandwidth = 0.003894

Post VS Marginal density ~ t_n1141 | Nyctaginaceae

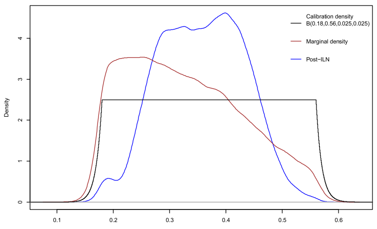

N = 50232 Bandwidth = 0.007734

Post VS Marginal density ~ t_n1275 | Nymphaeales

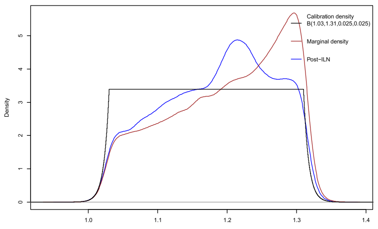

N = 50232 Bandwidth = 0.008192

Post VS Marginal density ~ t_n704 | Ochnaceae

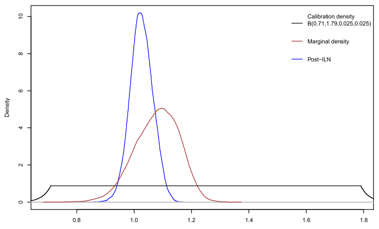

N = 50232 Bandwidth = 0.004056

Post VS Marginal density ~ t_n1221 | Orchidaceae

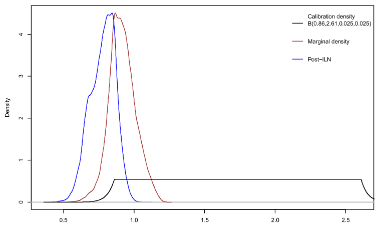

N = 50232 Bandwidth = 0.009158

Post VS Marginal density ~ t_n796 | Oxalidales

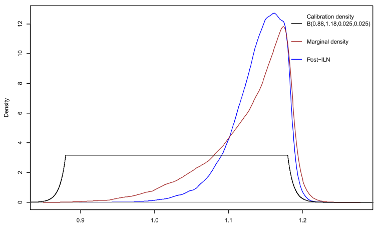

N = 50232 Bandwidth = 0.003491

Post VS Marginal density ~ t_n1225 | Pandanales

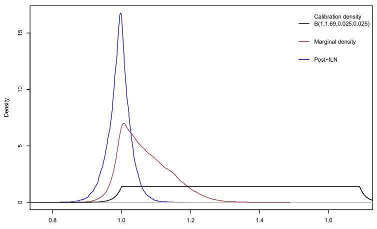

N = 50232 Bandwidth = 0.002827

Post VS Marginal density ~ t_n766 | Passifloraceae

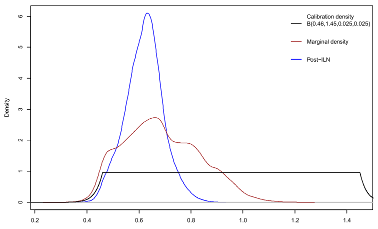

N = 50232 Bandwidth = 0.007073

Post VS Marginal density ~ t_n714 | Phyllanthaceae

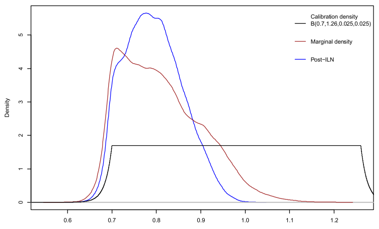

N = 50232 Bandwidth = 0.006595

Post VS Marginal density ~ t_n1259 | Piperaceae

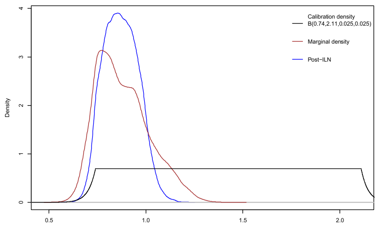

N = 50232 Bandwidth = 0.009156

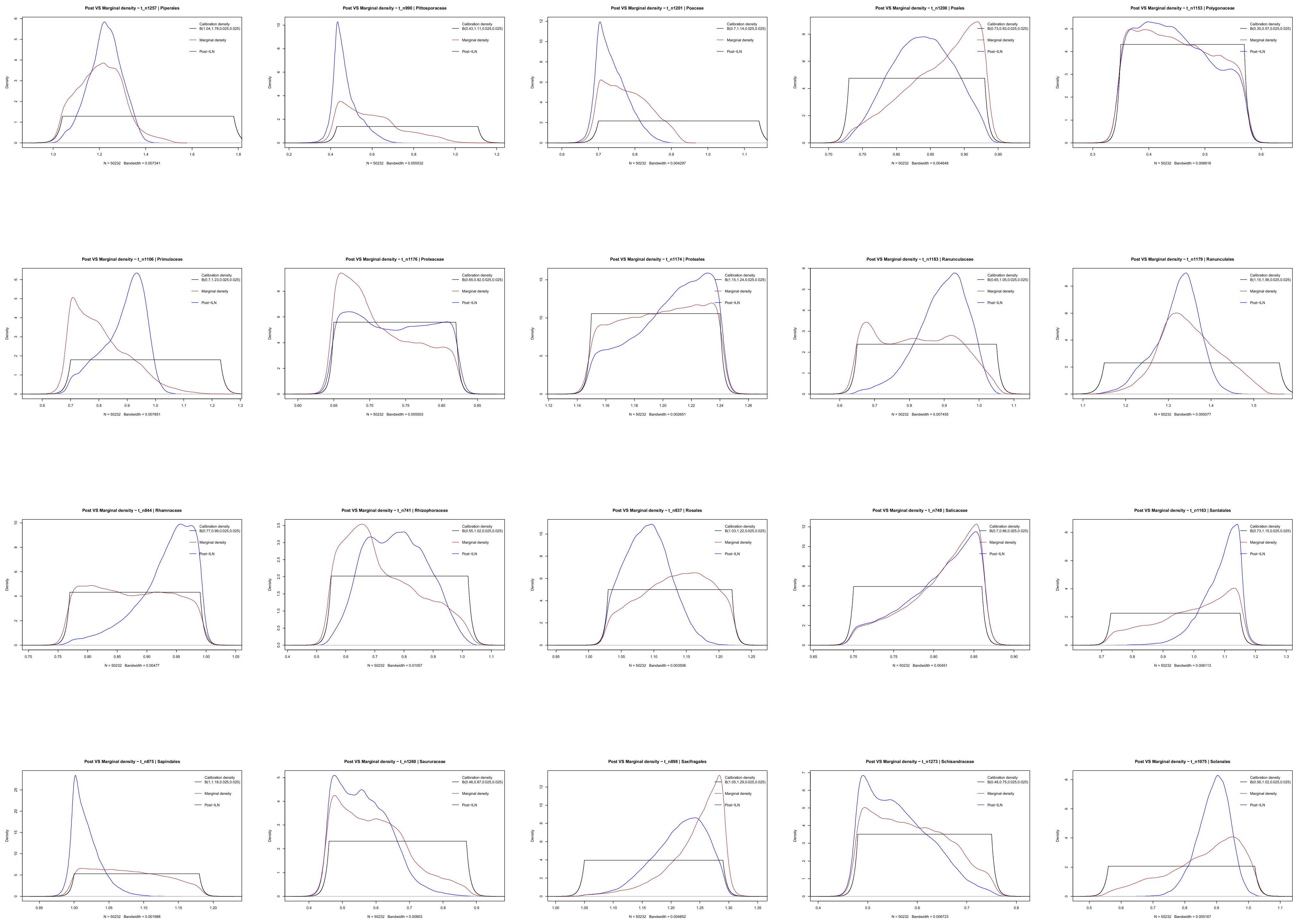

Post VS Marginal density – t_n1257 | Piperales
Calibration density B(1.04,1.78,0.025,0.025)
Marginal density
Post−ILN
N = 50232 Bandwidth = 0.007341
Post VS Marginal density – t_n990 | Pittosporaceae
Calibration density B(0.43,1.11,0.025,0.025)
N = 50232 Bandwidth = 0.005532
Post VS Marginal density – t_n1201 | Poaceae
Calibration density B(0.7,1.14,0.025,0.025)
N = 50232 Bandwidth = 0.004297
Post VS Marginal density – t_n1200 | Poales
Calibration density B(0.73,0.93,0.025,0.025)
N = 50232 Bandwidth = 0.004648
Post VS Marginal density – t_n1153 | Polygonaceae
Calibration density B(0.35,0.57,0.025,0.025)
N = 50232 Bandwidth = 0.006616
Post VS Marginal density – t_n1106 | Primulaceae
Calibration density B(0.7,1.23,0.025,0.025)
N = 50232 Bandwidth = 0.007851
Post VS Marginal density – t_n1176 | Proteaceae
Calibration density B(0.65,0.82,0.025,0.025)
N = 50232 Bandwidth = 0.005503
Post VS Marginal density – t_n1174 | Proteales
Calibration density B(1.15,1.24,0.025,0.025)
N = 50232 Bandwidth = 0.002651
Post VS Marginal density – t_n1183 | Ranunculaceae
Calibration density B(0.65,1.05,0.025,0.025)
N = 50232 Bandwidth = 0.007455
Post VS Marginal density – t_n1179 | Ranunculales
Calibration density B(1.15,1.56,0.025,0.025)
N = 50232 Bandwidth = 0.005077
Post VS Marginal density – t_n844 | Rhamnaceae
Calibration density B(0.77,0.99,0.025,0.025)
N = 50232 Bandwidth = 0.00477
Post VS Marginal density – t_n741 | Rhizophoraceae
Calibration density B(0.55,1.02,0.025,0.025)
N = 50232 Bandwidth = 0.01057
Post VS Marginal density – t_n837 | Rosales
Calibration density B(1.03,1.22,0.025,0.025)
N = 50232 Bandwidth = 0.003506
Post VS Marginal density – t_n748 | Salicaceae
Calibration density B(0.7,0.86,0.025,0.025)
N = 50232 Bandwidth = 0.00451
Post VS Marginal density – t_n1163 | Santalales
Calibration density B(0.73,1.15,0.025,0.025)
N = 50232 Bandwidth = 0.006113
Post VS Marginal density – t_n875 | Sapindales
Calibration density B(1,1.18,0.025,0.025)
N = 50232 Bandwidth = 0.001988
Post VS Marginal density – t_n1260 | Saururaceae
Calibration density B(0.46,0.87,0.025,0.025)
N = 50232 Bandwidth = 0.00803
Post VS Marginal density – t_n898 | Saxifragales
Calibration density B(1.05,1.29,0.025,0.025)
N = 50232 Bandwidth = 0.004852
Post VS Marginal density – t_n1273 | Schisandraceae
Calibration density B(0.48,0.75,0.025,0.025)
N = 50232 Bandwidth = 0.006723
Post VS Marginal density – t_n1075 | Solanales
Calibration density B(0.56,1.02,0.025,0.025)
N = 50232 Bandwidth = 0.005167

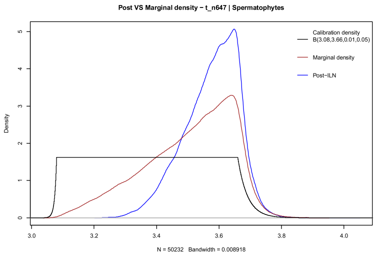
Post VS Marginal density − t_n647 | Spermatophytes
Calibration density
B(3.08,3.66,0.01,0.05)
Marginal density
Post−ILN
Density
N = 50232   Bandwidth = 0.008918

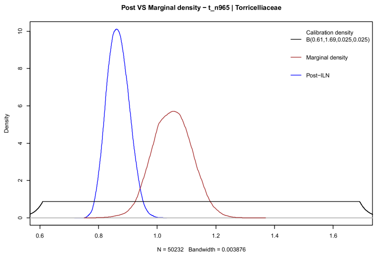
Post VS Marginal density − t_n965 | Torricelliaceae
Calibration density
B(0.61,1.69,0.025,0.025)
Marginal density
Post−ILN
Density
N = 50232   Bandwidth = 0.003876

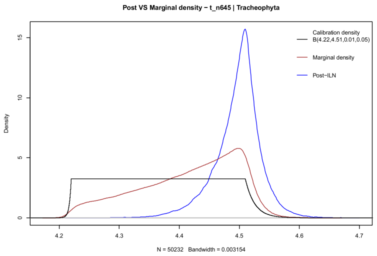
Post VS Marginal density − t_n645 | Tracheophyta
Calibration density
B(4.22,4.51,0.01,0.05)
Marginal density
Post−ILN
Density
N = 50232   Bandwidth = 0.003154

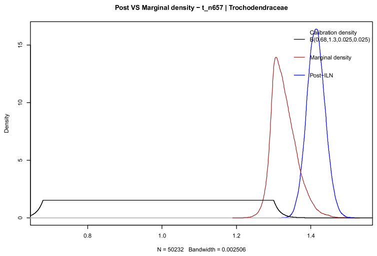
Post VS Marginal density − t_n657 | Trochodendraceae
Calibration density
B(0.68,1.3,0.025,0.025)
Marginal density
Post−ILN
Density
N = 50232   Bandwidth = 0.002506

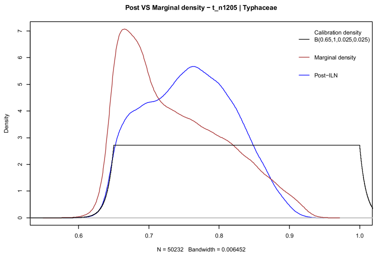
Post VS Marginal density − t_n1205 | Typhaceae
Calibration density
B(0.65,1,0.025,0.025)
Marginal density
Post−ILN
Density
N = 50232   Bandwidth = 0.006452

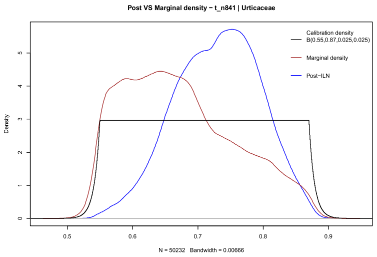
Post VS Marginal density − t_n841 | Urticaceae
Calibration density
B(0.55,0.87,0.025,0.025)
Marginal density
Post−ILN
Density
N = 50232   Bandwidth = 0.00666

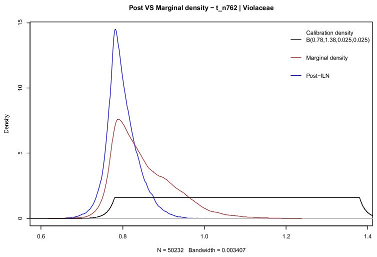
Post VS Marginal density − t_n762 | Violaceae
Calibration density
B(0.78,1.38,0.025,0.025)
Marginal density
Post−ILN
Density
N = 50232   Bandwidth = 0.003407

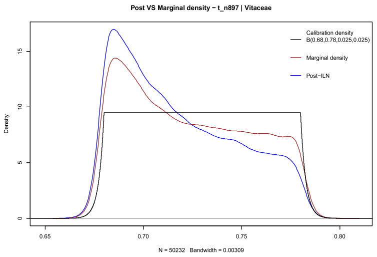
Post VS Marginal density − t_n897 | Vitaceae
Calibration density
B(0.68,0.78,0.025,0.025)
Marginal density
Post−ILN
Density
N = 50232   Bandwidth = 0.00309

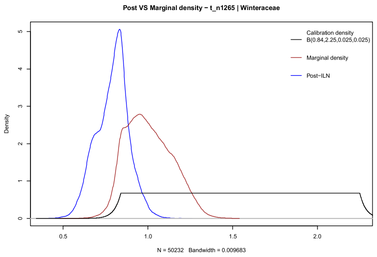
Post VS Marginal density − t_n1265 | Winteraceae
Calibration density
B(0.84,2.25,0.025,0.025)
Marginal density
Post−ILN
Density
N = 50232   Bandwidth = 0.009683

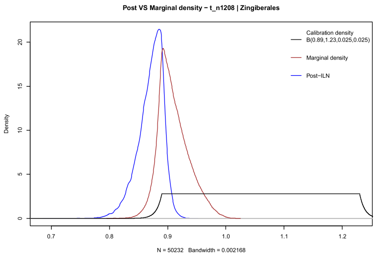
Post VS Marginal density − t_n1208 | Zingiberales
Calibration density
B(0.89,1.23,0.025,0.025)
Marginal density
Post−ILN
Density
N = 50232   Bandwidth = 0.002168

Strategy 3: comparison of marginal densities (effective priors) against calibration densities (user-specified priors) and posterior densities for those nodes which age had been constrained with a skew-T distribution.

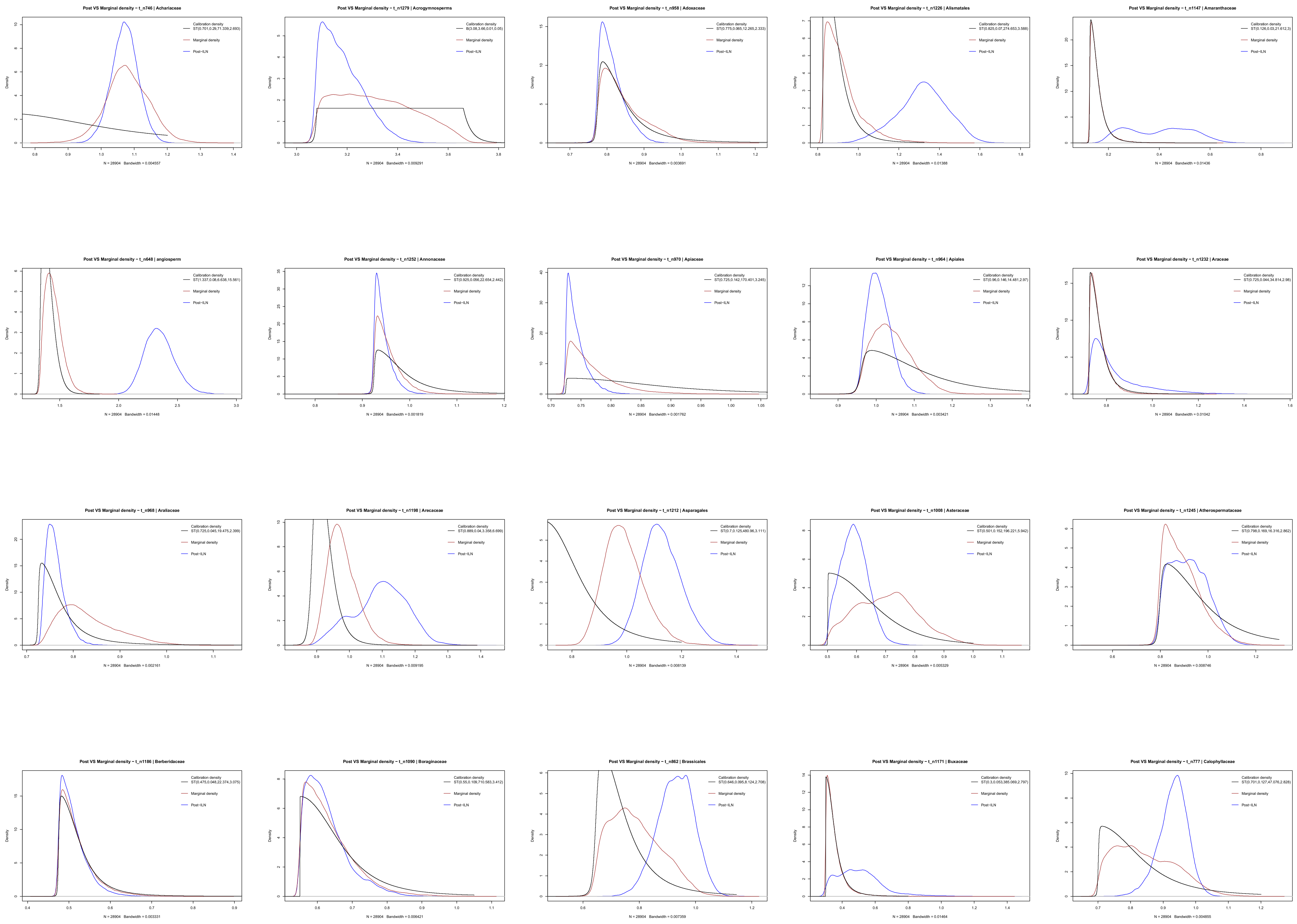

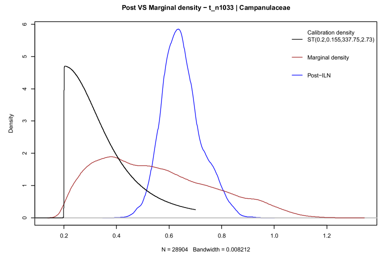
Post VS Marginal density – t_n1033 | Campanulaceae
Calibration density
ST(0.2,0.155,337.75,2.73)
Marginal density
Post−ILN
Density
N = 28904 Bandwidth = 0.008212

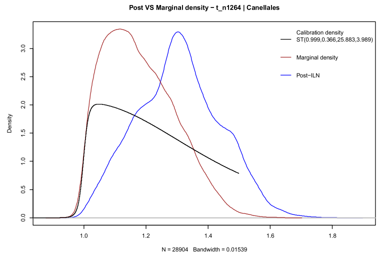
Post VS Marginal density – t_n1264 | Canellales
Calibration density
ST(0.999,0.366,25.883,3.989)
Marginal density
Post−ILN
Density
N = 28904 Bandwidth = 0.01539

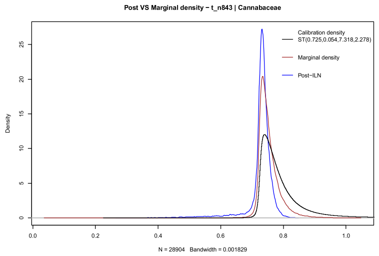
Post VS Marginal density – t_n843 | Cannabaceae
Calibration density
ST(0.725,0.054,7.318,2.278)
Marginal density
Post−ILN
Density
N = 28904 Bandwidth = 0.001829

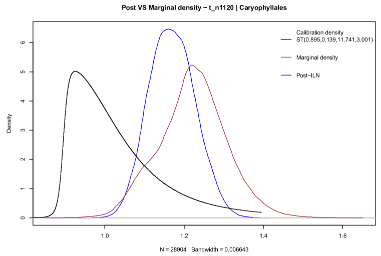
Post VS Marginal density – t_n1120 | Caryophyllales
Calibration density
ST(0.895,0.139,11.741,3.001)
Marginal density
Post−ILN
Density
N = 28904 Bandwidth = 0.006643

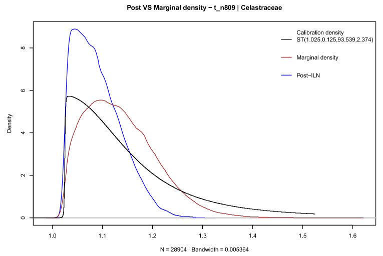
Post VS Marginal density – t_n809 | Celastraceae
Calibration density
ST(1.025,0.125,93.539,2.374)
Marginal density
Post−ILN
Density
N = 28904 Bandwidth = 0.005364

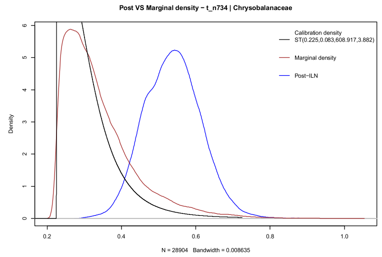
Post VS Marginal density – t_n734 | Chrysobalanaceae
Calibration density
ST(0.225,0.083,608.917,3.882)
Marginal density
Post−ILN
Density
N = 28904 Bandwidth = 0.008635

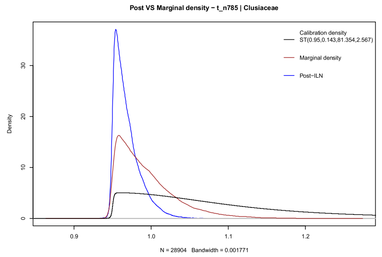
Post VS Marginal density – t_n785 | Clusiaceae
Calibration density
ST(0.95,0.143,81.354,2.567)
Marginal density
Post−ILN
Density
N = 28904 Bandwidth = 0.001771

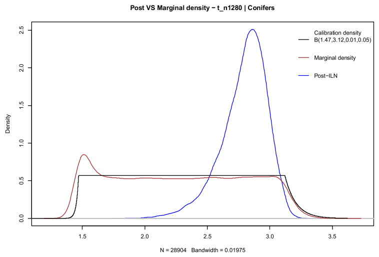
Post VS Marginal density – t_n1280 | Conifers
Calibration density
B(1.47,3.12,0.01,0.05)
Marginal density
Post−ILN
Density
N = 28904 Bandwidth = 0.01975

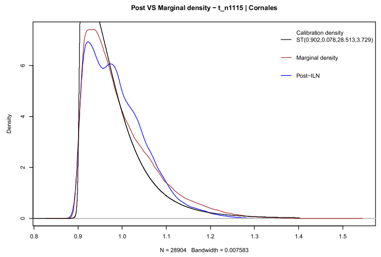
Post VS Marginal density – t_n1115 | Cornales
Calibration density
ST(0.902,0.078,28.513,3.729)
Marginal density
Post−ILN
Density
N = 28904 Bandwidth = 0.007583

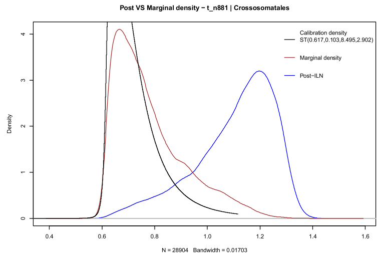
Post VS Marginal density – t_n881 | Crossosomatales
Calibration density
ST(0.617,0.103,8.495,2.902)
Marginal density
Post−ILN
Density
N = 28904 Bandwidth = 0.01703

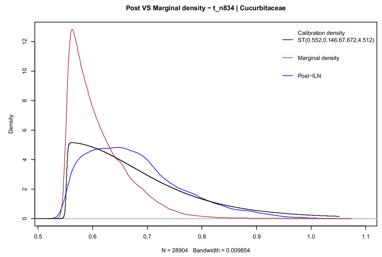
Post VS Marginal density – t_n834 | Cucurbitaceae
Calibration density
ST(0.552,0.146,67.672,4.512)
Marginal density
Post−ILN
Density
N = 28904 Bandwidth = 0.009854

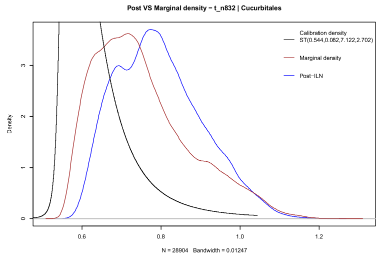
Post VS Marginal density – t_n832 | Cucurbitales
Calibration density
ST(0.544,0.082,7.122,2.702)
Marginal density
Post−ILN
Density
N = 28904 Bandwidth = 0.01247

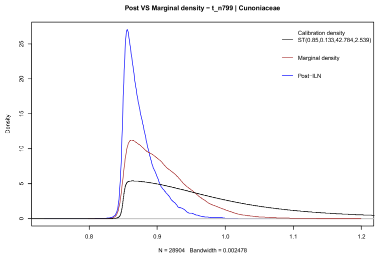
Post VS Marginal density – t_n799 | Cunoniaceae
Calibration density
ST(0.85,0.133,42.784,2.539)
Marginal density
Post−ILN
Density
N = 28904 Bandwidth = 0.002478

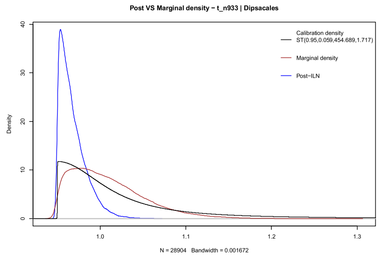
Post VS Marginal density – t_n933 | Dipsacales
Calibration density
ST(0.95,0.059,454.689,1.717)
Marginal density
Post−ILN
Density
N = 28904 Bandwidth = 0.001672

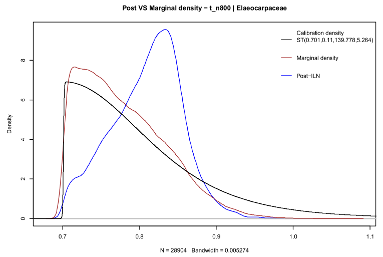
Post VS Marginal density – t_n800 | Elaeocarpaceae
Calibration density
ST(0.701,0.11,139.778,5.264)
Marginal density
Post−ILN
Density
N = 28904 Bandwidth = 0.005274

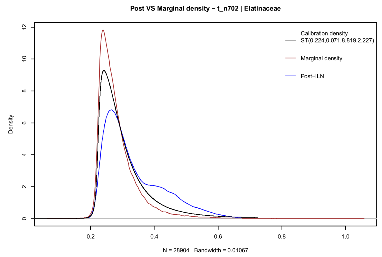
Post VS Marginal density – t_n702 | Elatinaceae
Calibration density
ST(0.224,0.071,8.819,2.227)
Marginal density
Post−ILN
Density
N = 28904 Bandwidth = 0.01067

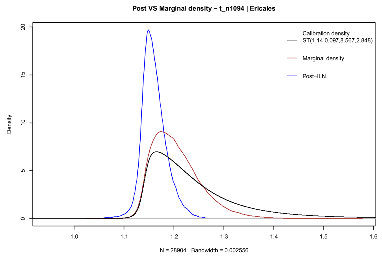
Post VS Marginal density – t_n1094 | Ericales
Calibration density
ST(1.14,0.097,8.567,2.848)
Marginal density
Post−ILN
Density
N = 28904 Bandwidth = 0.002556

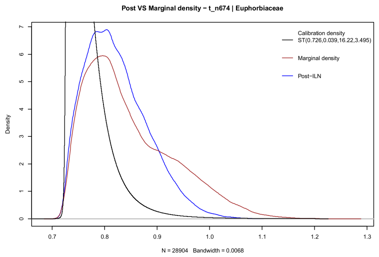
Post VS Marginal density – t_n674 | Euphorbiaceae
Calibration density
ST(0.726,0.039,16.22,3.495)
Marginal density
Post−ILN
Density
N = 28904 Bandwidth = 0.0068

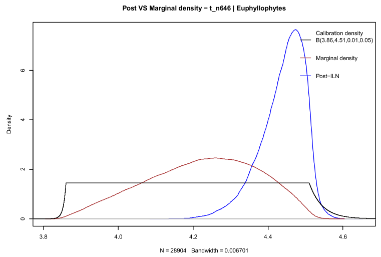
Post VS Marginal density – t_n646 | Euphyllophytes
Calibration density
B(3.86,4.51,0.01,0.05)
Marginal density
Post−ILN
Density
N = 28904 Bandwidth = 0.006701

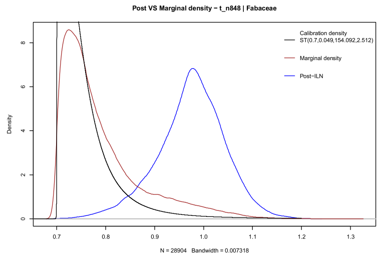
Post VS Marginal density – t_n848 | Fabaceae
Calibration density
ST(0.7,0.049,154.092,2.512)
Marginal density
Post−ILN
Density
N = 28904 Bandwidth = 0.007318

Post VS Marginal density − t_n847 | Fabales

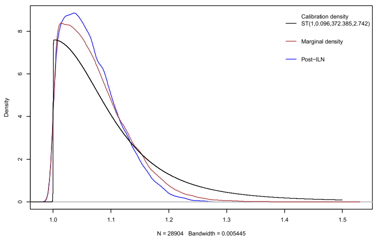

Post VS Marginal density − t_n826 | Fagaceae

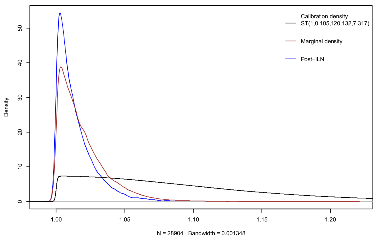

Post VS Marginal density − t_n825 | Fagales

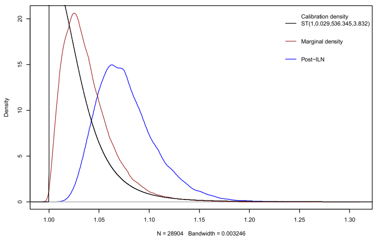

Post VS Marginal density − t_n1286 | Ferns

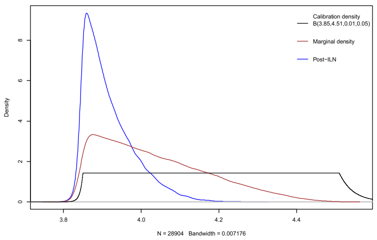

Post VS Marginal density − t_n1093 | Garryales

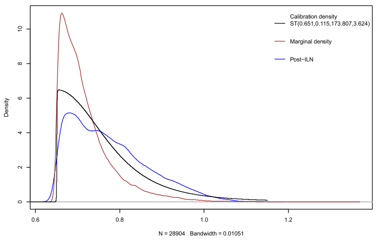

Post VS Marginal density − t_n1083 | Gentianales

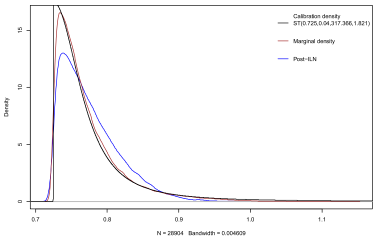

Post VS Marginal density − t_n1284 | Ginkgo−Cycas

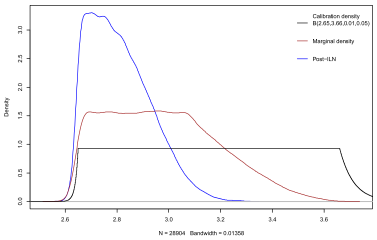

Post VS Marginal density − t_n1282 | Gnetales

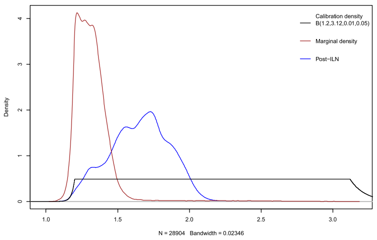

Post VS Marginal density − t_n906 | Haloragaceae

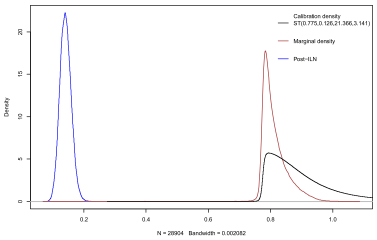

Post VS Marginal density − t_n914 | Hamamelidaceae

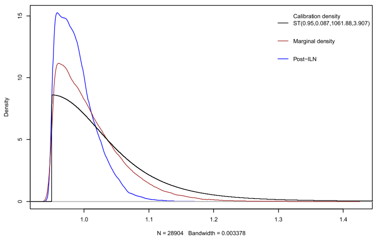

Post VS Marginal density − t_n791 | Humiriaceae

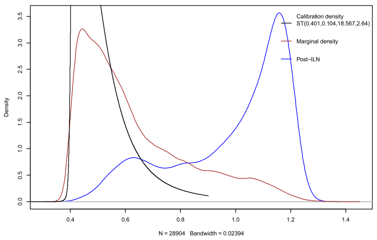

Post VS Marginal density − t_n779 | Hypericaceae

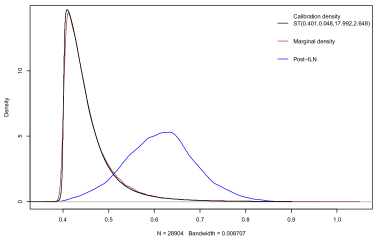

Post VS Marginal density − t_n1054 | Lamiales

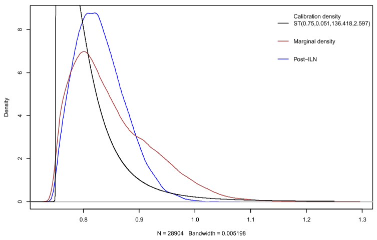

Post VS Marginal density − t_n1188 | Lardizabalaceae

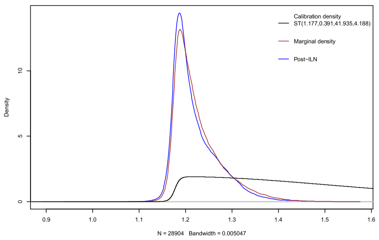

Post VS Marginal density − t_n1239 | Lauraceae

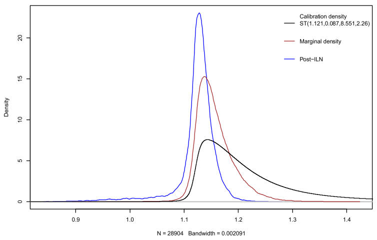

Post VS Marginal density − t_n1237 | Laurales

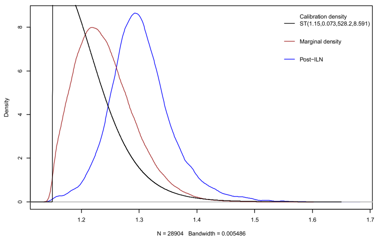

Post VS Marginal density − t_n1287 | Lycophytes

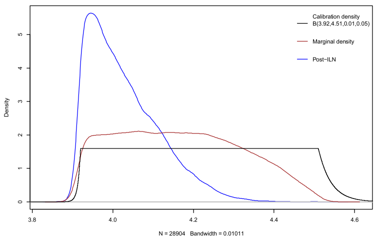

Post VS Marginal density − t_n1249 | Magnoliaceae

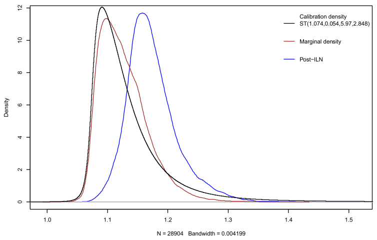

Post VS Marginal density − t_n1248 | Magnoliales

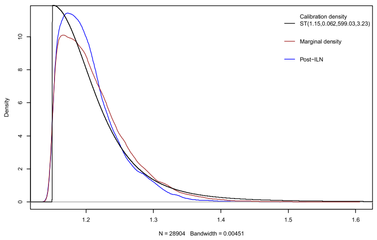

Post VS Marginal density − t_n697 | Malpighiaceae

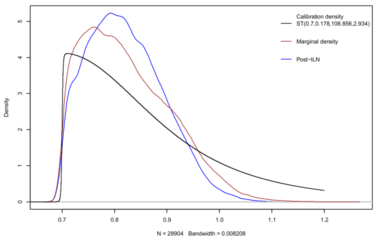

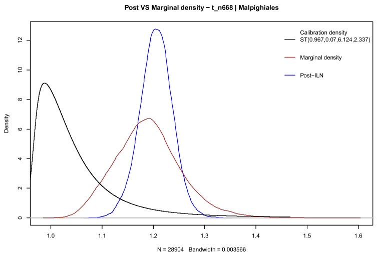
Post VS Marginal density − t_n668 | Malpighiales
Calibration density
ST(0.967,0.07,6.124,2.337)
Marginal density
Post−ILN
Density
N = 28904 Bandwidth = 0.003566

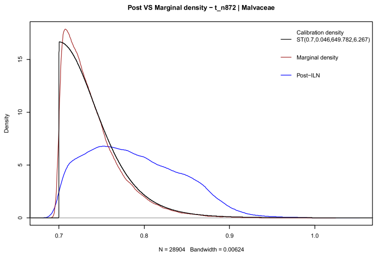
Post VS Marginal density − t_n872 | Malvaceae
Calibration density
ST(0.7,0.046,649.782,6.267)
Marginal density
Post−ILN
Density
N = 28904 Bandwidth = 0.00624

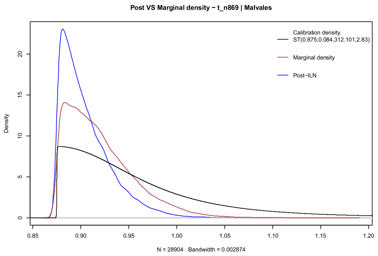
Post VS Marginal density − t_n869 | Malvales
Calibration density
ST(0.875,0.084,312.101,2.83)
Marginal density
Post−ILN
Density
N = 28904 Bandwidth = 0.002874

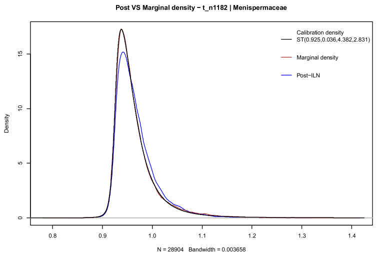
Post VS Marginal density − t_n1182 | Menispermaceae
Calibration density
ST(0.925,0.036,4.382,2.831)
Marginal density
Post−ILN
Density
N = 28904 Bandwidth = 0.003658

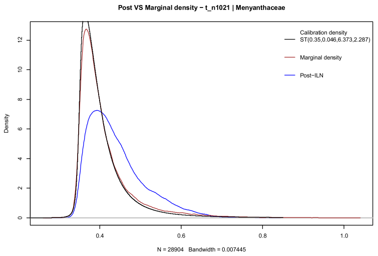
Post VS Marginal density − t_n1021 | Menyanthaceae
Calibration density
ST(0.35,0.046,6.373,2.287)
Marginal density
Post−ILN
Density
N = 28904 Bandwidth = 0.007445

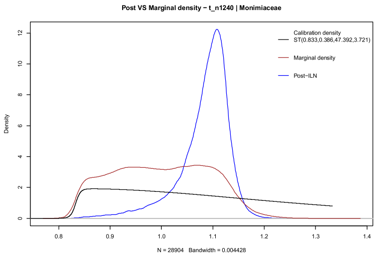
Post VS Marginal density − t_n1240 | Monimiaceae
Calibration density
ST(0.833,0.386,47.392,3.721)
Marginal density
Post−ILN
Density
N = 28904 Bandwidth = 0.004428

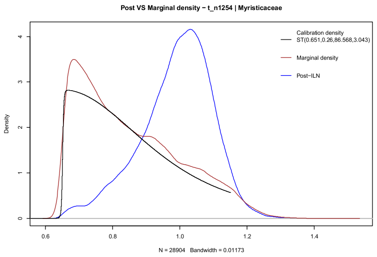
Post VS Marginal density − t_n1254 | Myristicaceae
Calibration density
ST(0.651,0.26,86.568,3.043)
Marginal density
Post−ILN
Density
N = 28904 Bandwidth = 0.01173

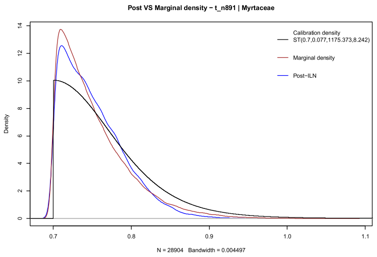
Post VS Marginal density − t_n891 | Myrtaceae
Calibration density
ST(0.7,0.077,1175.373,8.242)
Marginal density
Post−ILN
Density
N = 28904 Bandwidth = 0.004497

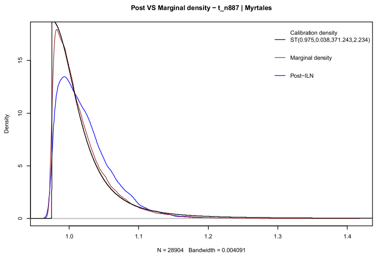
Post VS Marginal density − t_n887 | Myrtales
Calibration density
ST(0.975,0.038,371.243,2.234)
Marginal density
Post−ILN
Density
N = 28904 Bandwidth = 0.004091

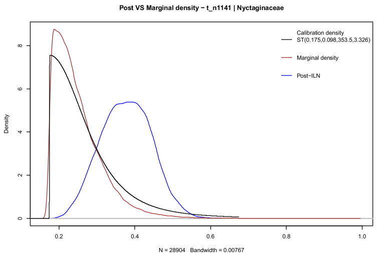
Post VS Marginal density − t_n1141 | Nyctaginaceae
Calibration density
ST(0.175,0.098,353.5,3.326)
Marginal density
Post−ILN
Density
N = 28904 Bandwidth = 0.00767

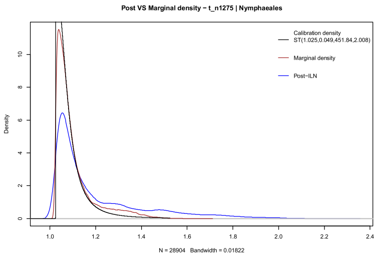
Post VS Marginal density − t_n1275 | Nymphaeales
Calibration density
ST(1.025,0.049,451.84,2.008)
Marginal density
Post−ILN
Density
N = 28904 Bandwidth = 0.01822

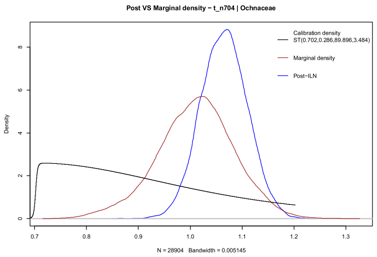
Post VS Marginal density − t_n704 | Ochnaceae
Calibration density
ST(0.702,0.286,89.896,3.484)
Marginal density
Post−ILN
Density
N = 28904 Bandwidth = 0.005145

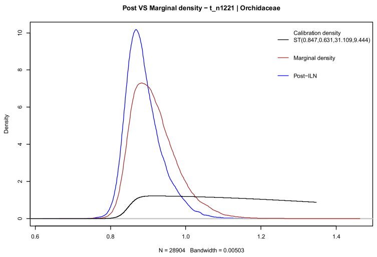
Post VS Marginal density − t_n1221 | Orchidaceae
Calibration density
ST(0.847,0.631,31.109,9.444)
Marginal density
Post−ILN
Density
N = 28904 Bandwidth = 0.00503

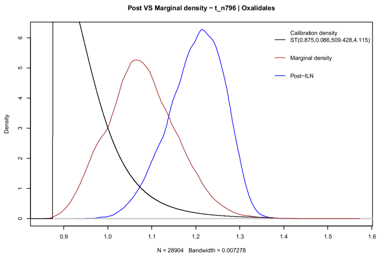
Post VS Marginal density − t_n796 | Oxalidales
Calibration density
ST(0.875,0.086,509.428,4.115)
Marginal density
Post−ILN
Density
N = 28904 Bandwidth = 0.007278

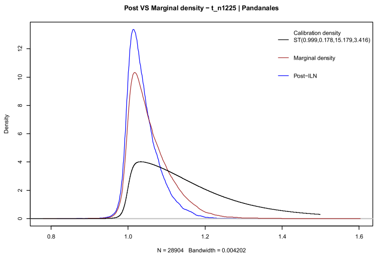
Post VS Marginal density − t_n1225 | Pandanales
Calibration density
ST(0.999,0.178,15.179,3.416)
Marginal density
Post−ILN
Density
N = 28904 Bandwidth = 0.004202

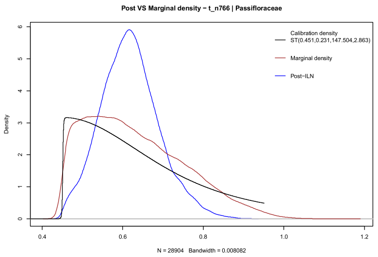
Post VS Marginal density − t_n766 | Passifloraceae
Calibration density
ST(0.451,0.231,147.504,2.863)
Marginal density
Post−ILN
Density
N = 28904 Bandwidth = 0.008082

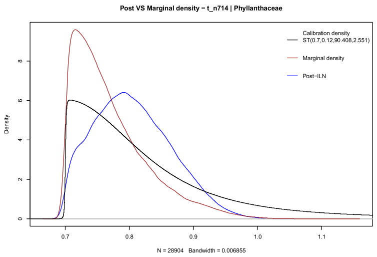
Post VS Marginal density − t_n714 | Phyllanthaceae
Calibration density
ST(0.7,0.12,90.408,2.551)
Marginal density
Post−ILN
Density
N = 28904 Bandwidth = 0.006855

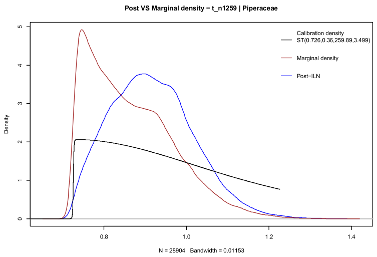
Post VS Marginal density − t_n1259 | Piperaceae
Calibration density
ST(0.726,0.36,259.89,3.499)
Marginal density
Post−ILN
Density
N = 28904 Bandwidth = 0.01153

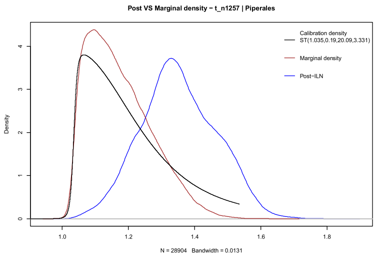
Post VS Marginal density − t_n1257 | Piperales
Calibration density
ST(1.035,0.19,20.09,3.331)
Marginal density
Post−ILN
Density
N = 28904 Bandwidth = 0.0131

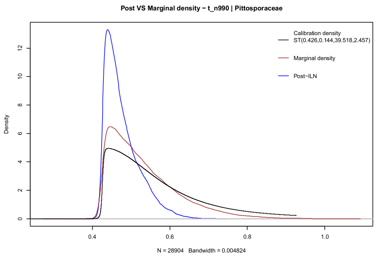
Post VS Marginal density − t_n990 | Pittosporaceae
Calibration density
ST(0.426,0.144,39.518,2.457)
Marginal density
Post−ILN
Density
N = 28904 Bandwidth = 0.004824

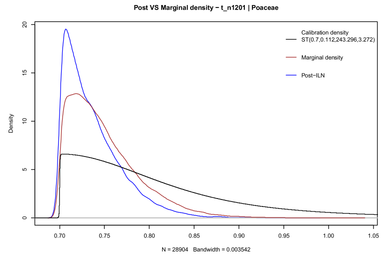
Post VS Marginal density ~ t_n1201 | Poaceae
Calibration density
ST(0.7,0.112,243.296,3.272)
Marginal density
Post−ILN
Density
N = 28904 Bandwidth = 0.003542

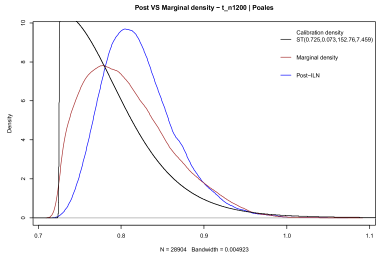
Post VS Marginal density ~ t_n1200 | Poales
Calibration density
ST(0.725,0.073,152.76,7.459)
Marginal density
Post−ILN
Density
N = 28904 Bandwidth = 0.004923

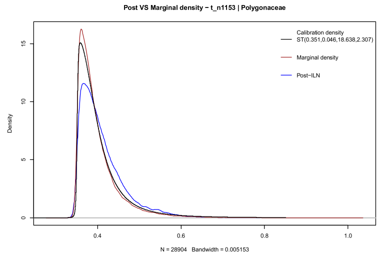
Post VS Marginal density ~ t_n1153 | Polygonaceae
Calibration density
ST(0.351,0.046,18.638,2.307)
Marginal density
Post−ILN
Density
N = 28904 Bandwidth = 0.005153

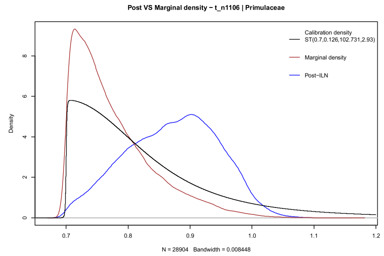
Post VS Marginal density ~ t_n1106 | Primulaceae
Calibration density
ST(0.7,0.126,102.731,2.93)
Marginal density
Post−ILN
Density
N = 28904 Bandwidth = 0.008448

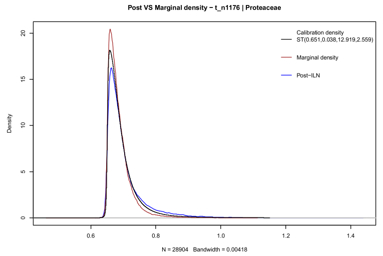
Post VS Marginal density ~ t_n1176 | Proteaceae
Calibration density
ST(0.651,0.038,12.919,2.559)
Marginal density
Post−ILN
Density
N = 28904 Bandwidth = 0.00418

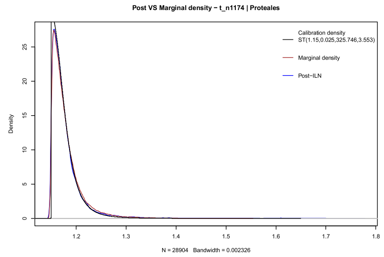
Post VS Marginal density ~ t_n1174 | Proteales
Calibration density
ST(1.15,0.025,325.746,3.553)
Marginal density
Post−ILN
Density
N = 28904 Bandwidth = 0.002326

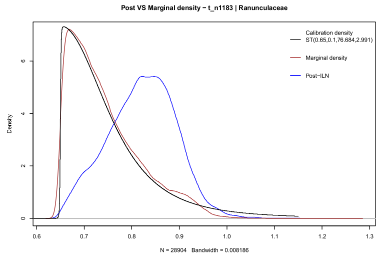
Post VS Marginal density ~ t_n1183 | Ranunculaceae
Calibration density
ST(0.65,0.1,76.684,2.991)
Marginal density
Post−ILN
Density
N = 28904 Bandwidth = 0.008186

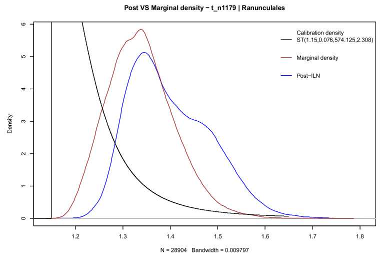
Post VS Marginal density ~ t_n1179 | Ranunculales
Calibration density
ST(1.15,0.076,574.125,2.308)
Marginal density
Post−ILN
Density
N = 28904 Bandwidth = 0.009797

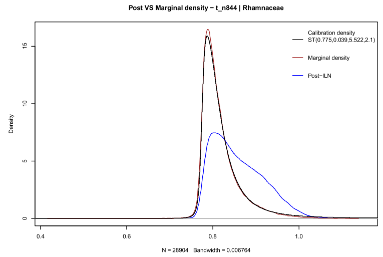
Post VS Marginal density ~ t_n844 | Rhamnaceae
Calibration density
ST(0.775,0.039,5.522,2.1)
Marginal density
Post−ILN
Density
N = 28904 Bandwidth = 0.006764

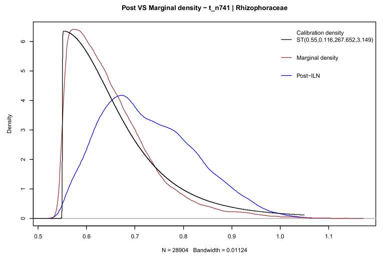
Post VS Marginal density ~ t_n741 | Rhizophoraceae
Calibration density
ST(0.55,0.116,267.652,3.149)
Marginal density
Post−ILN
Density
N = 28904 Bandwidth = 0.01124

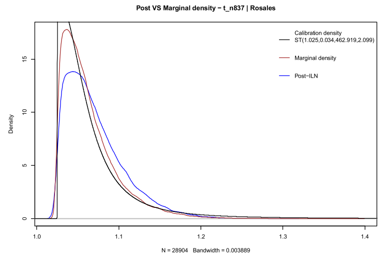
Post VS Marginal density ~ t_n837 | Rosales
Calibration density
ST(1.025,0.034,462.919,2.099)
Marginal density
Post−ILN
Density
N = 28904 Bandwidth = 0.003889

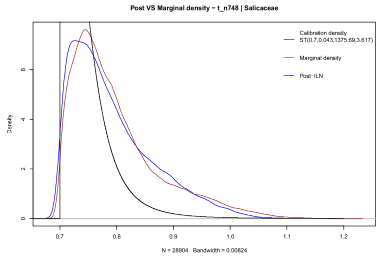
Post VS Marginal density ~ t_n748 | Salicaceae
Calibration density
ST(0.7,0.043,1375.69,3.617)
Marginal density
Post−ILN
Density
N = 28904 Bandwidth = 0.00824

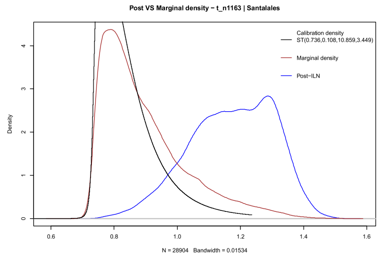
Post VS Marginal density ~ t_n1163 | Santalales
Calibration density
ST(0.736,0.108,10.859,3.449)
Marginal density
Post−ILN
Density
N = 28904 Bandwidth = 0.01534

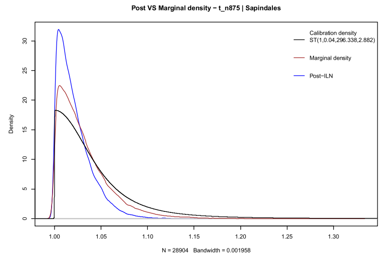
Post VS Marginal density ~ t_n875 | Sapindales
Calibration density
ST(1,0.04,296.338,2.882)
Marginal density
Post−ILN
Density
N = 28904 Bandwidth = 0.001958

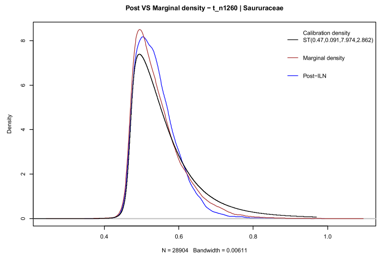
Post VS Marginal density ~ t_n1260 | Saururaceae
Calibration density
ST(0.47,0.091,7.974,2.862)
Marginal density
Post−ILN
Density
N = 28904 Bandwidth = 0.00611

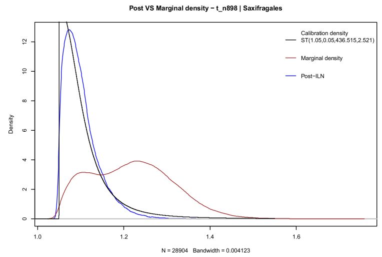
Post VS Marginal density ~ t_n898 | Saxifragales
Calibration density
ST(1.05,0.05,436.515,2.521)
Marginal density
Post−ILN
Density
N = 28904 Bandwidth = 0.004123

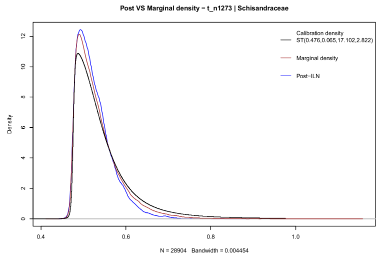
Post VS Marginal density ~ t_n1273 | Schisandraceae
Calibration density
ST(0.476,0.065,17.102,2.822)
Marginal density
Post−ILN
Density
N = 28904 Bandwidth = 0.004454

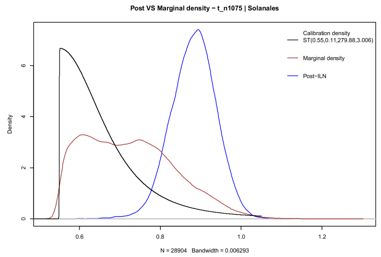
Post VS Marginal density ~ t_n1075 | Solanales
Calibration density
ST(0.55,0.11,279.88,3.006)
Marginal density
Post−ILN
Density
N = 28904 Bandwidth = 0.006293

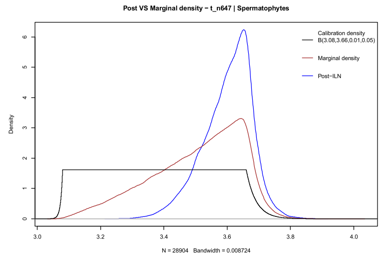
Post VS Marginal density ~ t_n647 | Spermatophytes
Calibration density
B(3.08,3.66,0.01,0.05)
Marginal density
Post−ILN
Density
N = 28904 Bandwidth = 0.008724

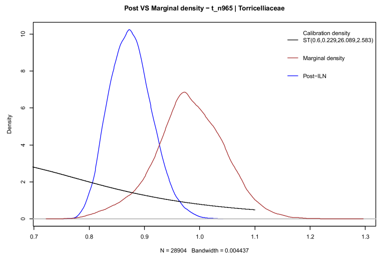
Post VS Marginal density ~ t_n965 | Torricelliaceae
Calibration density
ST(0.6,0.229,26.089,2.583)
Marginal density
Post−ILN
Density
N = 28904 Bandwidth = 0.004437

Post VS Marginal density − t_n645 | Tracheophyta

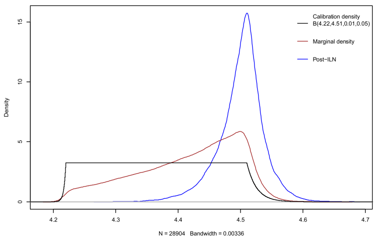

Post VS Marginal density − t_n1205 | Typhaceae

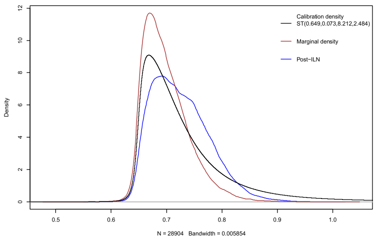

Post VS Marginal density − t_n841 | Urticaceae

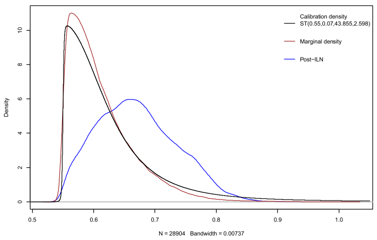

Post VS Marginal density − t_n762 | Violaceae

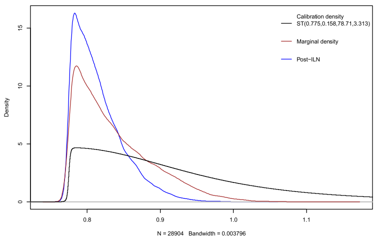

Post VS Marginal density − t_n897 | Vitaceae

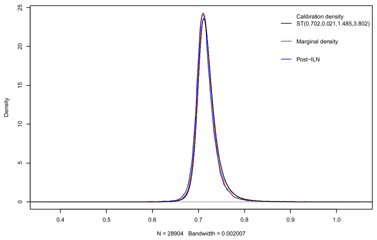

Post VS Marginal density − t_n1265 | Winteraceae

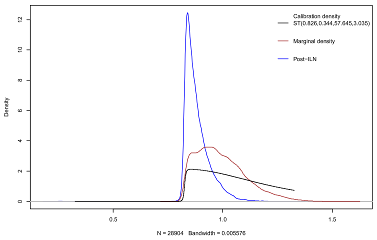

Post VS Marginal density − t_n1208 | Zingiberales

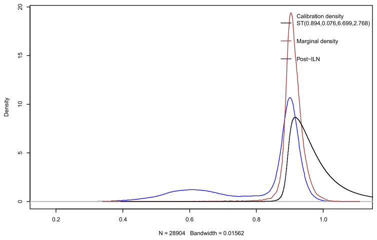

Supplementary Figure 4. Convergence plot

Convergence plot for posterior time densities inferred under the independent-rate log-normal relaxed-clock model (ILN) when uniform densities with hard bounds were used as calibration densities for constraining node ages.

A total of 32 independent chains were run under this strategy, and thus we calculated the mean posterior time densities for each node using the first 16 chains (x axis, 1-16) and then the last 16 chains (y axis, 17-32) to generate the convergence plot. Some of the chains did not pass our quality checks (i.e., differences in the tails of the distribution were larger than the threshold we specified) and were removed from the analysis. The plot "FILT" includes the chains that were kept and summarised to infer the corresponding evolutionary timeline. The straight line suggests that the different chains were sampling from the same target distribution and that it is likely that chains have reached convergence.

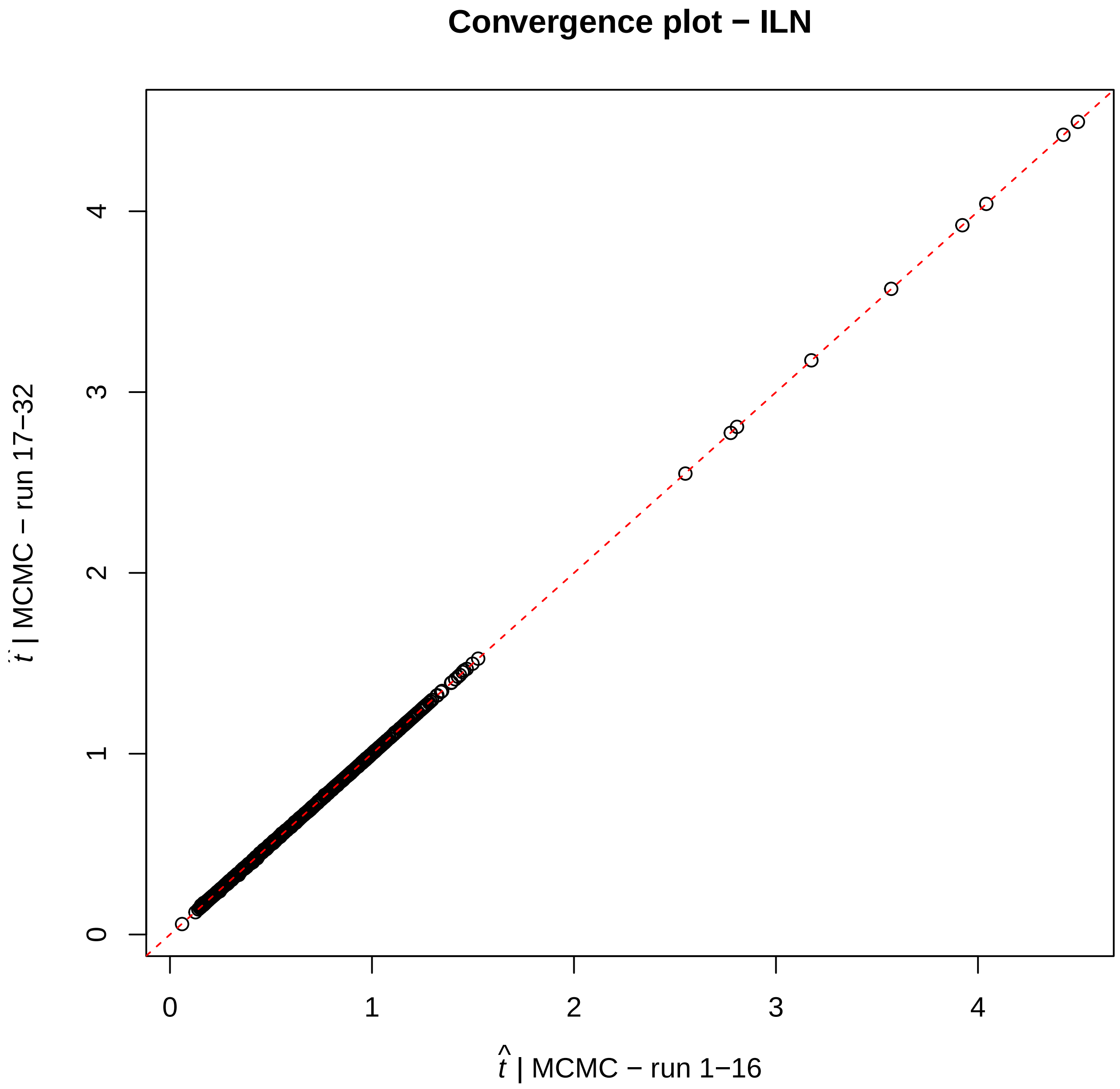

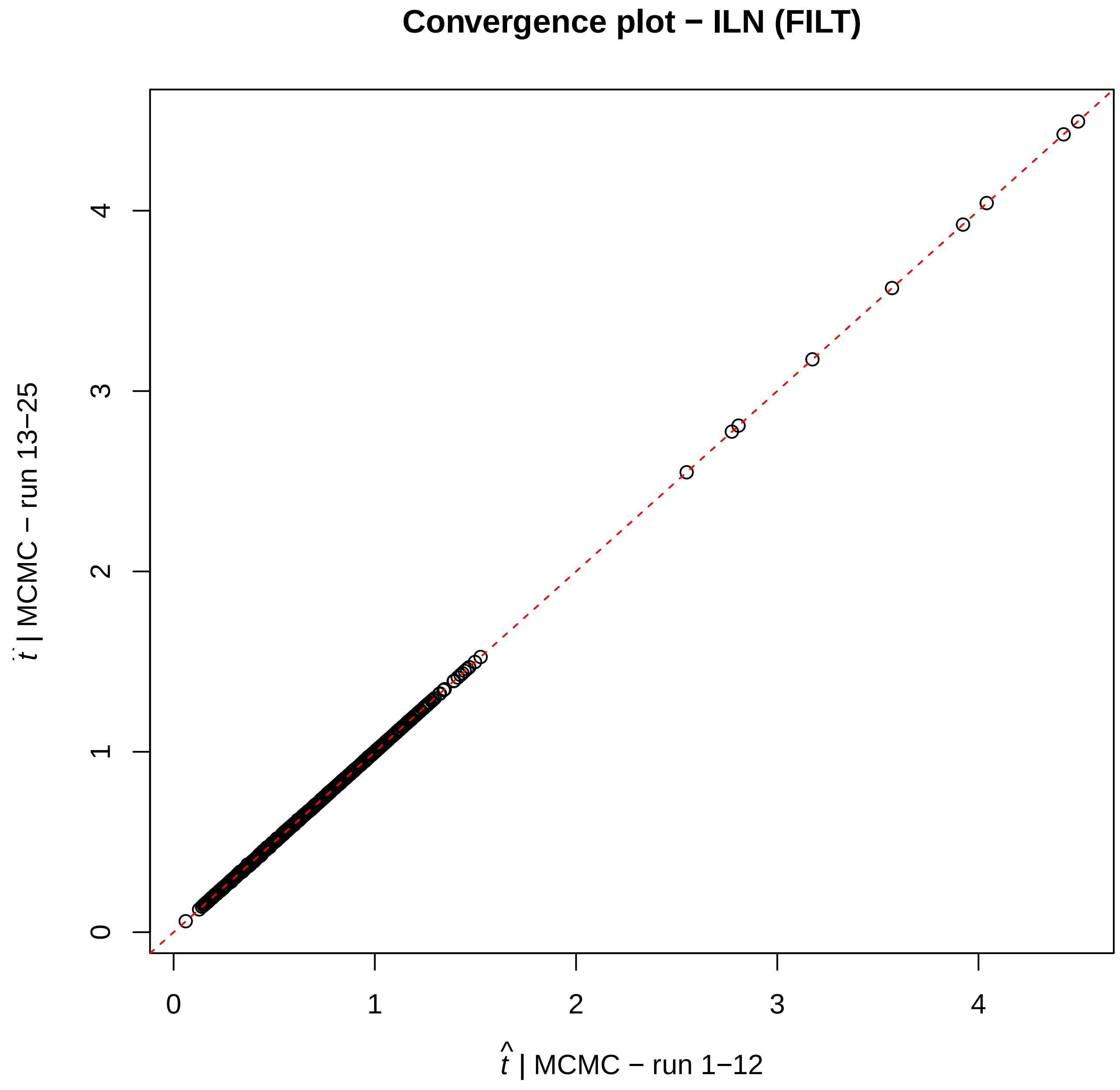
Convergence plot − ILN (FILT)
0
1
2
3
4
$\hat{t}$ | MCMC − run 1−12
$\ddot{t}$ | MCMC − run 13−25

Convergence plot for posterior time densities inferred under the independent-rate log-normal relaxed-clock model (ILN) when uniform densities with soft bounds were used as calibration densities for constraining node ages.

A total of 32 independent chains were run under this strategy, and thus we calculated the mean posterior time densities for each node using the first 16 chains (x axis, 1-16) and then the last 16 chains (y axis, 17-32) to generate the convergence plot. Some of the chains did not pass our quality checks (i.e., differences in the tails of the distribution were larger than the threshold we specified) and were removed from the analysis. The plot "FILT" includes the chains that were kept and summarised to infer the corresponding evolutionary timeline. The straight line suggests that the different chains were sampling from the same target distribution and that it is likely that chains have reached convergence.

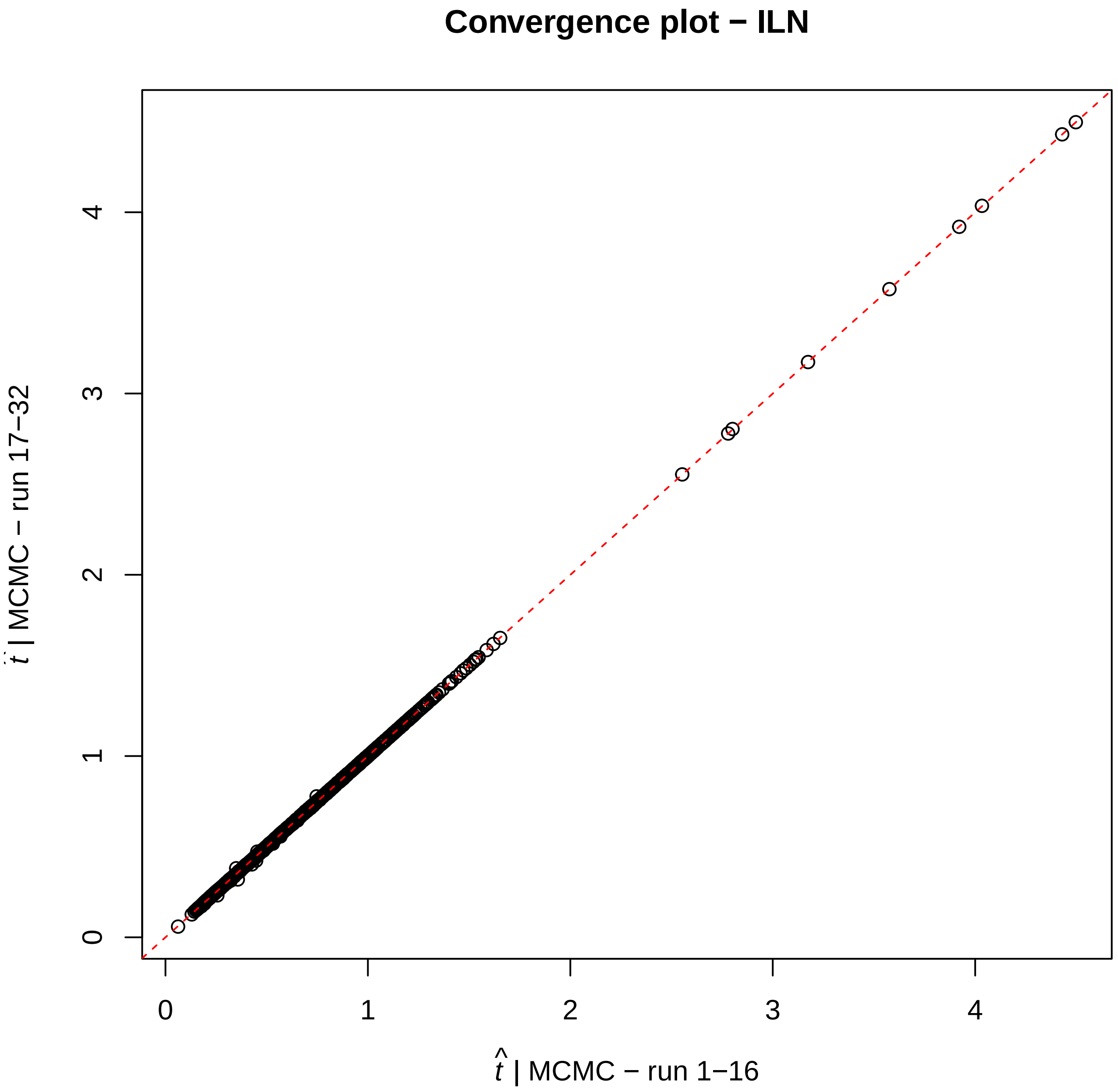

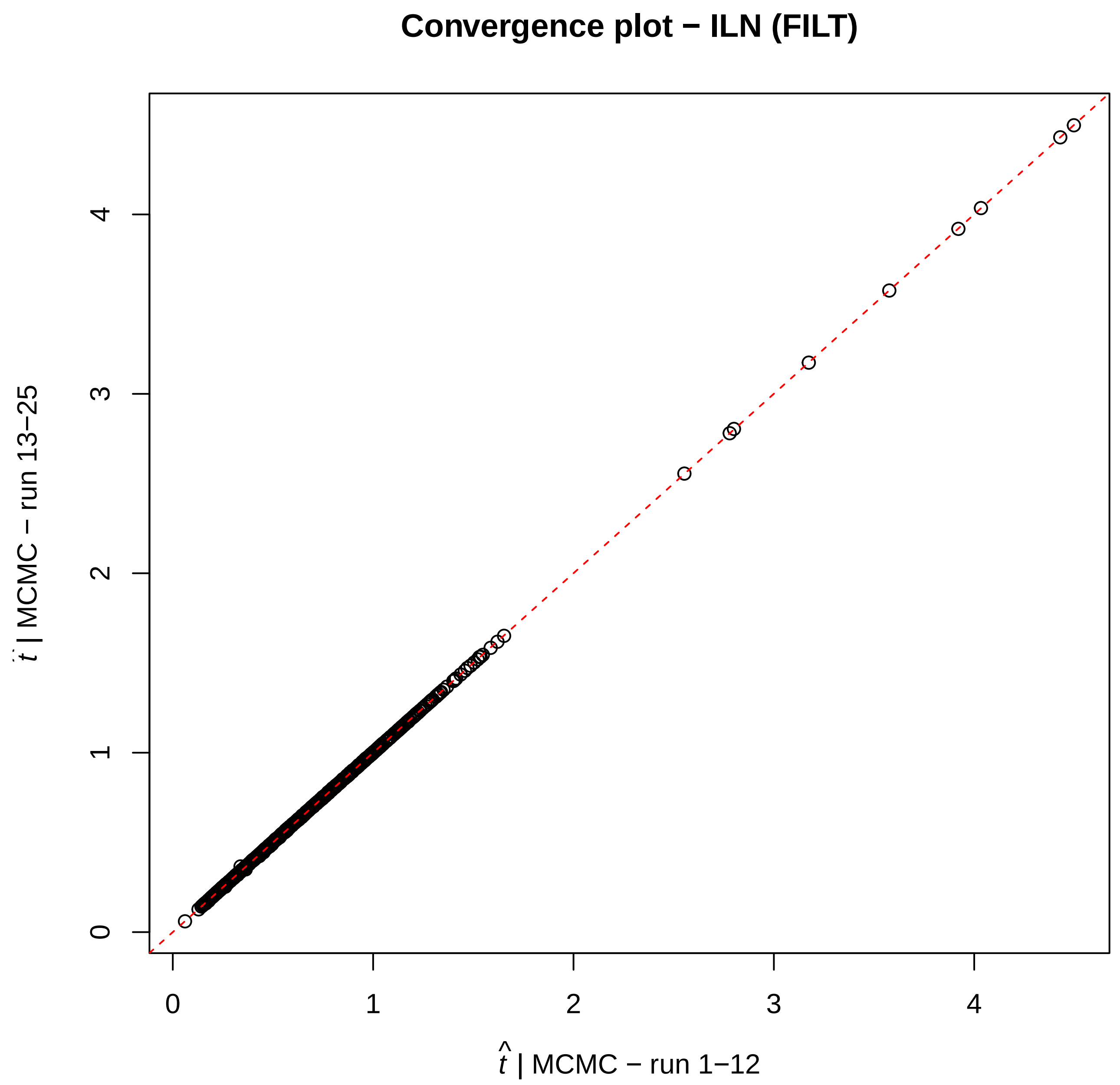

Convergence plot − ILN (FILT)
0
1
2
3
4
$\hat{t}$ | MCMC − run 1−12
$\ddot{t}$ | MCMC − run 13−25

Convergence plot for posterior time densities inferred under the independent-rate log-normal relaxed-clock model (ILN) when skew-T distributions were used as calibration densities for constraining node ages.

A total of 32 independent chains were run under this strategy, and thus we calculated the mean posterior time densities for each node using the first 16 chains (x axis, 1-16) and then the last 16 chains (y axis, 17-32) to generate the convergence plot. Some of the chains did not pass our quality checks (i.e., differences in the tails of the distribution were larger than the threshold we specified) and were removed from the analysis. The plot "FILT" includes the chains that were kept and summarised to infer the corresponding evolutionary timeline. The straight line suggests that the different chains were sampling from the same target distribution and that it is likely that chains have reached convergence.

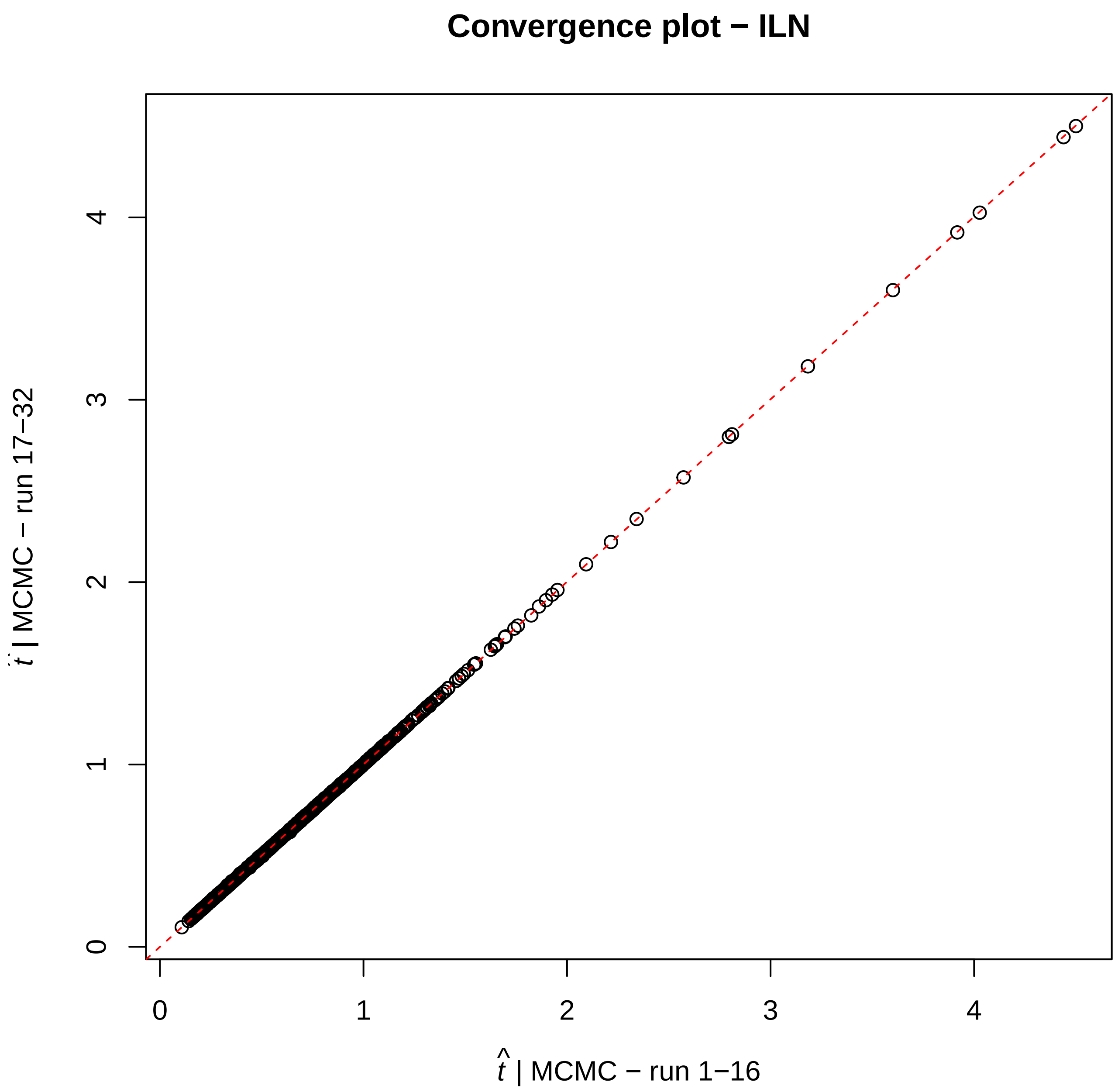

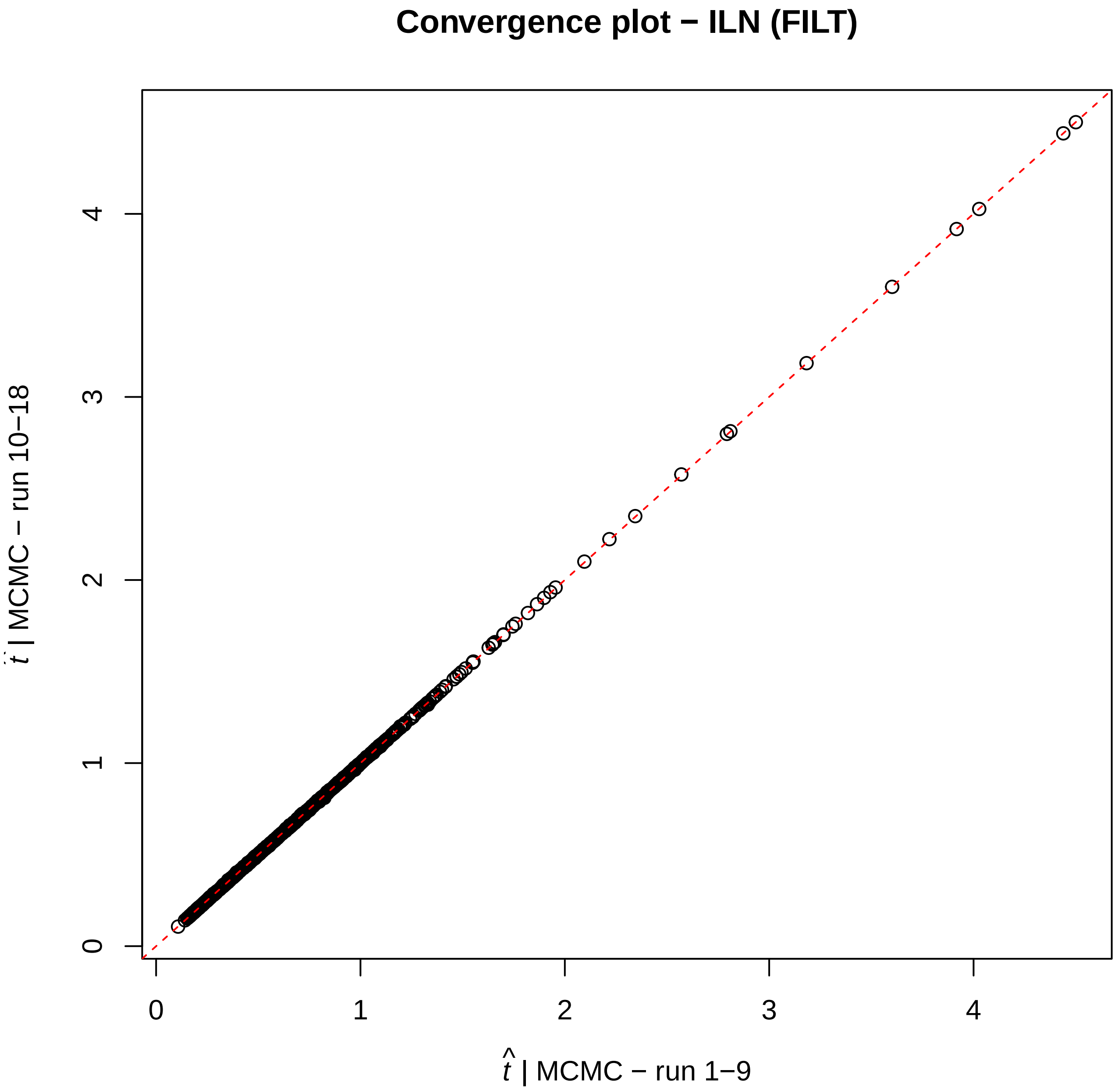
Convergence plot − ILN (FILT)
0
1
2
3
4
$\hat{t}$ | MCMC − run 1−9
$\ddot{t}$ | MCMC − run 10−18

Supplementary Figure 5. Estimated mean origin ages when including the full fossil dataset and two subsets (70% and 30% of the complete dataset).

(a) Mean origin ages for the angiosperm families Asteraceae and Fabaceae under three fossil sampling schemes: the full fossil dataset, 70% downsampling, and 30% downsampling. These two families represent clades with high extant diversity but contrasting fossil richness. Each point represents the mean of five independent subsampling replicates. Grey dots denote estimates from individual replicates, while the coloured bars show the average of the related replicates. The estimates derived from the full dataset are shown for comparison.

(b) 95% Highest Posterior Density (HPD) intervals for each downsampling level and the full dataset, and (c), 95% equal-tailed credible intervals corresponding to each downsampling scheme. Grey vertical lines indicate the range of values obtained of each replicate, while the coloured bars and dots show the average of all related replicates of the specific percentage.

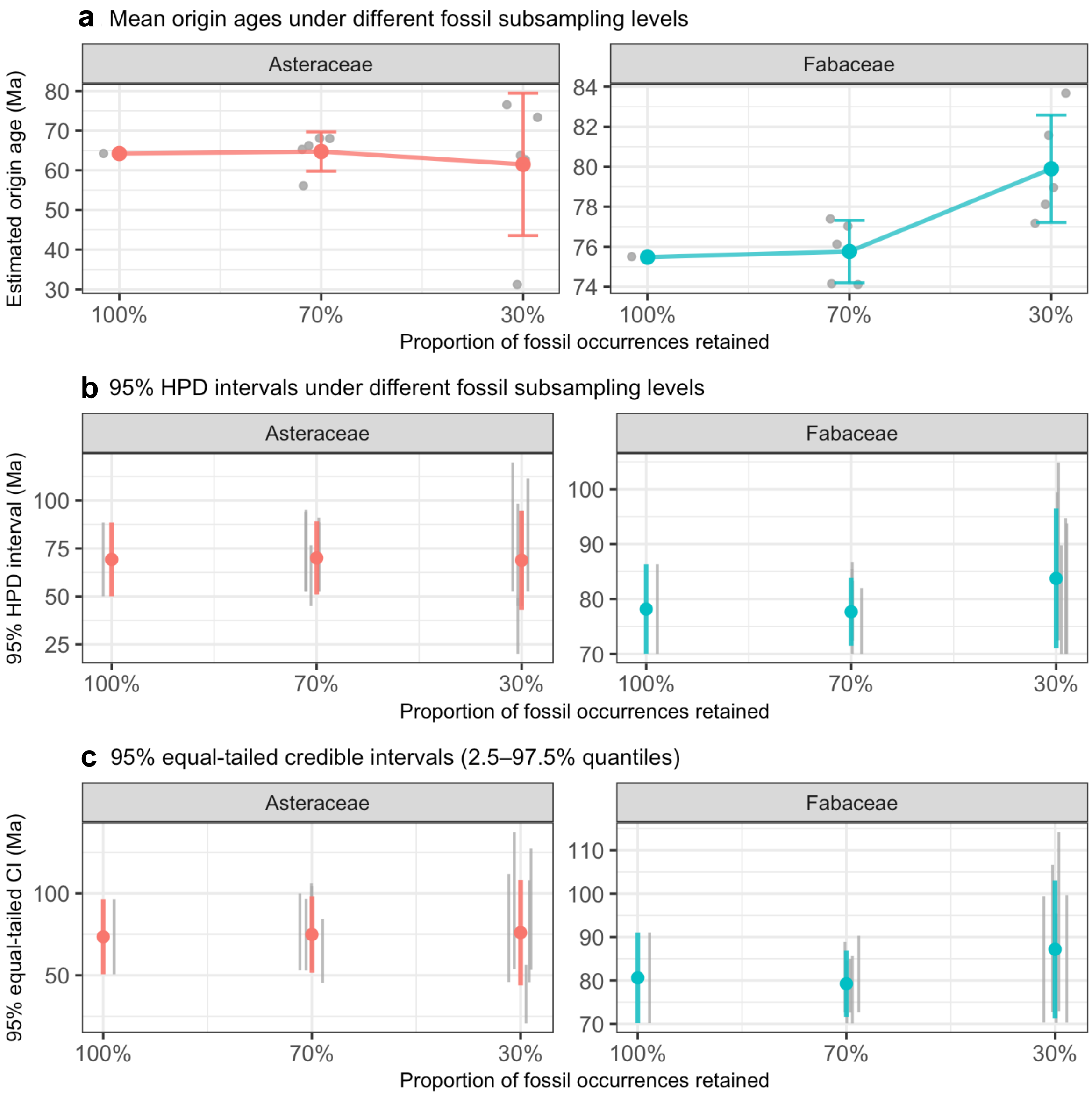

Supplementary Figure 6. Infinite-site plots calculated when using the full sequence alignment and various data subsets.

The latter were generated by randomly sampling 10%, 25%, 50%, and 75% of the sequences in the alignment (equivalent to including 10%, 25%, 50%, 75% of taxa).

Infinite-site plots for the full alignment and subsets when uniform densities with the hardbound strategy during timetree inference. For each dataset, the mean posterior times are shown in the x axis (time unit = Ma). The y axis illustrates the width of the corresponding mean posterior credible interval (CI) for each estimated node age. The legend included in each subplot shows the coefficient of determination for each comparison (R^2) and the equations of the regression lines with (red) and without (black) the root (node "Angiosperms").

a. Hard bound (100%)

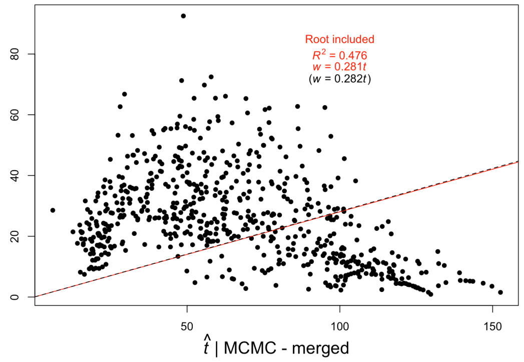

b. 10% subsample

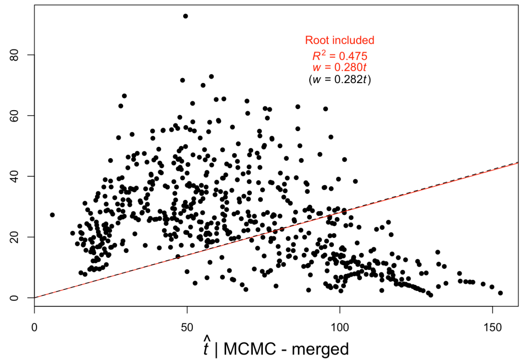

c. 25% subsample

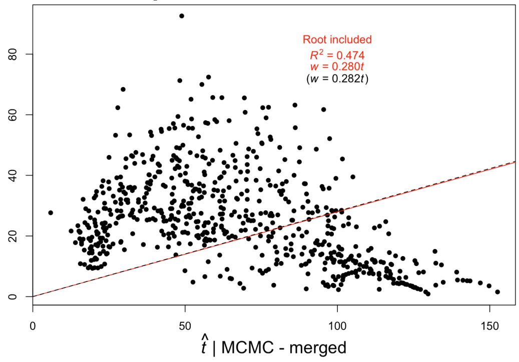

d. 50% subsample

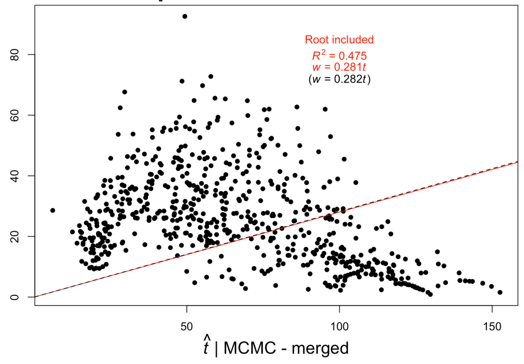

e. 75% subsample

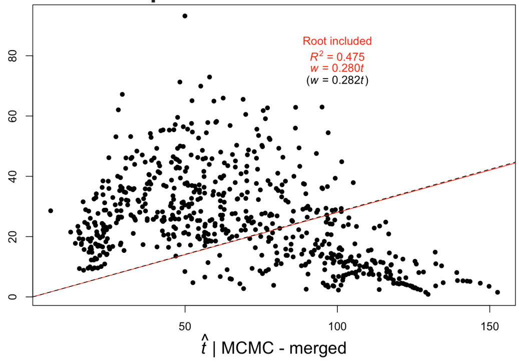

Infinite-site plots for each replicate with 10% randomly sampled sequences when the hardbound strategy was used during timetree inference. For each dataset, the mean posterior times are shown in the x axis (time unit = Ma). The y axis illustrates the width of the corresponding mean posterior credible interval (CI) for each estimated node age. The legend included in each subplot shows the coefficient of determination for each comparison (R^2) and the equations of the regression lines with (red) and without (black) the root (node "Angiosperms").

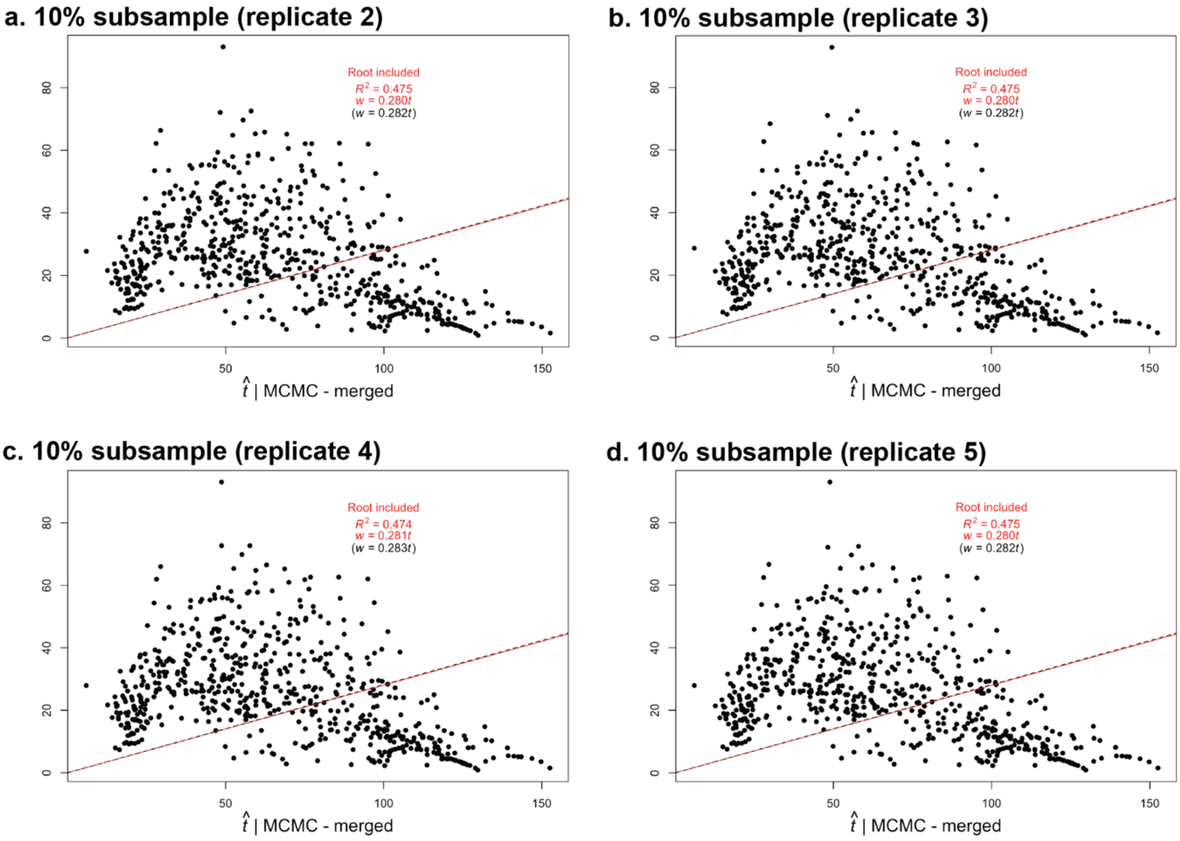

Infinite-site plots for each replicate with 25% randomly sampled sequences when the hardbound strategy was used during timetree inference. For each dataset, the mean posterior times are shown in the x axis (time unit = Ma). The y axis illustrates the width of the corresponding mean posterior credible interval (CI) for each estimated node age. The legend included in each subplot shows the coefficient of determination for each comparison (R^2) and the equations of the regression lines with (red) and without (black) the root (node "Angiosperms").

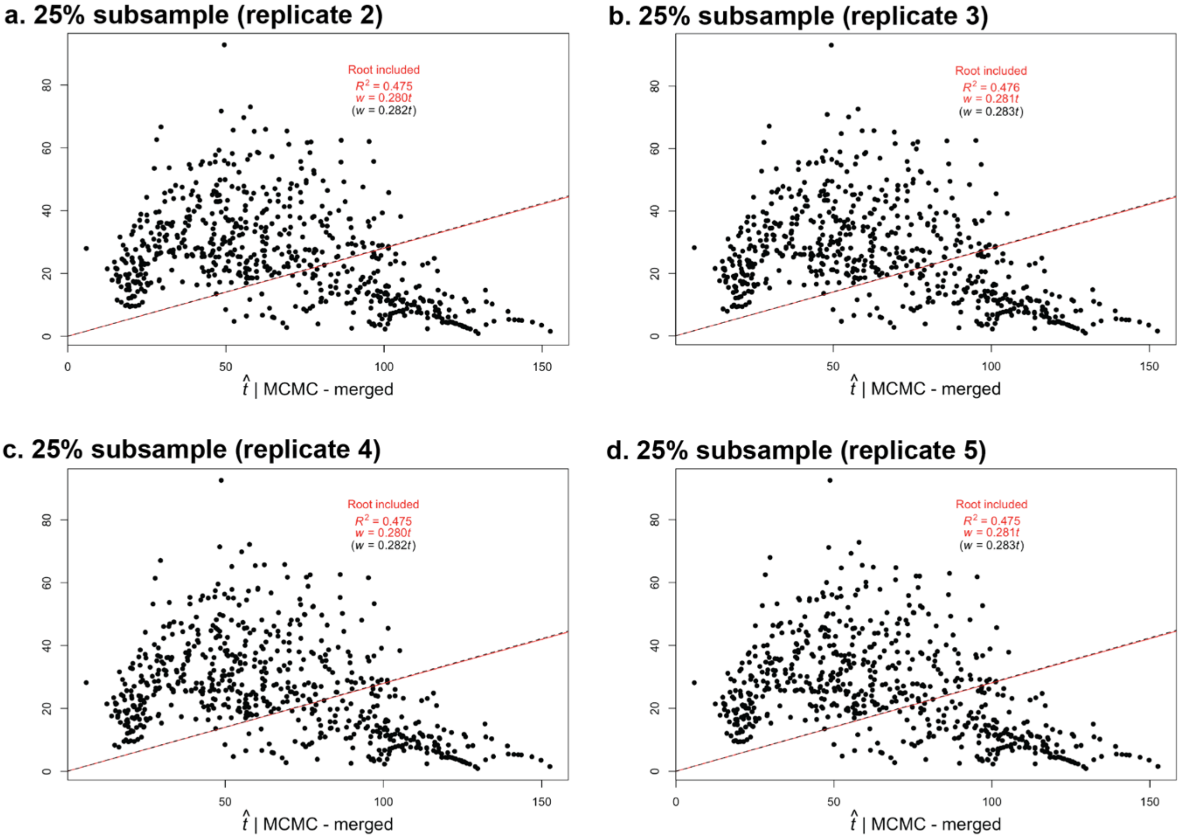

Infinite-site plots for each replicate with 50% randomly sampled sequences when the hardbound strategy was used during timetree inference. For each dataset, the mean posterior times are shown in the x axis (time unit = Ma). The y axis illustrates the width of the corresponding mean posterior credible interval (CI) for each estimated node age. The legend included in each subplot shows the coefficient of determination for each comparison (R^2) and the equations of the regression lines with (red) and without (black) the root (node "Angiosperms").

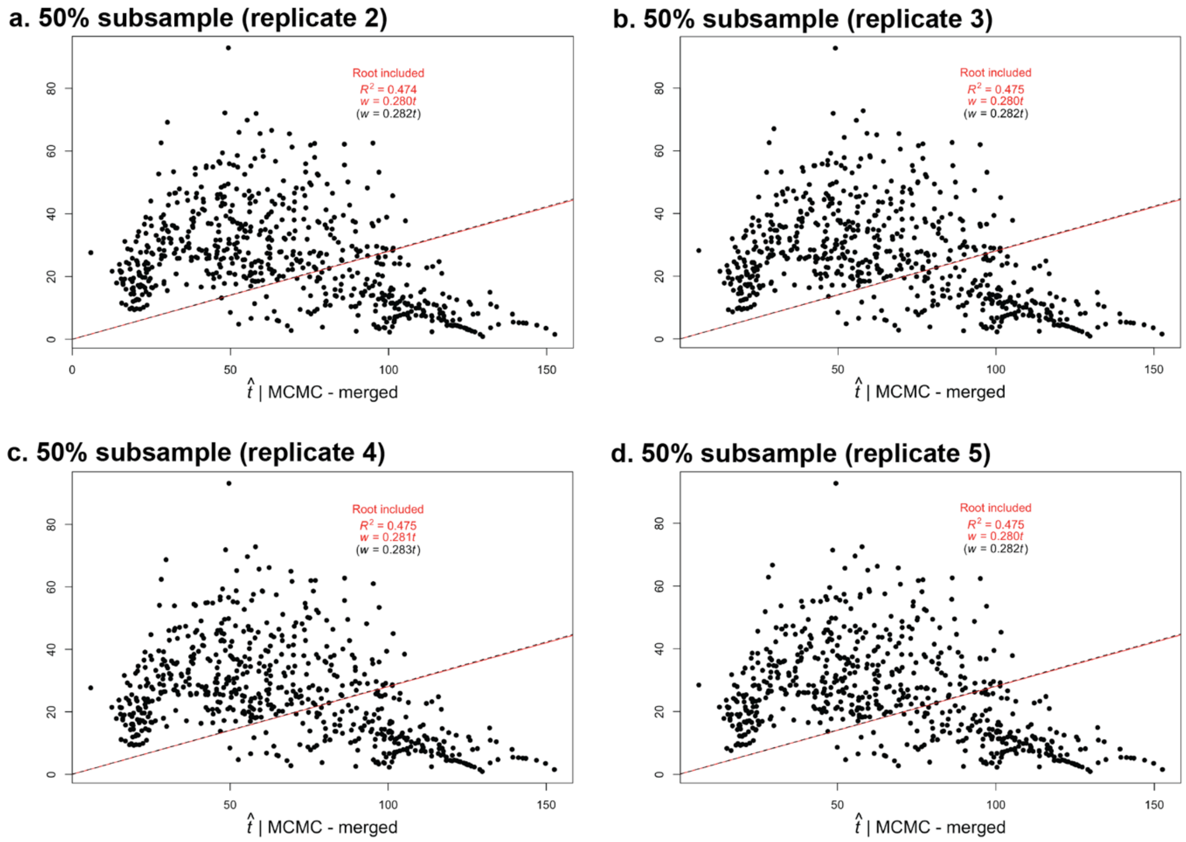

Infinite-site plots for each replicate with 75% randomly sampled sequences when the hardbound strategy was used during timetree inference. For each dataset, the mean posterior times are shown in the x axis (time unit = Ma). The y axis illustrates the width of the corresponding mean posterior credible interval (CI) for each estimated node age. The legend included in each subplot shows the coefficient of determination for each comparison (R^2) and the equations of the regression lines with (red) and without (black) the root (node "Angiosperms").

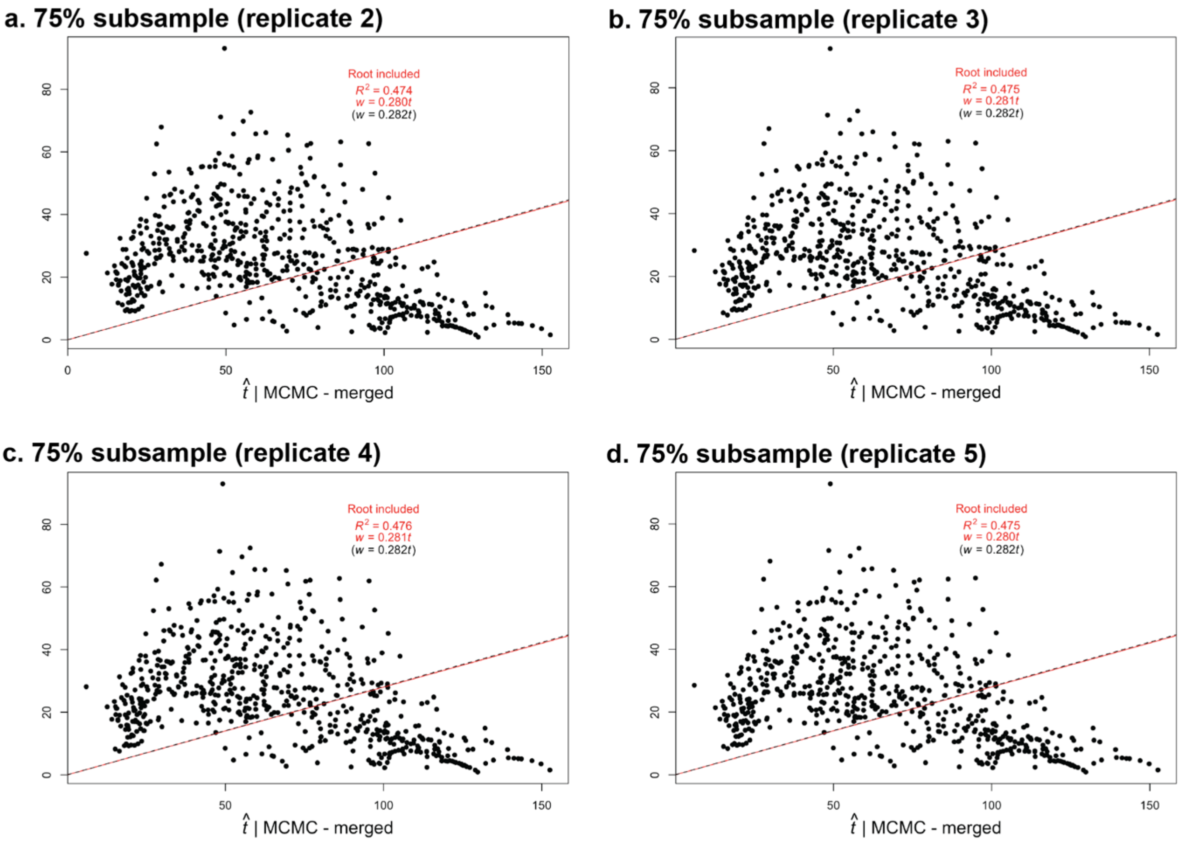

Supplement: Supplementary file 1 — Supplementary Sections 1–9. [file 41477_2026_2311_MOESM1_ESM.pdf]
